# Phase 2 study of pembrolizumab in patients with recurrent and residual high-grade meningiomas

**DOI:** 10.1038/s41467-022-29052-7

**Published:** 2022-03-14

**Authors:** Priscilla K. Brastianos, Albert E. Kim, Anita Giobbie-Hurder, Eudocia Quant Lee, Nancy Wang, April F. Eichler, Ugonma Chukwueke, Deborah A. Forst, Isabel C. Arrillaga-Romany, Jorg Dietrich, Zachary Corbin, Jennifer Moliterno, Joachim Baehring, Michael White, Kevin W. Lou, Juliana Larson, Magali A. de Sauvage, Kathryn Evancic, Joana Mora, Naema Nayyar, Jay Loeffler, Kevin Oh, Helen A. Shih, William T. Curry, Daniel P. Cahill, Fred G. Barker, Elizabeth R. Gerstner, Sandro Santagata

**Affiliations:** 1 Massachusetts General Hospital Cancer Center, Harvard Medical School, Boston, MA USA; 2 Dana-Farber Cancer Institute, Harvard Medical School, Boston, MA USA; 3 The Chenevert Family Brain Tumor Center, Smilow Cancer Hospital and Yale Cancer Center, Yale School of Medicine, New Haven, CT USA; 4 Wilmot Cancer Center, University of Rochester, Division of Neuro-Oncology, Rochester, NY USA; 5 Brigham and Women’s Hospital, Department of Pathology, Harvard Medical School, Boston, MA USA; 6 Ludwig Center at Harvard, Boston, MA USA

**Keywords:** CNS cancer, Cancer immunotherapy

## Abstract

High-grade meningiomas are associated with neuro-cognitive morbidity and have limited treatments. High-grade meningiomas harbor an immunosuppressive tumor microenvironment (TME) and programmed death-ligand 1 (PD-L1) expression may contribute to their aggressive phenotype. Here, we present the results of a single-arm, open-label phase 2 trial (NCT03279692) evaluating the efficacy of pembrolizumab, a PD-1 inhibitor, in a cohort of 25 evaluable patients with recurrent and progressive grade 2 and 3 meningiomas. The primary endpoint is the proportion of patients alive and progression-free at 6 months (PFS-6). Secondary endpoints include progression-free and overall survival, best intracranial response, and toxicity. Our study has met its primary endpoint and achieved a PFS-6 rate of 0.48 (90% exact CI: 0.31–0.66) and a median PFS of 7.6 months (90% CI: 3.4–12.9 months). Twenty percent of patients have experienced one (or more) grade-3 or higher treatment-related adverse events. These results suggest that pembrolizumab exerts promising efficacy on a subset of these tumors. Further studies are needed to identify the biological facets within the meningioma TME that may drive response to immune-based therapies.

## Abstract (web summary)

High-grade meningiomas have a poor prognosis with virtually no effective systemic therapies. Here, the authors report results of a phase 2 clinical trial demonstrating safety and activity of pembrolizumab, a PD-1 inhibitor, in patients with recurrent and residual high-grade meningiomas.

## Introduction

Meningiomas are the most common primary central nervous system (CNS) tumor, with a prevalence of 170,000 cases in the United States^1^. While most meningiomas are classified as World Health Organization (WHO) grade 1, a subset of these tumors cause significant neurologic morbidity and fatal complications. Higher-grade meningiomas (WHO grade 2 and 3) make up approximately 20% of all meningiomas^2^ and possess an aggressive phenotype, with 5-year recurrence rates of 50% for grade-2 (atypical) and 90% for grade-3 meningiomas. Despite aggressive treatments, prognoses for patients harboring these tumors are poor, with 10-year overall-survival (OS) rates of ~50% for grade-2 meningiomas and 0% for grade-3 meningiomas^2^.

The mainstay of treatment for meningiomas is maximal safe resection and, in some cases, adjuvant radiotherapy. Options for additional treatments, particularly for recurrent or higher-grade meningiomas, are limited due to a lack of effective systemic therapies. Traditional cytotoxic agents, hormonal therapy, and targeted agents have demonstrated modest benefit^3^. Response rates have been 0% in nearly all studies evaluating systemic therapy for recurrent meningiomas of all grades. Furthermore, many recurrent meningiomas do not have targetable genomic alterations or obvious drivers of malignant progression^4,5^, thus limiting the utility of targeted therapies.

Recent studies suggest that targeting the immune microenvironment may be a promising strategy for meningiomas^6–12^. Genomic-characterization studies suggest that a subset of high-grade meningiomas possess a high somatic mutation burden^9^. Accordingly, there is evidence that tumors with a high mutational load express immunogenic neoepitopes and may be responsive to immune-checkpoint inhibitors (ICI)^13^. Furthermore, work from our group has demonstrated the development of an immunosuppressive tumor microenvironment (TME) in higher-grade meningiomas. We found a decrease in CD4+ and CD8+ T cells, an increase in the number of FOXP3-expressing immunoregulatory (Treg) cells, and an increase in programmed death-ligand-1 (PD-L1) expression on tumor cells, compared with grade-1 meningiomas^14^. Others have identified that the presence of immunosuppressive myeloid cells in high-grade meningioma^11,12^, linked PD-L1/PD-L2 expression with worse outcomes^10,12^, and found enrichment of PD-1/PD-L1 signaling in malignant meningiomas by single-cell RNA sequencing^11^.

Based on these studies suggesting that high-grade meningiomas harbor an immunosuppressive TME, which may contribute to its aggressive phenotype, we hypothesize that pembrolizumab, a PD-1 inhibitor, would result in antitumor activity for high-grade meningiomas. Given that meningiomas grow outside the blood–brain barrier, coupled with extensive studies showing manageable adverse events in a number of solid tumors^15–18^, we have designed a prospective phase-2 study evaluating pembrolizumab, using a dose schedule that has demonstrated efficacy in systemic tumors, in patients with progressive high-grade meningiomas. Our results suggest that pembrolizumab exerts promising efficacy on a subset of these tumors and results in prolonged PFS compared with historical controls.

## Results

### Patients

Twenty-three patients had tissue-confirmed grade-2 meningioma, and three patients had grade-3 meningioma. Prior to enrollment, fifteen patients had progressive disease and eleven patients had residual measurable disease after surgery. Seven patients had either invasive (e.g., involving scalp or neck) or extracranial meningioma. Four patients had biopsy-proven distal metastatic disease (e.g., vertebral body, lung). Enrolled patients were first diagnosed with high-grade meningioma for a median of 11.5 months (range: 3–171) prior to enrollment. All patients underwent surgical resection, with a median of 2.5 prior intracranial surgeries (range: 1–5) per patient. Twenty-four patients received radiotherapy directed at the meningioma, with a median of 2 prior rounds of radiation (range: 1–4). Ten patients received systemic therapies prior to enrollment, with a median of 1 prior systemic therapy (range: 1–2).

#### Efficacy

**Fig. 1:**
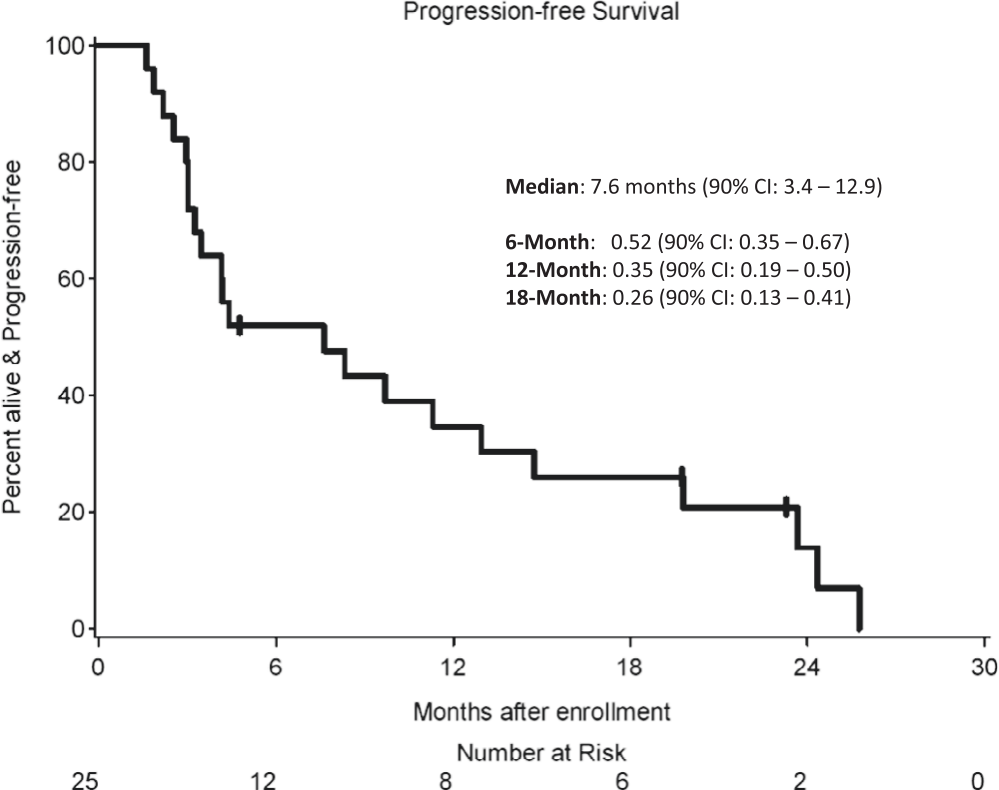
Kaplan–Meier curve for progression-free survival. Kaplan–Meier estimates of PFS are shown. There were 22 PFS events among 25 patients. The median PFS was 7.6 months (90% CI: 3.4–12.9).

**Fig. 2:**
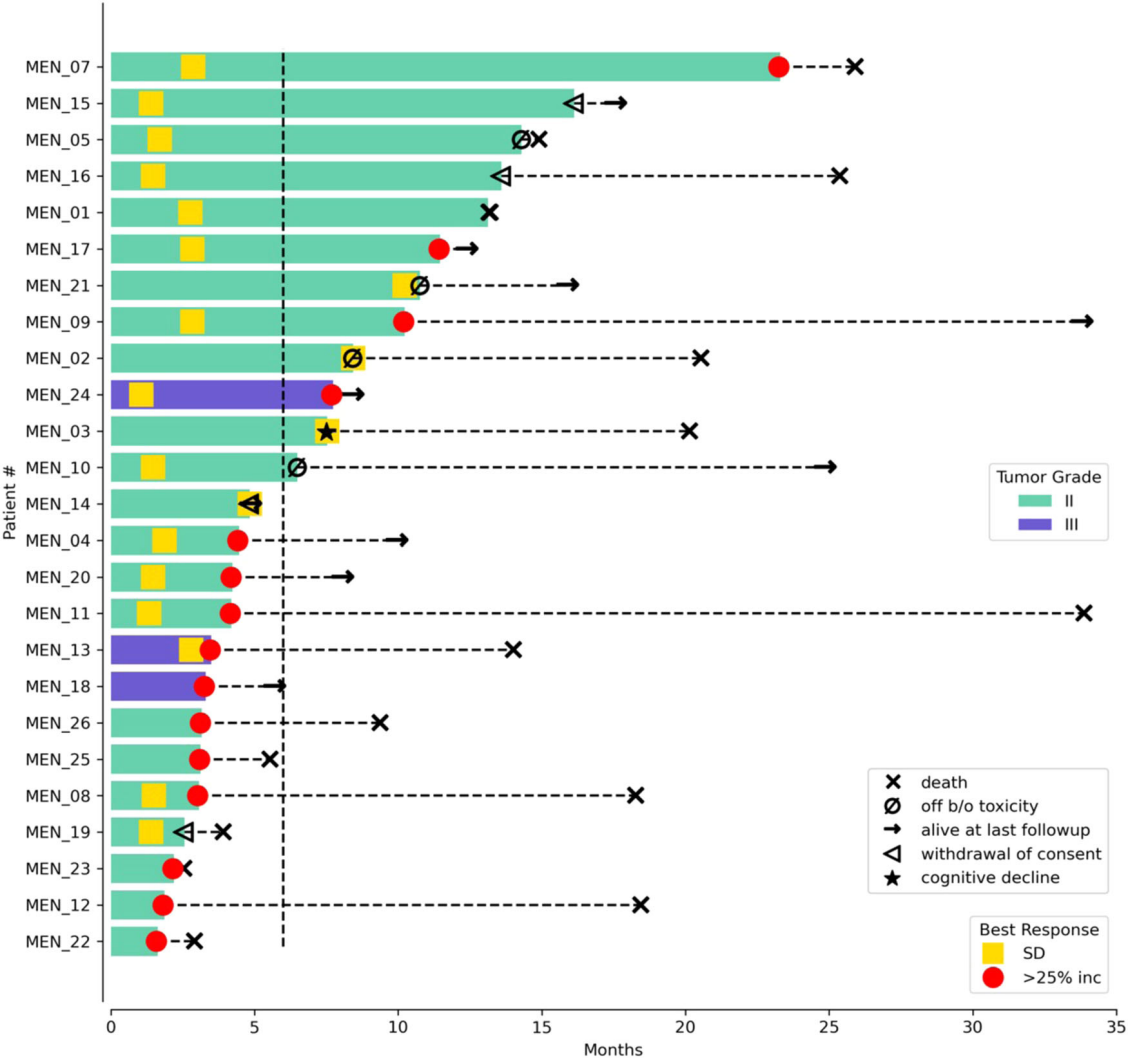
Clinical course of high-grade meningioma patients treated with pembrolizumab. Time of best radiographic response (as defined by TIMC), death, and study-limiting toxicity for the patient population. The arrow at the end of the bar indicates the time at which the patient was last known to be alive at the time of data analysis. The dashed line at 6 months is the threshold of the primary endpoint (PFS-6). Patients marked in blue had extracranial or metastatic meningiomas. One patient (MEN_06) was excluded from this schematic, as they withdrew consent one week after enrollment before receiving pembrolizumab.

As one patient withdrew consent before receiving pembrolizumab, the sample size for efficacy and safety analyses was 25 patients. The proportion of patients with PFS-6 was 0.48 (12/25, 90% exact CI: 0.31–0.66, Supplemental Table 2). Per prespecified criteria, the overall trial endpoint would be met if 10 or more patients had PFS-6; therefore, the study met the primary endpoint. The median PFS (Kaplan–Meier) for the entire cohort was 7.6 months (90% CI: 3.4–12.9 months, Fig. 1), and median OS was 20.2 months (90% CI: 14.8–25.8 months, Supplemental Fig. 2). For the twelve patients who achieved PFS-6, median PFS from the start of treatment was 17.3 months (90% CI: 9.7–24.3 months). Eleven of these patients possessed grade-2 meningiomas and one patient had a grade-3 meningioma (Supplemental Table 2). Two patients had PFS for longer than 24 months. Eighteen patients had RANO intracranial responses of SD as their best response. No patients had intracranial CR or PR (response rate [RR] = 0%, 90% CI: 0–11%). Figure 2 illustrates the time of best response for each patient and the overall clinical course for each patient. We calculated pre- and post-treatment growth rates for the fifteen patients that were enrolled due to PD. Six of these patients had a stabilization in their meningioma growth rate while on pembrolizumab, compared with their pretreatment growth arc (Supplemental Fig. 3). Furthermore, two patients had a small decrease in meningioma size, which did not quite meet the criteria for PR, while enrolled on trial. To evaluate whether prior systemic therapy might sensitize the meningioma to ICI, we evaluated the rate of PFS-6 for patients who underwent prior systemic therapy (33.3%, 3/9) and those without prior systemic therapy (56.3%, 9/16), and did not detect a statistically significant difference in PFS-6 (Fisher’s exact *p*-value: 0.41).

Of the seven patients with invasive or extracranial disease, four patients achieved PFS-6. One patient (MEN_15) with biopsy-confirmed vertebral body and lung metastases had PFS lasting for nearly twenty months. Additionally, three patients had extracranial SD and two patients had PD as their best response. Extracranial disease for two patients was unevaluable due to a lack of measurable extracranial lesions. Ten patients were still alive at the time of data-lock—the median follow-up for these patients was 22.8 months (interquartile range: 12.6–31.1). All patients were taken off pembrolizumab—the reasons for discontinuation include disease progression (15), withdrawal of consent (4), unacceptable toxicity (4), cognitive deterioration (1), and death unrelated to pembrolizumab due to a myocardial infarction from biopsy-confirmed coronary-artery disease (1).

#### Toxicity

The median number of cycles of pembrolizumab completed was 6 (range: 2–29). All 25 patients reported at least one AE (Supplemental Table 3). Ten patients (40%, 90% exact CI: 24–58%) had one or more grade-3 or higher AEs of any attribution. Twenty-two of 25 patients had one or more AEs that were at least possibly treatment-related (Supplemental Table 4). The most frequently occurring AEs were fatigue (*N* = 10) and pruritus (*N* = 6). Neurologic AEs were mostly grade 1–2, with the exception of one episode of grade-3 encephalopathy. Five patients (20%, 90% exact CI: 8–38%) had one or more grade-3 or higher AEs that were at least possibly treatment-related.

#### Biomarker studies

Eighteen patients had pretreatment tumor tissue available (Supplemental Fig. 4). We assessed PD-L1 expression using both conservative (PD-L1 > 0, MPS > 1) and stringent (PD-L1 2+; MPS > 5) thresholds. No significant correlation was observed between PD-L1 expression and outcome using either threshold (Supplemental Table 5). When the modified percent score (MPS) was assessed as a continuous scale, we found a nonsignificant trend of higher MPS in patients with PFS-6 (*p* = 0.13, Supplemental Table 6). Further, to assess whether PD-L1 expression may be associated with a stabilization in meningioma growth curve, we analyzed data from nine of the fifteen patients who were enrolled due to PD and for whom pretreatment tumor tissue was available. Using conservative thresholds for PD-L1 expression, all four patients with elevated PD-L1 expression had stabilization in their meningioma growth curve. Two of the five patients with low PD-L1 expression had stabilization in their growth curve. We found a nonsignificant trend between increased PD-L1 expression and stabilization in the growth curve (Fisher’s exact *p*-value: 0.17). There was no relationship between TIL density and achieving PFS-6.

Fourteen patients had meningiomas in areas that were reliably captured on the ADC–MRI sequence. The remaining patients had meningiomas in areas (e.g., high convexity) that were not well visualized on ADC–MRI. The Wilcoxon rank-sum analysis demonstrated a trend toward higher-median (*p* = 0.13) ADC values for patients with PFS-6. There was no relationship between heterogeneity of ADC values, as measured by CV, and outcome (Supplemental Fig. 5).

## Discussion

As a direct translation of our genomic-^4,5^ and TME^14^-characterization efforts, we evaluated the efficacy of pembrolizumab in recurrent and progressive high-grade meningiomas. Our study met its primary endpoint, demonstrating a PFS-6 rate of 0.48 in a heavily pretreated population. We chose PFS-6 to screen for treatment efficacy based on recommendations of a RANO working-group effort to homogenize benchmarks for clinical trials in treatment-refractory meningiomas^3^. We then calculated our target-sample size of 24 evaluable patients to determine a clinically significant effect based on a historical weighted average of PFS-6 rate of 26% from a meta-analysis using eleven prior trials evaluating systemic therapies for high-grade meningiomas^3^. While PFS-6 may not capture all the nuances of antitumor efficacy in this challenging population, this metric has value in serving as an initial screen to assess whether ICI may exert activity in these tumors.

Notably, our study reached its prespecified primary endpoint, despite one patient withdrawing consent before receiving treatment with pembrolizumab. Our study achieved a PFS-6 rate of 0.48 and median PFS of 7.6 months, which compare favorably to recent trials for high-grade meningiomas that report a median PFS of 4–26 weeks^3^. Observed responses in our cohort were durable, as the median PFS for the twelve patients who achieved PFS-6 was 17.3 months after enrollment. These results are even more compelling as many patients had exhausted all conventional or off-label treatments prior to enrollment and radiographic responses in meningioma are very uncommon. Finally, the toxicity profile of pembrolizumab was consistent with those of other studies of PD-1 inhibitors^15–18^, indicating that the majority of AEs can be managed with supportive care. Therefore, given limitations in treatments for recurrent high-grade meningiomas, our results suggest that patients with treatment-refractory meningiomas may benefit from ICI.

Interestingly, our trial enrolled seven patients with invasive or extracranial meningioma. Four of these patients achieved PFS-6 and one patient with distal metastatic disease had PFS lasting for nearly twenty months. Extracranial or metastatic meningiomas are exceedingly rare, with an estimated prevalence of 0.1–0.76% of all meningioma patients. Prognosis for this entity is especially poor, given aggressive histological features and the lack of effective systemic therapies^19^. Further compounding this issue is the fact that the presence of extracranial or metastatic disease can often be an exclusion criterion for meningioma trials. Given our findings, further exploration of pembrolizumab as a therapeutic option is warranted for patients with metastatic high-grade meningioma.

We performed molecular and imaging correlative analyses to identify biomarkers of response to pembrolizumab. While these analyses were hindered by our small sample size, we did find a nonsignificant trend in increased PD-L1 expression (through quantitative measurement) with likelihood of achieving PFS-6 and stabilization in meningioma growth rate. As with other tumors, these findings suggest that there may be a contribution of alternate TME factors, such as T-cell or myeloid-cell phenotypic dynamics, that dictate ICI response. On imaging analysis, we found a nonsignificant trend toward higher ADC values pretreatment and achieving PFS-6. Prior imaging studies have noted lower ADC values for meningiomas of high cellularity and high Ki-67 indices^20^, suggesting that meningiomas likely to derive benefit from pembrolizumab were in the less histologically aggressive subset of high-grade meningiomas. These results suggest that ADC–MRI may hold potential to noninvasively monitor meningioma physiology. However, because these findings did not meet statistical significance, future studies, with larger sample sizes, are needed to validate these findings and shed light on longitudinal physiology associated with ICI response and resistance.

Our study had some limitations. While our trial had a relatively small sample size, our patient enrollment and accrual rate were comparable to those in recent trials for high-grade meningiomas^3^. Second, our study did not possess a comparator arm through which to compare ICI to best physician practice. Many patients had already exhausted conventional treatments, and so, there were no further feasible standard-treatment options that could serve as an adequate control. Given our promising PFS-6 rate, our next steps will be to validate these results in larger randomized studies for recurrent meningiomas. Third, our cohort included seven patients with extracranial or metastatic meningioma. Given the rarity of these tumors and the likelihood that extracranial meningiomas possess different physiology than locally recurrent meningiomas confined to the CNS^19^, our cohort is somewhat heterogeneous and may not be representative of the general meningioma population. Nonetheless, given the promising efficacy of pembrolizumab for this diverse patient population, we feel that these results serve as a basis by which to evaluate ICI in larger cohorts of patients with high-grade meningiomas.

Additionally, while our PFS-6 rate was high, SD was the best-observed response. This was not unexpected as radiographic responses are very uncommon in high-grade meningiomas. Given the wide range of tumor volumes associated with SD (<50% decrease and <25% increase in tumor size) and rarity of meningioma response, we calculated pre-pembrolizumab and post-pembrolizumab tumor-growth and volume curves for a more detailed assessment of efficacy. On analysis of pre- and post-treatment tumor growth patterns, several patients had a stabilization in tumor growth during treatment with pembrolizumab. Furthermore, two patients had a small decrease in tumor size. Our hope is that pre- and post-treatment growth curves will be integrated in future meningioma trials as an outcome of interest. Furthermore, prolonged stability in patients with progressive surgery and radiation-refractory meningioma, an entity with a historically poor prognosis, is clinically relevant^3^.

Finally, we note that although all patients in our cohort were off treatment, ten patients were still alive at the time of data-lock. In a heavily pretreated cohort with minimal remaining standard treatments, this suggests a potential ongoing tumor-stabilization effect after ICI was stopped, which has been observed in meningioma^9^ and melanoma^21^. Similarly, it is also possible that treated tumors may show dramatic pseudoprogression due to marked inflammatory infiltrates^9^, which can meet the criteria for PD and curtail treatment. This phenomenon has been described with solid tumors of diverse histologies, including meningioma, undergoing treatment with ICI^22,23^. Further study is needed to fully understand these effects. Therefore, given the durability of PFS for the 12 patients that achieved the trial’s PFS-6 endpoint, immunosuppressive TME of high-grade meningiomas, and manageable toxicity of pembrolizumab, our data suggest that immunotherapy may have an emerging role in meningioma-treatment paradigms. The next steps include evaluating combination regimens, such as ICI with targeted therapies or novel therapies targeting immunosuppressive myeloid cells^8^, in larger studies that stratify between grade-2 and -3 meningioma. Given prior data that chemotherapy sensitizes solid tumors to ICI through augmenting dendritic-cell activation and abrogating regulatory T-cell and myeloid-derived suppressor-cell response^24^, future studies in which patients are stratified based on prior systemic treatments are warranted.

In summary, we conducted a phase-2 trial evaluating the efficacy of pembrolizumab in a cohort of heavily pretreated recurrent and progressive high-grade meningiomas. Our results suggest that pembrolizumab exerts promising activity on a subset of these tumors and results in prolonged PFS compared with historical controls. Further studies are needed to identify which meningioma subtypes or facets of the TME are associated with efficacy from immune-based therapies. While our trial met its primary endpoint, these results will require additional validation. Future studies of combination therapies, in conjunction with imaging and multi-omics analyses of these tumors, are needed to build upon these results.

## Methods

### Study oversight

The study (Clinicaltrials.gov identifier NCT03279692) was designed by the principal investigators and conducted in accordance with the provision of the Declaration of Helsinki and Good Clinical Practice guidelines. The Dana-Farber Harvard Cancer Center (DF/HCC) Institutional Review Board approved the protocol. All patients provided signed informed consent. Funding was provided by Merck.

### Patients

Between November 2017 and January 2021, 26 patients were enrolled (Supplemental Table 1). Eligible patients had histologically confirmed progressive or residual intracranial or metastatic grade-2 or -3 meningioma. Based on inclusion criteria for prior trials for treatment-refractory meningiomas and consensus RANO recommendations^3^, grade-2 and -3 meningiomas were included. Patients must have had progressive or residual measurable disease immediately prior to enrollment. Progressive disease was defined as an increase in size of a measurable meningioma on MRI by greater than 25% (bidirectional area) on scans separated by no more than 24 months. Residual measurable disease was defined by the presence of measurable disease, or a meningioma with clearly defined margins and a minimum diameter of 10 mm in one dimension, following surgery. Multifocal disease was allowed if one lesion met the criteria for measurable disease and progressive disease. Metastatic meningiomas, as defined by the presence of extracranial meningiomas, were allowed.

Prior meningioma-directed therapies, such as radiotherapy and systemic therapies, were allowed. To minimize the risk of enrolling patients with pseudoprogression, patients with prior external beam-radiation therapy or interstitial brachytherapy were required to show evidence of progressive meningioma within the irradiated field ≥ 24 weeks after completion of radiation treatment. Participants who had systemic therapies within 2 weeks prior to trial enrollment were excluded. Concurrent meningioma-directed systemic agents or radiation with the study drug was not allowed. Other key inclusion criteria included the following: age ≥18 years, ECOG-performance status ≤2, and stable dose of dexamethasone at 2 mg or less for at least 7 days prior to start of the trial. Key exclusion criteria included the presence of brainstem meningiomas and active autoimmune disease that required systemic immunosuppression within two years of enrollment.

### Study design, treatment, and endpoints

Pembrolizumab was administered intravenously at 200 mg IV every 3 weeks, until disease progression, death, or unacceptable toxicity. Dose reductions were not permitted; however, dose interruptions were allowed for adverse events (AEs) for up to 12 weeks. Treatment was resumed once AEs improved to grade 0–1 and corticosteroids (if started) were reduced to prednisone ≤10 mg or equivalent.

A brain MRI was obtained every 6 weeks for restaging. Intracranial and extracranial response was assessed centrally via blinded review by the MGH Tissue Imaging Metrics Core using modified Response Assessment in Neuro-oncology (RANO)^25^ and Response Evaluation Criteria in Solid Tumors (RECIST) 1.1^26^ criteria, respectively. The primary endpoint was the proportion of patients alive and progression-free at 6 months (PFS-6). All patients meeting the eligibility criteria that signed the consent form and began treatment with pembrolizumab were considered evaluable for the analysis of primary endpoint. PFS-6 was selected based on recommendations from a comprehensive RANO working-group effort that sought to define common benchmarks in clinical trials for meningiomas^3^. This meta-analysis noted that PFS-6 was the most consistently recorded endpoint for trials evaluating systemic therapies in surgery/radiation-refractory meningiomas and recommended using PFS-6 as a metric to measure efficacy for future trials. Radiographic response was evaluated as a secondary endpoint due to the lack of consensus radiographic-response criteria for meningiomas^3^.

The secondary endpoints include progression-free (PFS) and overall survival (OS), best intracranial response (as defined by complete response [CR], partial response [PR], stable disease [SD], or progressive disease [PD]), and toxicity using Common Terminology Criteria for Adverse Events (CTCAE) version 5.0. Under modified RANO criteria, CR was defined as the disappearance of all CNS target lesions. PR was a ≥50% decrease in the sum of longest diameters (LD) in CNS target lesions, relative to the baseline or nadir-sum LD, without new CNS lesions. This response must be sustained for at least four weeks, while on a stable corticosteroid dose. PD was defined as either a ≥25% increase in the sum LD of target lesions relative to baseline/nadir LD, appearance of new enhancing lesions or site of disease, or clear clinical decompensation (unless clearly unrelated to the meningioma—e.g., medication side effects, stroke, and infection). Stable disease (SD) was defined as a <50% decrease and <25% increase in the sum LD of target lesions relative to baseline/nadir LD, without new CNS lesions. As the vast majority of meningioma patients in prior trials have stable disease as their best response, alternate measures of treatment efficacy are needed. Therefore, we calculated the pretreatment and post-treatment growth rates of the meningioma, using the sum of LD in CNS target lesions, for patients enrolled due to progressive disease.

### Biomarker analysis

Archival meningioma-tissue samples, with a preference for tissue obtained immediately prior to enrollment, were collected. PD-L1 expression was centrally assessed at Discovery Life Sciences using the Merck 22C3 antibody (catalog number: SK006), a validated CLIA-certified assay for multiple solid tumors for both exploratory and prospective use, at a concentration of 2 micrograms/milliliter^27^. This assay has been used in a number of prior ICI-based trials^28–38^. The grading pathologist was blinded to patient response. Percentages of PD-L1-positive tumor and stromal cells were estimated based on all regions of the tumor to account for heterogeneous expression. Staining intensity was scored considering 0 as negative or trace, 1 as weak, 2 as moderate, and 3 as high. Moreover, a semiquantitative approach was also used to generate a score for each tissue section. The modified percent score (MPS), which ranges from 0 to 100, represented the overall percentage of both tumor and mononuclear inflammatory cells, which represent the stromal interface, that had membrane staining at low (1+) intensities or greater. As meningioma is not an approved indication for the 22C3-antibody assay, there was no preset threshold for PD-L1 positivity. Therefore, given the exploratory nature of this assay for meningiomas and variability in thresholds for PD-L1 positivity, we employed both conservative and liberal cutoffs for PD-L1 expression.

Density and distribution of tumor-infiltrating lymphocytes (TILs) on hematoxylin- and eosin-stained slides were estimated by a trained pathologist at Discovery Life Sciences using established criteria^39^ as performed in similar studies in other histologies^31,34,36^. The following scale was used: 0 for <1 TIL per high-power field (HPF) on average, 1 for 1–10 TIL per HPF on average, 2 for 11–20 TIL per HPF on average, and 3 for >20 TIL per HPF on average. This assessment included both stained and nonstained TILs. Therefore, the TIL scores represented the relative abundance of TILs for each case. To maximize objectivity for this assay, preset ranges, according to established criteria, for each value, were used for scoring. The value determined for each sample is based on averaging of abundance determined for the fields present in order to account for sample heterogeneity. A minimum of five 20x fields was required to make an assessment. Tissue molecular correlative analysis was exploratory and had no prespecified statistical plan for analysis.

For the imaging correlative studies, we focused our analysis on the ADC–MRI sequence. All patients underwent DWI-sequence acquisition with a 1.5T or 3T MRI scanner as part of routine brain-imaging protocols. The ADC maps were automatically generated from the DWI sequence. The ADC–MRI sequence was registered to the post-contrast MPRAGE MR sequence using the BRAINSFit module in 3D Slicer. A deep-learning algorithm (DeepNeuro) was used to initially segment the meningioma on the post-contrast MPRAGE images^40^. These regions of interest (ROIs) were reviewed and edited as needed (by ERG). Using normal white matter of the contralateral hemisphere, ADC voxel values within this ROI were then normalized as a ratio of ADC_meningioma_/ADC_white matter_. The mean- and median-normalized ADC voxel values were then calculated for each meningioma.

To assess the relationship between pretreatment ADC voxels and likelihood of PFS-6, we employed a nonparametric exact Wilcoxon rank-sum comparison using the median of the normalized ADC voxels for each patient. To quantitate heterogeneity of ADC voxels within the tumor ROI, we calculated the coefficient of variation (CV), which is defined as the standard deviation divided by the mean (SD/Mean). This metric summarized the extent of variability in relation to the average and was presented as a percent (100 * SD/Mean). CV was used to assess the relationship between heterogeneity of ADC voxels within the tumor ROI and likelihood of PFS-6.

### Statistical design

This clinical trial was designed as an open-label, single-arm phase-2 study (Supplemental Fig. 1). The total planned accrual was 26 patients to achieve at least 24 evaluable patients (i.e., received at least one dose of pembrolizumab). The trial was powered to distinguish between PFS-6 rates of 26% versus 52%. Prior studies have strongly suggested that patients with high-grade meningioma that progress after surgery and radiation and receive off-label or experimental systemic therapies have poor outcomes. Therefore, a recent RANO working group defined a PFS-6 threshold of 26% as clinically meaningful based on a meta-analysis of eleven prior trials that evaluated systemic therapies in surgery/radiation-refractory high-grade meningioma^3^. If at least 10 patients demonstrated PFS-6, among 24 evaluable patients, the agent would be considered worthy of further study. This design had at least 88% power (target 85%) to detect a true PFS-6 rate of at least 52%, using an exact binomial test with a one-sided significance level of 0.1, against the null hypothesis of 26%.

We summarized the proportion of patients who were alive, participated in follow-up, and progression-free at six months. Patients with progression or death, or whose follow-up was censored before six months, were counted as having events. The proportion of patients with intracranial response was summarized with a two-sided, 90% exact binomial confidence interval. Toxicities that were new or worsening relative to baseline are summarized according to the worst grade occurring for each patient. The distribution of OS and PFS was presented using the method of Kaplan–Meier with 90% confidence interval estimates using log(-log) methods. All statistical testing is two-sided; *p*-values are nominal. Stata version 16 was used for data analysis.

### Reporting summary

Further information on research design is available in the Nature Research Reporting Summary linked to this article.

### Supplementary information

Supplementary Information file

Reporting Summary

### Source data

Source Data

## Data Availability

The raw clinical and imaging data are protected due to patient privacy laws. Information is taken directly from the electronic medical record or original source generated by treating investigators (e.g., email confirmations, adverse event logs). This is stored on a secured network drive to which only appropriately trained and delegated staff have access to. Lesion measurements are obtained from the Tumor Metrics Imaging Core online portal that uses a secure server to which only appropriately trained and delegated staff are granted access to. Any requests for raw and analyzed data should be sent in writing to Priscilla Brastianos (pbrastianos@mgh.harvard.edu) and will be reviewed by the DF/HCC Institutional Review Board (IRB) in an expeditious fashion. Patient-related data not included in the paper were generated as part of a clinical trial and are subject to patient confidentiality. Any data and materials (e.g., tissue samples, PD-L1 testing, or imaging data) that can be shared will need approval from the DF/HCC IRB and a Material Transfer Agreement in place. Deidentified data will then be transferred to the inquiring investigator in an expeditious fashion over secure file transfer. The study protocol is available as Supplementary Note in the Supplementary Information file. Statistical-analysis plan and Source Data for the figures in this paper are included with the submission. The remaining data are available within the article and Supplementary Information. Source data are provided with this paper.

## References

[1] Ostrom QT (2020). CBTRUS statistical report: primary brain and other central nervous system tumors diagnosed in the United States in 2013–2017. Neuro. Oncol..

[2] Buerki RA (2018). An overview of meningiomas. Future Oncol..

[3] Kaley T (2014). Historical benchmarks for medical therapy trials in surgery-and radiation-refractory meningioma: a RANO review. Neuro. Oncol..

[4] Bi WL (2017). Genomic landscape of high-grade meningiomas. NPJ Genom. Med..

[5] Brastianos PK (2013). Genomic sequencing of meningiomas identifies oncogenic SMO and AKT1 mutations. Nat. Genet..

[6] Karimi, S. et al. Programmed death ligand-1 (PD-L1) expression in meningioma; prognostic significance and its association with hypoxia and NFKB2 expression. *Sci. Rep*. **10**, 14115 (2020). 10.1038/s41598-020-70514-z PMC7445252 32839486

[7] Everson RG (2018). Multiplatform profiling of meningioma provides molecular insight and prioritization of drug targets for rational clinical trial design. J. Neurooncol..

[8] Yeung J (2021). Spatially resolved and quantitative analysis of the immunological landscape in human meningiomas. J. Neuropathol. Exp. Neurol..

[9] Dunn, I. F. et al. Mismatch repair deficiency in high-grade meningioma: a rare but recurrent event associated with dramatic immune activation and clinical response to PD-1 blockade. *JCO Precis. Oncol*., 10.1200/po.18.00190 (2018). 10.1200/PO.18.00190 PMC6383717 30801050

[10] Han SJ (2016). Expression and prognostic impact of immune modulatory molecule PD-L1 in meningioma. J. Neurooncol..

[11] Blume, C. et al. Integrated phospho-proteogenomic and single-cell transcriptomic analysis of meningiomas establishes robust subtyping and reveals subtype-specific immune invasion. 10.1101/2021.05.11.443369 (2021).

[12] J., Yeung et al. OUP accepted manuscript. *Neuro. Oncol*., 10.1093/neuonc/noab075 (2021).

[13] Nebot-Bral L (2017). Hypermutated tumours in the era of immunotherapy: the paradigm of personalised medicine. Eur. J. Cancer.

[14] Du Z (2015). Increased expression of the immune modulatory molecule PDL1 (CD274) in anaplastic meningioma. Oncotarget.

[15] Powles T (2020). Pembrolizumab plus axitinib versus sunitinib monotherapy as first-line treatment of advanced renal cell carcinoma (KEYNOTE-426): extended follow-up from a randomised, open-label, phase 3 trial. Lancet Oncol..

[16] Mok TSK (2019). Pembrolizumab versus chemotherapy for previously untreated, PD-L1-expressing, locally advanced or metastatic non-small-cell lung cancer (KEYNOTE-042): a randomised, open-label, controlled, phase 3 trial. Lancet.

[17] Goldberg SB (2020). Pembrolizumab for management of patients with NSCLC and brain metastases: long-term results and biomarker analysis from a non-randomised, open-label, phase 2 trial. Lancet Oncol..

[18] Hamid O (2019). Five-year survival outcomes for patients with advanced melanoma treated with pembrolizumab in KEYNOTE-001. Ann. Oncol..

[19] Dalle Ore CL (2019). Meningioma metastases: incidence and proposed screening paradigm. J. Neurosurg..

[20] Surov A (2015). Diffusion-weighted imaging in meningioma: Prediction of tumor grade and association with histopathological parameters. Transl. Oncol..

[21] Larkin J (2019). Five-year survival with combined Nivolumab and Ipilimumab in advanced melanoma. N. Engl. J. Med..

[22] Chiou VL, Burotto M (2015). Pseudoprogression and immune-related response in solid tumors. J. Clin. Oncol..

[23] Park, H. J. et al. Incidence of pseudoprogression during immune checkpoint inhibitor therapy for solid tumors: a systematic review and meta-analysis. *Radiology*. **297**, 87–96 (2020). 10.1148/radiol.2020200443 PMC7526949 32749204

[24] Emens LA, Middleton G (2015). The interplay of immunotherapy and chemotherapy: harnessing potential synergies. Cancer Immunol. Res..

[25] Ellingson BM, Wen PY, Cloughesy TF (2017). Modified criteria for radiographic response assessment in glioblastoma clinical trials. Neurotherapeutics.

[26] Eisenhauer, E. A. et al. New response evaluation criteria in solid tumours: revised RECIST guideline (version 1.1). *Eur. J. Cancer*, 10.1016/j.ejca.2008.10.026 (2009). 10.1016/j.ejca.2008.10.026 19097774

[27] Dolled-Filhart M (2016). Development of a prototype immunohistochemistry assay to measure programmed death ligand-1 expression in tumor tissue. Arch. Pathol. Lab. Med..

[28] Garon EB (2015). Pembrolizumab for the treatment of non-small-cell lung cancer. N. Engl. J. Med..

[29] Muro K (2016). Pembrolizumab for patients with PD-L1-positive advanced gastric cancer (KEYNOTE-012): a multicentre, open-label, phase 1b trial. Lancet Oncol..

[30] Nanda, R. et al. Pembrolizumab in patients with advanced triple-negative breast cancer: phase Ib KEYNOTE-012 study. *J. Clin. Oncol*. **34**, 2460–2467 (2016). 10.1200/JCO.2015.64.8931 PMC6816000 27138582

[31] Adams S (2017). Dramatic response of metaplastic breast cancer to chemo-immunotherapy. NPJ Breast Cancer.

[32] R., Cristescu et al. Pan-tumor genomic biomarkers for PD-1 checkpoint blockade-based immunotherapy. *Science* **362**, eaar3593 (2018). 10.1126/science.aar3593 PMC6718162 30309915

[33] Joseph RW (2018). Baseline tumor size is an independent prognostic factor for overall survival in patients with melanoma treated with pembrolizumab. Clin. Cancer Res..

[34] Clouthier DL (2019). An interim report on the investigator-initiated phase 2 study of pembrolizumab immunological response evaluation (INSPIRE). J. Immunother. Cancer.

[35] Mehnert JM (2019). Safety and antitumor activity of the anti–PD-1 antibody pembrolizumab in patients with advanced, PD-L1–positive papillary or follicular thyroid cancer. BMC Cancer.

[36] Habra, M. A. et al. Phase II clinical trial of pembrolizumab efficacy and safety in advanced adrenocortical carcinoma. *J. Immunother. Cancer*. **7**, 253 (2019). 10.1186/s40425-019-0722-x PMC6751592 31533818

[37] Rodriguez CP (2020). A phase II trial of pembrolizumab and vorinostat in recurrent metastatic head and neck squamous cell carcinomas and salivary gland cancer. Clin. Cancer Res..

[38] Chung HC (2020). Pembrolizumab after two or more lines of previous therapy in patients with recurrent or metastatic SCLC: results from the KEYNOTE-028 and KEYNOTE-158 studies. J. Thorac. Oncol..

[39] Hendry S (2017). Assessing tumor-infiltrating lymphocytes in solid tumors: a practical review for pathologists and proposal for a standardized method from the International Immuno-Oncology Biomarkers Working Group: Part 2: TILs in melanoma, gastrointestinal tract carcinomas, non-small cell lung carcinoma and mesothelioma, endometrial and ovarian carcinomas, squamous cell carcinoma of the head and neck, genitourinary carcinomas, and primary brain tumors. Adv. Anat. Pathol..

[40] Beers, A. et al. DeepNeuro: an open-source deep learning toolbox for neuroimaging. *Neuroinformatics*, 10.1007/s12021-020-09477-5 (2020). 10.1007/s12021-020-09477-5 PMC7786286 32578020

