# Supplementary material for: Phase 2 study of pembrolizumab in patients with recurrent and residual high-grade meningiomas
Source: Nat Commun. 2022 Mar 14;13:1325. doi: 10.1038/s41467-022-29052-7 (PMC8921328; doi:10.1038/s41467-022-29052-7)
Supplement: Supplementary file 1 — Supplementary Information file [file 41467_2022_29052_MOESM1_ESM.pdf]

Supplemental Figure 1: Enrollment information. A total of 26 patients with high-grade meningiomas were consented and enrolled to the study between November 2017 to January 2021.

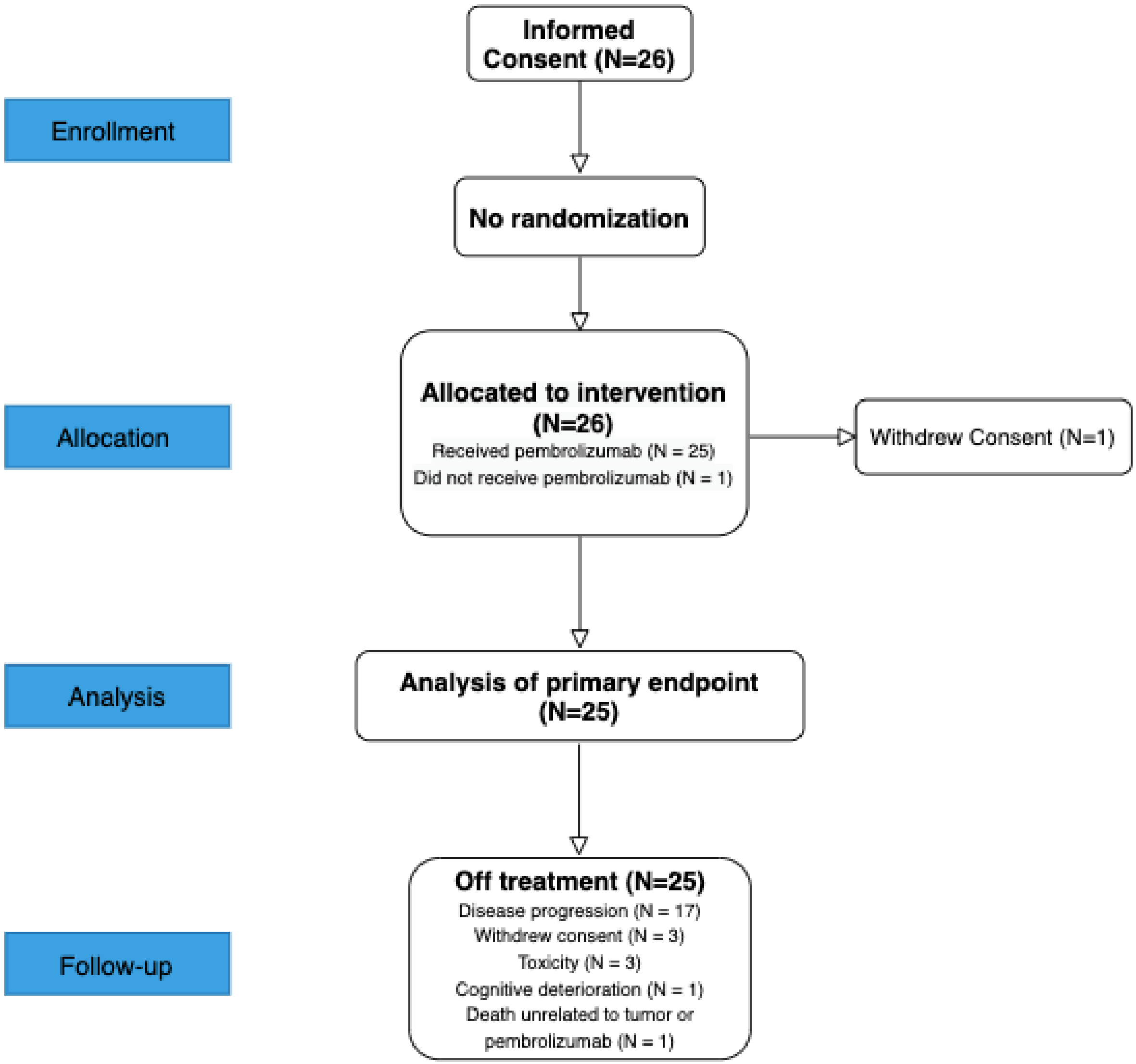

Supplemental Figure 2: Kaplan-Meier Curve for Overall Survival

Kaplan-Meier estimates of OS are shown. There were 15 deaths among 25 patients. Median survival was 20.2 months (90% CI: 14.8 – 25.8).

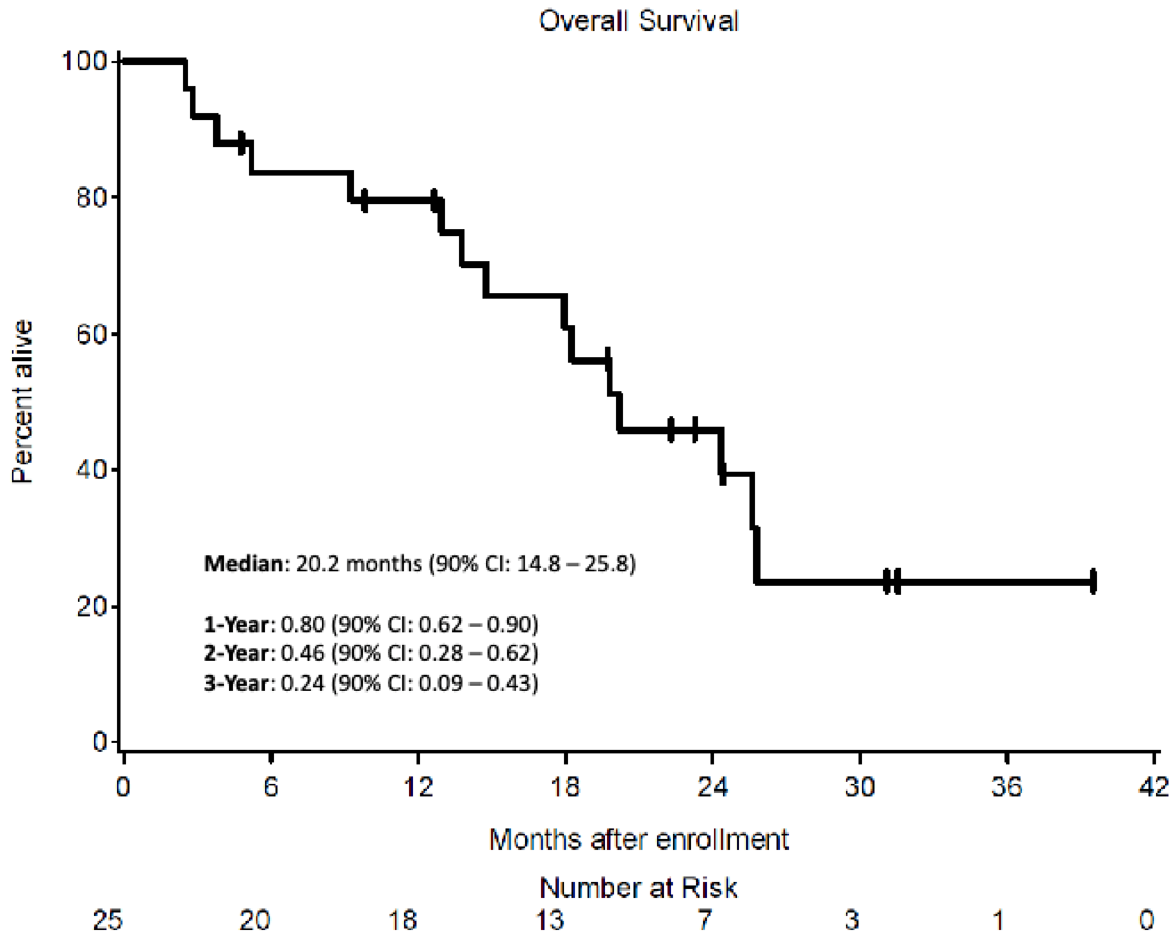

Supplemental Figure 3: Pre-treatment and Post-treatment Growth Rate & Tumor Size of Patients Enrolled due to Progressive Disease

Pre- and post-pembrolizumab growth rates for patients were calculated for the thirteen patients enrolled due to PD. Meningioma size was calculated using the sum LD of CNS target lesions from the T1 post-contrast MPRAGE sequence. A) The y-axis shows percent change in meningioma size relative to the baseline MRI, which was determined *a priori* by the treating oncologist at the beginning of the patient's course of therapy prior to pembrolizumab (e.g. systemic therapy or radiation). Pre-pembrolizumab (left of time 0 on x-axis) and post-pembrolizumab (right of time 0) tumor growth curves are illustrated for patients that were enrolled due to progressive disease. Six patients (e.g. MEN_03, 05, 07, 17, 21, and 24) displayed a stabilization in the tumor growth curve after starting pembrolizumab, compared to their pre-treatment growth arc. Two patients (MEN_03 and 21) had a decrease in tumor size (that did not meet criteria for partial response) after starting pembrolizumab. B) The y-axis shows tumor size relative to the baseline MRI. Here, four patients (MEN_03, 07, 21, 24) displayed stabilization in tumor growth, compared to the pre-treatment trajectory. One patient was excluded from this analysis as he was determined to have progressive disease on the basis of new foci of pachymeningeal enhancement.

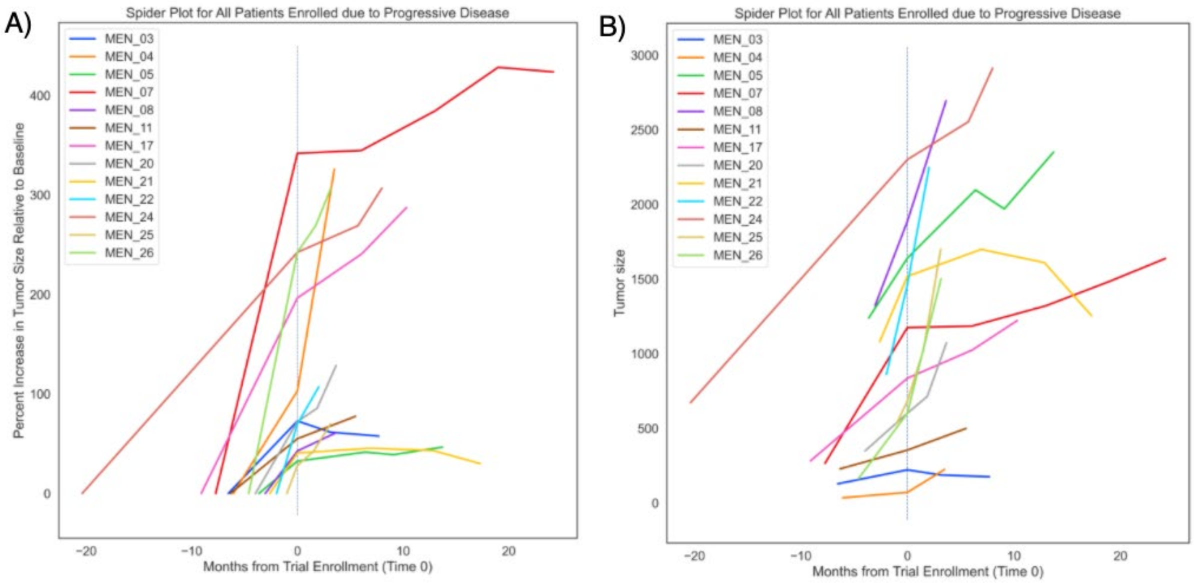
A)
Spider Plot for All Patients Enrolled due to Progressive Disease
MEN_03
MEN_04
MEN_05
MEN_07
MEN_08
MEN_11
MEN_17
MEN_20
MEN_21
MEN_22
MEN_24
MEN_25
MEN_26
Percent Increase in Tumor Size Relative to Baseline
Months from Trial Enrollment (Time 0)
B)
Spider Plot for All Patients Enrolled due to Progressive Disease
Tumor size
Months from Trial Enrollment (Time 0)

Supplemental Figure 4: Assessment of PD-L1 expression. Representative images of a meningioma with low PD-L1 expression (A. 100x, B. 400x; MPS = 5 and H score = 5; sample Y12) and a meningioma with high PD-L1 expression (C. 100x, D. 400x; MPS = 25 and H score = 30;sample Y25). PD-L1 IHC performed with monoclonal mouse anti–PD-L1 antibody clone 22C3.

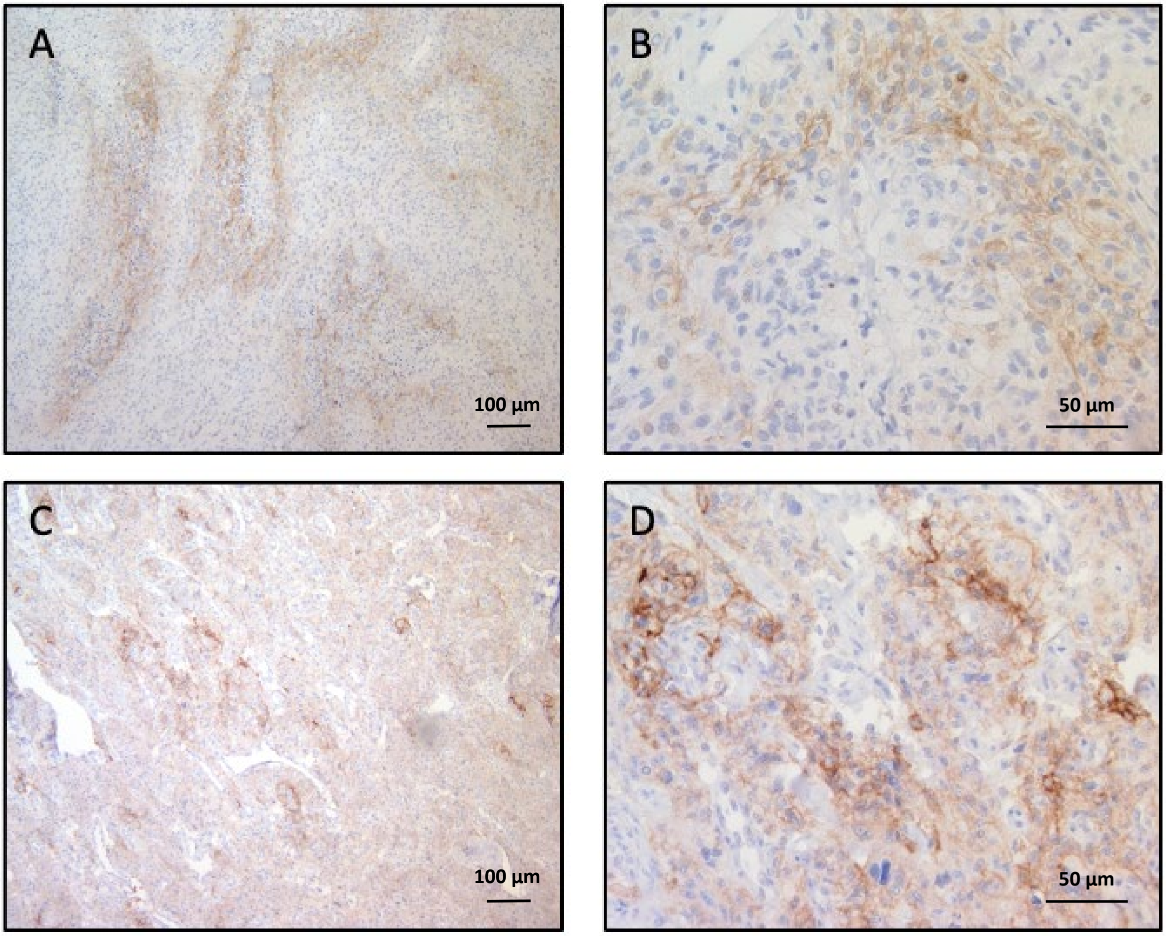

Supplemental Figure 5: Assessment of variability for pre-treatment ADC voxels

There was available ADC imaging data for seven patients who achieved PFS-6 and seven patients who did not. A coefficient of variation (CV) was calculated for each patient's meningioma. A boxplot of the CV values for each patient according to PFS (N: did not reach PFS-6; Y: did reach PFS-6). The individual data values are shown using circles. The diamond indicates the average. For the blue boxes, the top and bottom boundaries represent the 75th and 25th percentiles, respectively, and the center horizontal line is the median. The whiskers extend to the maximum and minimum of the distribution of values.

ID	*CV*	*PFS6*
4	29.9	NO
8	36.0	NO
11	38.0	NO
12	29.5	NO
20	36.8	NO
22	36.0	NO
23	42.9	NO
1	39.0	YES
5	25.6	YES
15	37.4	YES
16	38.0	YES
17	28.3	YES
21	21.1	YES
24	30.9	YES

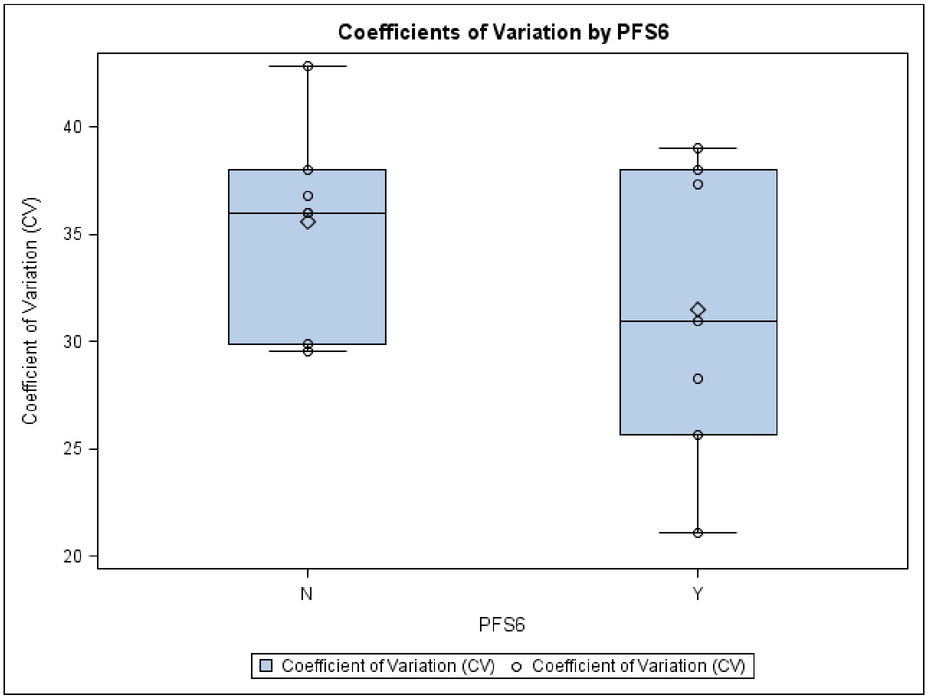
Coefficients of Variation by PFS6
Coefficient of Variation (CV)
40
35
30
25
20
N
Y
PFS6
Coefficient of Variation (CV)
Coefficient of Variation (CV)

Supplemental Table 1: Patient Demographics at Enrollment & Disease Characteristics

	N	%
**Gender**		
Female	11	42.3
Male	15	57.7
**Race**		
Black or African American	3	11.5
More Than One Race	1	3.8
Other	2	7.7
White	20	76.9
**ECOG Performance Status**		
0	15	57.7
1	10	38.5
2	1	3.9
**Median Age, years (range)**	61.4 (19-89)	
**Tumor Diagnosis**		
WHO Grade 2 Atypical Meningioma	23	88.5
WHO Grade 3 Anaplastic Meningioma	3	11.5
**Tumor Status at enrollment**		
Progressive Disease	15	57.7
Residual Measurable Disease	11	42.3
**Histologically proven meningioma with extracranial spread?**		
No	19	73.1
Yes	7	26.9
Time since initial diagnosis of high-grade meningioma, months (range)	11.5 (3-171)	
Prior Therapy		
Radiation	24	92.3
Median number of prior rounds of radiation (range)	2 (0-4)	
Local intracranial radiation	22	84.6
Whole brain radiation	1	3.8
Craniospinal radiation	1	3.8
Prior intracranial surgical resection	26	100
Median number of prior resections (range)	2.5 (1-5)	
Prior systemic therapy	10	38.4
*Chemotherapy**	3	11.5
Targeted therapy†	5	19.2

	N	%
Hormonal therapy‡	2	7.7

*****Prior meningioma-directed chemotherapy included carboplatin, etoposide, and temozolomide.
†Prior meningioma-directed targeted therapy included bevacizumab (2), vistusertib, and GSK2256098 (FAK inhibitor).
‡Prior meningioma-directed hormonal therapy included somatostatin analogs (2).

Supplemental Table 2: Patients Alive and Progression-Free at 6 Months (PFS-6)

Case number	***PFS months***	***Last known survival status***	***Progressed by the end of trial?***	***Tumor type***
1	12.9	Died	No	WHO Grade 2 Atypical Meningioma
2	8.3	Died	Yes	WHO Grade 2 Atypical Meningioma
3	19.8	Died	No	WHO Grade 2 Atypical Meningioma
5	14.8	Died	No	WHO Grade 2 Atypical Meningioma
7	23.7	Died	Yes	WHO Grade 2 Atypical Meningioma
9	9.7	Alive	Yes	WHO Grade 2 Atypical Meningioma
10	25.8	Died	No	WHO Grade 2 Atypical Meningioma
15	19.7	Alive	No	WHO Grade 2 Atypical Meningioma
16	24.3	Died	No	WHO Grade 2 Atypical Meningioma
17	11.3	Alive	Yes	WHO Grade 2 Atypical Meningioma
21	23.3	Alive	No	WHO Grade 2 Atypical Meningioma
24	7.6	Alive	Yes	WHO Grade 3 Atypical Meningioma

Supplemental Table 3: Adverse Events of All Attributions

		Toxicity Grade CTCAE v4.0			
		01-Mild	02-Moderate	03-Severe	04-Life Threatening
		N	N	N	N
**Toxicity Category CTCAE v4.0**	**Toxicity Description CTCAE v4.0**				
Blood and lymphatic system disorders	Anemia	5	2	-	-
	Decreased EGFR	-	1	-	-
	Elevated LDH	1	-	-	-
	Intermittent Decreased Red Blood Cell Count	1	-	-	-
	Leukocytosis	4	-	-	-
	Thrombotic thrombocytopenic purpura	1	-	-	-
Cardiac disorders	Restrictive cardiomyopathy	1	-	-	-
	Sinus bradycardia	1	-	-	-
	Sinus tachycardia	2	-	-	-
	Systolic Murmur	1	-	-	-
Ear and labyrinth disorders	Ear pain	1	1	-	-
	Hearing impaired	1	-	-	-
	Tinnitus	1	-	-	-
	Vertigo	1	-	-	-
Endocrine disorders	Adrenal insufficiency	1	-	-	-
	Hyperthyroidism	2	-	-	-
	Hypothyroidism	-	2	-	-
Eye disorders	Conjunctivitis	1	-	-	-
	Eye Discharge	1	-	-	-
	Eye pain	1	-	-	-
	Neurotropic Ulcer Left Eye	-	1	-	-
	Papilledema	1	-	-	-

		Toxicity Grade CTCAE v4.0			
		01-Mild	02-Moderate	03-Severe	04-Life Threatening
		N	N	N	N
Gastrointestinal disorders	Bloating	1	-	-	-
	Colitis	-	-	2	-
	Constipation	8	-	-	-
	Diarrhea	3	-	-	-
	Diverticulosis	1	-	-	-
	Esophagitis	1	-	-	-
	Gallstone	-	1	-	-
	Mouth Pain	1	-	-	-
	Nausea	2	-	1	-
	Oral pain	1	-	-	-
	Retroperitoneal hemorrhage	1	-	-	-
	Thrush	-	-	1	-
	Vomiting	3	-	1	-
General disorders and admin site conditions	Chills	2	-	-	-
	Edema face	2	-	-	-
	Edema limbs	5	-	-	-
	Fatigue	9	3	-	-
	Fever	2	-	-	-
	Flu like symptoms	1	-	-	-
	Gait disturbance	5	1	1	-
	Hypopituitarism	1	-	-	-
	Ingrown Toenail	1	-	-	-
	Left Arm Orbiting	1	-	-	-
	Localized edema	1	-	-	-
	Neck edema	1	-	-	-
	Non-cardiac chest pain	1	-	-	-
	Pain	4	-	-	-
	Postural Difficulty	-	1	-	-
	Right Forearm Lesion	1	-	-	-
Hepatobiliary disorders	Jaundice	1	-	-	-

		Toxicity Grade CTCAE v4.0			
		01-Mild	02-Moderate	03-Severe	04-Life Threatening
		N	N	N	N
Infections and infestations	Bilateral Toe Fungus	1	-	-	-
	Enterocolitis infectious	-	1	-	-
	Herpes Simplex Virus Vesicle	1	-	-	-
	Lung infection	1	1	-	-
	Sinusitis	1	-	-	-
	Skin infection	-	-	1	-
	Upper respiratory infection	1	-	-	-
	Urinary tract infection	-	1	-	-
	Vaginal infection	-	1	-	-
Injury, poisoning and procedural complications	Fall	7	2	1	-
	Fracture	1	-	-	-
	Spinal fracture	1	-	-	-
Investigations	Alanine aminotransferase increased	4	-	1	-
	Alkaline phosphatase increased	4	-	1	-
	Aspartate aminotransferase increased	3	-	-	1
	Blood bilirubin increased	-	1	-	-
	Blood corticotrophin decreased	1	-	-	-
	Cholesterol high	1	-	-	-
	Creatinine increased	1	-	-	-
	Decreased Free T4	1	-	-	-
	Elevated LDH	6	-	-	-
	Elevated Neutrophil Count	1	-	-	-
	Lipase increased	1	-	-	-
	Low TSH	1	-	-	-
	Lymphocyte count decreased	2	1	1	-
	Neutrophil count decreased	2	-	-	-
	Platelet count decreased	1	-	-	-
	Serum amylase increased	1	-	-	-
	Weight gain	1	-	-	-
	Weight loss	1	1	-	-
	White blood cell decreased	3	-	-	-

		Toxicity Grade CTCAE v4.0			
		01-Mild	02-Moderate	03-Severe	04-Life Threatening
		N	N	N	N
Metabolism and nutrition disorders	Anorexia	2	-	-	-
	Decreased TSH	1	-	-	-
	Dehydration	3	-	-	-
	Hyperglycemia	6	1	-	-
	Hyperkalemia	1	-	-	-
	Hyperuricemia	1	1	-	1
	Hypokalemia	1	-	-	-
	Hyponatremia	1	-	-	-
	Hypophosphatemia	3	4	-	-
Musculoskeletal and connective tissue disorders	Back pain	2	2	-	-
	Cramping, Left Leg	1	-	-	-
	Generalized muscle weakness	4	1	1	-
	Muscle weakness left-sided	-	3	-	-
	Muscle weakness lower limb	1	1	-	-
	Muscle weakness right-sided	1	1	-	-
	Muscle weakness upper limb	1	-	-	-
	Myalgia	2	-	-	-
	Neck pain	1	1	1	-
	Pain in extremity	-	1	-	-
	Right Ankle Pain	1	-	-	-
Neoplasms benign, malignant and unspecified (incl cysts and polyps)	Adrenal Adenoma	1	-	-	-

Supplemental Table 4: Adverse Events/Toxicities (Possibly, probably, or definitely related) Attributable to Pembrolizumab

		Toxicity Grade CTCAE v4.0			
		01-Mild	02-Moderate	03-Severe	04-Life Threatening
		N	N	N	N
**Toxicity Category CTCAE v4.0**	**Toxicity Description CTCAE v4.0**				
Blood and lymphatic system disorders	Anemia	4	1	-	-
	Elevated LDH	1	-	-	-
	Intermittent Decreased Red Blood Cell Count	1	-	-	-
Cardiac disorders	Sinus bradycardia	1	-	-	-
Endocrine disorders	Hyperthyroidism	2	-	-	-
	Hypothyroidism	-	2	-	-
Eye disorders	Conjunctivitis	1	-	-	-
	Eye Discharge	1	-	-	-
Gastrointestinal disorders	Colitis	-	-	1	-
	Constipation	4	-	-	-
	Diarrhea	3	-	-	-
	Nausea	2	-	1	-
	Oral pain	1	-	-	-
	Vomiting	2	-	1	-
General disorders and admin site conditions	Edema face	1	-	-	-
	Edema limbs	2	-	-	-
	Fatigue	8	2	-	-
	Fever	1	-	-	-
	Hypopituitarism	1	-	-	-
	Localized edema	1	-	-	-
	Pain	1	-	-	-
	Right Forearm Lesion	1	-	-	-
Hepatobiliary disorders	Jaundice	1	-	-	-
Infections and infestations	Skin infection	-	-	1	-
Injury, poisoning and procedural complications	Fall	1	-	-	-

		Toxicity Grade CTCAE v4.0			
		01-Mild	02-Moderate	03-Severe	04-Life Threatening
		N	N	N	N
Investigations	Alanine aminotransferase increased	4	-	1	-
	Alkaline phosphatase increased	3	-	1	-
	Aspartate aminotransferase increased	2	-	-	1
	Blood bilirubin increased	-	1	-	-
	Blood corticotrophin decreased	1	-	-	-
	Decreased Free T4	1	-	-	-
	Elevated LDH	3	-	-	-
	Lipase increased	1	-	-	-
	Low TSH	1	-	-	-
	Lymphocyte count decreased	1	1	-	-
	Neutrophil count decreased	2	-	-	-
	Serum amylase increased	1	-	-	-
	TSH Elevated	1	-	-	-
	Weight loss	1	1	-	-
	White blood cell decreased	3	-	-	-
Metabolism and nutrition disorders	Anorexia	1	-	-	-
	Decreased TSH	1	-	-	-
	Dehydration	1	-	-	-
	Hyperglycemia	5	-	-	-
	Hypophosphatemia	1	2	-	-
Musculoskeletal and connective tissue disorders	Back pain	1	-	-	-
	Generalized muscle weakness	1	-	-	-
	Myalgia	2	-	-	-
	Neck pain	1	-	-	-
Nervous system disorders	Dizziness	1	-	-	-
	Encephalopathy	-	-	1	-
	Headache	1	1	1	-
	Somnolence	1	-	-	-
Psychiatric disorders	Confusion	1	-	-	-
	Depression	1	-	-	-
	Insomnia	2	1	-	-
Respiratory, thoracic and mediastinal disorders	Cough	1	-	-	-
	Dyspnea	1	-	-	-
	Respiratory, thoracic and mediastinal disorders	-	1	-	-

		Toxicity Grade CTCAE v4.0			
		01-Mild	02-Moderate	03-Severe	04-Life Threatening
		N	N	N	N
Skin and subcutaneous tissue disorders	Alopecia	1	-	-	-
	Atypical Squamous Proliferation	1	-	-	-
	Dry skin	1	-	-	-
	Nail irritation	1	-	-	-
	Pruritus	4	2	-	-
	Rash maculo-papular	-	1	-	-
	Scaly Skin Irritation	1	-	-	-
	Truncal Rash	1	-	-	-
Vascular disorders	Hypertension	1	1	-	-

Supplemental Table 5: Comparisons of PD-L1 and TILs with PFS-6

P-values were calculated using Fisher's exact tests. We used multiple thresholds to define PD-L1 positivity. There was no statistically significant correlation between PD-L1 expression or TIL density and likelihood of achieving PFS-6.

	PFS6			
	No		Yes	
	N	%	N	%
PD-L1% > 0				
No	4	50.0	2	20.0
Yes	4	50.0	8	80.0
PD-L1 2+				
No	6	75.0	8	80.0
Yes	2	25.0	2	20.0
MPS Score > 1				
No	6	75.0	4	40.0
Yes	2	25.0	6	60.0
MPS Score ≥ 5				
No	7	87.5	6	60.0
Yes	1	12.5	4	40.0
TILs ≥ 3				
No	3	37.5	2	20.0
Yes	5	62.5	8	80.0

Fisher's Exact p-values

Comparison	P-value
PD-L1% > 0	0.32
PD-L1 2+	0.99
MPS Score > 1	0.19
MPS Score ≥ 5	0.31
TILs ≥ 3	0.61

Supplemental Table 6: Comparison of MPS and TILs on a Continuous Scale

P-values were calculated using exact Wilcoxon rank-sum tests. There was a weak trend towards higher MPS in patients who achieved PFS-6 (p = 0.13). There was no relationship between TIL density and PFS-6.

		N	Mean	Std	Min	Median	Max
MPS	PFS6						
	No	8	3.6	8.7	0.0	0.5	25.0
	Yes	10	6.3	9.5	0.0	2.5	30.0
TIL	PFS6						
	No	8	2.4	0.9	1.0	3.0	3.0
	Yes	10	2.8	0.4	2.0	3.0	3.0

Exact Wilcoxon rank-sum p-values: MPS, p=0.13; TIL, p=0.31

Supplementary Note

DF/HCC Protocol #: 17-296

TITLE: Phase II Trial of Pembrolizumab in Recurrent or Residual High Grade Meningioma

Coordinating Center: Massachusetts General Hospital

***Principal Investigators (PI):**
Priscilla Brastianos, MD
Massachusetts General Hospital
55 Fruit Street, Yawkey 9E
Boston, MA
pbrastianos@partners.org

Sandro Santagata, MD, PhD
Brigham and Women's Hospital
Dana Farber Cancer Institute
77 Avenue Louis Pasteur
Boston, MA
ssantagata@partners.org

Other Investigators:

Dan Cahill, MD PhD
Massachusetts General Hospital
Boston, MA

William Curry, MD
Massachusetts General Hospital
Boston, MA

Frederick Barker, MD
Massachusetts General Hospital
Boston, MA

Ian F. Dunn, MD
Dana-Farber Cancer Institute
Boston, MA

Kevin Oh, MD
Massachusetts General Hospital
Boston, MA

Elizabeth Gerstner, MD
Massachusetts General Hospital
Boston, MA

Statistician:

Anita Giobbie-Hurder
Department of Biostatistics
& Computational Biology
Dana-Farber Cancer Institute

Study Coordinator:

Edwin Nunez
Massachusetts General Hospital
Yawkey 9E, 55 Fruit Street
Telephone: 617-643-4395
Fax: 617-726-4319
enunez2@mgh.harvard.edu

Agent(s): Pembrolizumab

IND #: 133213
IND Sponsor: Priscilla K. Brastianos, MD

Protocol Type / Version # / Version Date: Revised / Version 4 / August 17, 2018

SCHEMA

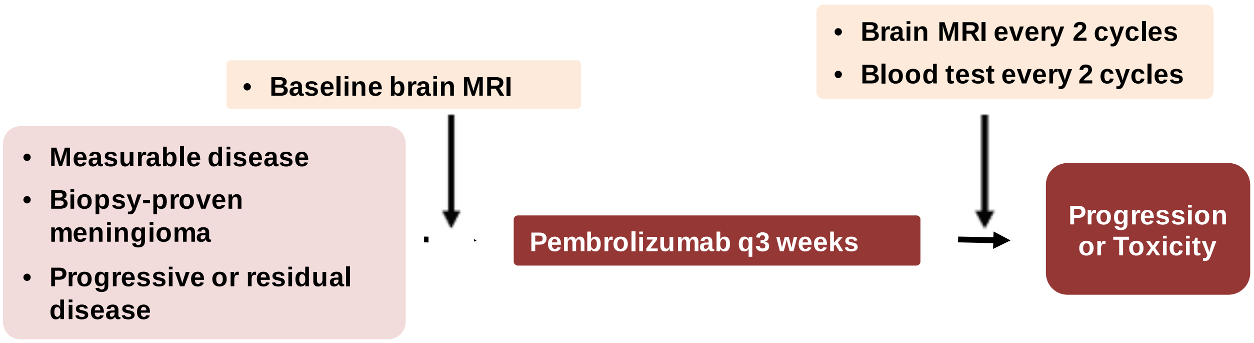
• Baseline brain MRI
• Brain MRI every 2 cycles
• Blood test every 2 cycles
• Measurable disease
• Biopsy-proven meningioma
• Progressive or residual disease
Pembrolizumab q3 weeks
Progression or Toxicity

TABLE OF CONTENTS

SCHEMA 3
1.1 Study Design 7
1.2 Primary Objective 7
1.3 Secondary Objectives 7
1.4 Exploratory Objectives 7

2. BACKGROUND 7
2.1 Meningioma 7
2.2 Pembrolizumab (MK-3475) 8
2.3 Rationale 10
2.4 Correlative Studies Background 11

3.0 PARTICIPANT SELECTION 11
3.3 Inclusion of Women and Minorities 15

4. REGISTRATION PROCEDURES 15
4.1 General Guidelines for DF/HCC Institutions 15
4.2 Registration Process for DF/HCC Institutions 15

5. TREATMENT PLAN 16
5.1 Treatment Regimen 16
5.2 Pre-Treatment Criteria 16
5.3 Agent Administration 16
5.4 General Concomitant Medication and Supportive Care Guidelines 17
5.5 Supportive Care Guidelines for Events of Clinical Interest and Immune Related Adverse Events (irAEs) 19
5.6 Acceptable Concomitant Medications 24
5.7 Prohibited Concomitant Medication 24
5.8 Diet 25
5.9 Contraception 25
5.10 Use in Pregnancy 26
5.11 Use in Nursing Women 26
5.12 Criteria for Taking a Participant Off Protocol Therapy 26
5.13 Duration of Follow Up 27
5.14 Criteria for Taking a Participant Off Study 27

6. DOSING DELAYS/DOSE MODIFICATIONS 28

7. ADVERSE EVENTS: LIST AND REPORTING REQUIREMENTS 29
7.1 Definition of an Overdose for This Protocol and Reporting of Overdose to the Overall PI and to Merck 30
7.2 Reporting of Pregnancy and Lactation to the Overall PI and to Merck 30
7.3 Immediate Reporting of Adverse Events to the Overall PI and to Merck 30

7.4 Events of Clinical Interest....31
7.5 Criteria for Taking a Participant Off Study....32
7.6 Overall PI Responsibility for Reporting Adverse Events....34
7.7 Adverse Event Characteristics....34
7.8 Expedited Adverse Event Reporting....35

8. PHARMACEUTICAL INFORMATION....36
8.1 Pembrolizumab....36
8.2 Description....36
8.3 Form....37
8.4 Packaging and Labeling Information....37
8.5 Clinical Supplies Disclosure....37
8.6 Storage, Handling and Preparation Requirements....37
8.7 Administration....38
8.8 Ordering....39
8.9 Accountability....39
8.10 Destruction and Return....39

9. BIOMARKER, CORRELATIVE, AND SPECIAL STUDIES....40
9.1 Exploratory Laboratory Correlative Studies....40
9.2 Background....40
9.3 Methodology....40
9.3.1 RNAscope assay and evaluation....40
9.3.2 Immunohistochemistry and evaluation....40
9.3.3 RNA-sequencing....41
9.3.4 Whole exome sequencing....41
9.3.5 Imaging Correlative Studies....41
9.4 Collection and Shipping of Tissue Specimen(s)....42

10. STUDY CALENDAR....43

11. MEASUREMENT OF EFFECT....46

12 End of Treatment/Intervention....57
12.1 Duration of Treatment....57
12.1.1 CR, PR, or SD....57
12.1.2 Disease Progression....57
12.2 Definitions and Follow-up Requirements....57
12.3 Extraordinary Medical Circumstances....58

13.0 Statistical Considerations....58

14 PUBLICATION PLAN....60

REFERENCES....61

APPENDIX A PERFORMANCE STATUS CRITERIA....64

OBJECTIVES

1.1 Study Design

This is a Phase 2 study of pembrolizumab in patients with recurrent or progressive high-grade meningioma.

1.2 Primary Objective

To estimate the progression-free survival at 6 months of pembrolizumab in recurrent high-grade meningioma (WHO grade II atypical meningioma and WHO grade III anaplastic meningioma)

1.3 Secondary Objectives

1. To determine the toxicity of pembrolizumab in patients with recurrent high-grade meningioma (WHO grade II atypical meningioma and WHO grade III anaplastic meningioma)
2. To estimate the overall survival of patients with recurrent high-grade meningioma receiving pembrolizumab
3. To estimate the response rate of patients with recurrent high grade meningioma receiving pembrolizumab

1.4 Exploratory Objectives

1. To determine molecular biomarkers of response using RNAscope in situ hybridization methodologies for immune checkpoint markers, immunohistochemistry to characterize the immune infiltrate and next generation DNA and RNA sequencing.
2. To determine if changes in brain MRI parameters correlate with tumor response.

2. BACKGROUND

2.1 Meningioma

Meningiomas are the most common primary brain tumor, with a prevalence of 170,000 cases in the US and an annual incidence of 18,000 new cases. Most meningiomas are of the typical variety – World Health Organization (WHO) grade I. However, depending on their location within the nervous system, grade I meningiomas can cause significant morbidity or mortality. Even after surgical resection, recurrence rates can be as high as 20%[1-3], and patients with grade I tumors have reduced long-term survival.

Approximately 20% of meningiomas are atypical (WHO grade II) and anaplastic (WHO grade III), defined by increased mitoses, necrosis, higher nuclear to cytoplasmic ratios, or histologic appearance resembling carcinoma, sarcoma, or melanoma[4]. Recurrence rates for WHO grade II and III meningiomas are 40% and 80%, respectively[3]. The prognosis of atypical and anaplastic meningiomas is poor, with 5-year overall survival rates between 47-65%[5,6].

Treatment options, particularly for the atypical or anaplastic meningiomas, are limited[5]. Radiation is frequently used as an adjunct to surgery; however, there are no effective chemotherapeutic options when surgery and radiation fail to offer durable long-term disease

control[7]. Traditional cytotoxic agents have minimal activity in this setting[8,9], and targeted agents in unselected patients[10] have demonstrated modest benefit at best. Response rate has been 0% in nearly all studies of systemic therapy in recurrent tumors of all grades[9,11-13]. A poor understanding of what drives meningioma development has hampered the development of therapeutic agents to supplement surgery and radiation. Therefore these patients have limited therapeutic options and effective treatments are greatly needed. While genomic studies have revealed mutations in oncogenes such as SMO and AKT in a small subset of meningioma[14,15], most tumors do not have mutations that can be targeted. Studies of high grade tumors have failed to reveal obvious drivers of malignant progression, rendering these tumors exceedingly challenging to treat.

2.2 Pembrolizumab (MK-3475)

The importance of intact immune surveillance in controlling outgrowth of neoplastic transformation has been known for decades. Accumulating evidence shows a correlation between tumor-infiltrating lymphocytes (TILs) in cancer tissue and favorable prognosis in various malignancies. In particular, the presence of CD8+ T-cells and the ratio of CD8+ effector T-cells / FoxP3+ regulatory T-cells seems to correlate with improved prognosis and long-term survival in many solid tumors.

The PD-1 receptor-ligand interaction is a major pathway hijacked by tumors to suppress immune control. The normal function of PD-1, expressed on the cell surface of activated T-cells under healthy conditions, is to down-modulate unwanted or excessive immune responses, including autoimmune reactions. PD-1 (encoded by the gene Pdcd1) is an Ig superfamily member related to CD28 and CTLA-4 which has been shown to negatively regulate antigen receptor signaling upon engagement of its ligands (PD-L1 and/or PD-L2). The structure of murine PD-1 has been resolved. PD-1 and family members are type I transmembrane glycoproteins containing an Ig Variable-type (V-type) domain responsible for ligand binding and a cytoplasmic tail which is responsible for the binding of signaling molecules. The cytoplasmic tail of PD-1 contains 2 tyrosine-based signaling motifs, an immunoreceptor tyrosine-based inhibition motif (ITIM) and an immunoreceptor tyrosine-based switch motif (ITSM). Following T-cell stimulation, PD-1 recruits the tyrosine phosphatases SHP-1 and SHP-2 to the ITSM motif within its cytoplasmic tail, leading to the dephosphorylation of effector molecules such as CD3ζ, PKCθ and ZAP70 which are involved in the CD3 T-cell signaling cascade.

The mechanism by which PD-1 down modulates T-cell responses is similar to, but distinct from that of CTLA-4 as both molecules regulate an overlapping set of signaling proteins. PD-1 was shown to be expressed on activated lymphocytes including peripheral CD4+ and CD8+ T-cells, B-cells, T regs and Natural Killer cells. Expression has also been shown during thymic development on CD4-CD8- (double negative) T-cells as well as subsets of macrophages and dendritic cells. The ligands for PD-1 (PD-L1 and PD-L2) are constitutively expressed or can be induced in a variety of cell types, including non-hematopoietic tissues as well as in various tumors. Both ligands are type I transmembrane receptors containing both IgV- and IgC-like domains in the extracellular region and contain short cytoplasmic regions with no known signaling motifs. Binding of either PD-1 ligand to PD-1 inhibits T-cell activation triggered through the T-cell receptor.

PD-L1 is expressed at low levels on various non-hematopoietic tissues, most notably on vascular endothelium, whereas PD-L2 protein is only detectably expressed on antigen-presenting cells found in lymphoid tissue or chronic inflammatory environments. PD-L2 is thought to control immune T-cell activation in lymphoid organs, whereas PD-L1 serves to dampen unwarranted T-cell function in peripheral tissues. Although healthy organs express little (if any) PD-L1, a variety of cancers were demonstrated to express abundant levels of this T-cell inhibitor. PD-1 has been suggested to regulate tumor-specific T-cell expansion in subjects with melanoma (MEL). This suggests that the PD-1/PD-L1 pathway plays a critical role in tumor immune evasion and should be considered as an attractive target for therapeutic intervention.

Pembrolizumab is a potent and highly selective humanized monoclonal antibody (mAb) of the IgG4/kappa isotype designed to directly block the interaction between PD-1 and its ligands, PD-L1 and PD-L2. Keytruda™ (pembrolizumab) has recently been approved in the United Stated for the treatment of patients with unresectable or metastatic melanoma and disease progression following ipilumumab and, if BRAF V600 mutation positive, a BRAF inhibitor.

An open-label Phase I trial (Protocol 001) is being conducted to evaluate the safety and clinical activity of single agent MK-3475. The dose escalation portion of this trial evaluated three dose levels, 1 mg/kg, 3 mg/kg, and 10 mg/kg, administered every 2 weeks (Q2W) in subjects with advanced solid tumors. All three dose levels were well tolerated and no dose-limiting toxicities were observed. This first in human study of MK-3475 showed evidence of target engagement and objective evidence of tumor size reduction at all dose levels (1 mg/kg, 3 mg/kg and 10 mg/kg Q2W). No MTD has been identified to date. 200mg IV Q3W will be the dose and schedule utilized in this protocol to test for initial tumor activity. Recent data from other clinical studies within the MK-3475 program has shown that a lower dose of MK-3475 and a less frequent schedule may be sufficient for target engagement and clinical activity.

Analysis of pharmacokinetic data for MK-3475 administered Q2W and Q3W showed slow systemic clearance, limited volume of distribution, and a long half-life. Pharmacodynamic data (IL-2 release assay) suggested that peripheral target engagement is durable (>21 days). This PK and pharmacodynamic data provides scientific rationale for using a Q3W dosing schedule.

A population pharmacokinetic analysis has been performed using serum concentration time data from 476 patients. Within the resulting population PK model, clearance and volume parameters of MK-3475 were found to be dependent on body weight. The relationship between clearance and body weight, with an allometric exponent of 0.59, is within the range observed for other antibodies and would support both body weight normalized dosing or a fixed dose across all body weights. MK-3475 has been found to have a wide therapeutic range based on the melanoma indication. The differences in exposure for a 200 mg fixed dose regimen relative to a 2 mg/kg Q3W body weight based regimen are anticipated to remain well within the established exposure margins of 0.5 – 5.0 for MK-3475 in the melanoma indication. The exposure margins are based on the notion of similar efficacy and safety in melanoma at 10 mg/kg Q3W vs. the proposed dose regimen of 2 mg/kg Q3W (i.e. 5-fold higher dose and exposure). The population PK evaluation revealed that there was no significant impact of tumor burden on exposure. In addition, exposure was similar between the NSCLC and melanoma indications. Therefore, there are no anticipated changes in exposure between different indication settings.

The rationale for further exploration of 2 mg/kg and comparable doses of pembrolizumab in solid tumors is based on: 1) similar efficacy and safety of pembrolizumab when dosed at either 2 mg/kg or 10 mg/kg Q3W in melanoma patients, 2) the flat exposure-response relationships of pembrolizumab for both efficacy and safety in the dose ranges of 2 mg/kg Q3W to 10 mg/kg Q3W, 3) the lack of effect of tumor burden or indication on distribution behavior of pembrolizumab (as assessed by the population PK model) and 4) the assumption that the dynamics of pembrolizumab target engagement will not vary meaningfully with tumor type.

The choice of the 200 mg Q3W as an appropriate dose for the switch to fixed dosing is based on simulations performed using the population PK model of pembrolizumab showing that the fixed dose of 200 mg every 3 weeks will provide exposures that 1) are optimally consistent with those obtained with the 2 mg/kg dose every 3 weeks, 2) will maintain individual patient exposures in the exposure range established in melanoma as associated with maximal efficacy response and 3) will maintain individual patients exposure in the exposure range established in melanoma that are well tolerated and safe.

A fixed dose regimen will simplify the dosing regimen to be more convenient for physicians and to reduce potential for dosing errors. A fixed dosing scheme will also reduce complexity in the logistical chain at treatment facilities and reduce wastage.

2.3 Rationale

Recently, great strides in immunotherapies have been made in the management of a number of malignancies namely the development of the immune checkpoint inhibitors targeting cytotoxic T-lymphocyte antigen 4 (CTLA4) and the programmed cell death (PD1) receptor-ligand (PDL1, PDL1). However, the role of these inhibitors in meningioma has not been explored. Preclinical data demonstrates an increase in PD-L1 expression as meningioma tumor grade increases and a decrease in the number of T-cells in higher grade meningioma [16]. Because there are no effective medical treatments for recurrent WHO grade II atypical and WHO grade III anaplastic meningioma[17] we characterized the immune infiltrate of meningioma and investigated the expression of molecules that might render these tumors susceptible to checkpoint blockade[16]. We characterized the immune infiltrate of 291 meningiomas including WHO grade I-III meningiomas using immunohistochemistry and we examined the expression of PD-L1 mRNA by RNAscope in situ hybridization and PD-L1 protein by immunohistochemistry and flow cytometry. In meningioma, the tumor infiltrating lymphocytes are predominantly T cells. In atypical and anaplastic meningioma, there is a sharp decrease in the number of T cells, including the numbers of CD4+ and CD8+ T cells and there is also an increase in the number of FOXP3 expressing immunoregulatory (Treg) cells. PD-L1 mRNA and protein expression is increased in atypical and anaplastic meningioma. Using patient derived meningioma cells, we confirmed that PD-L1 is expressed in meningioma cells themselves, and not solely in infiltrating immune cells. This work indicates that high-grade meningioma harbor an immunosuppressive tumor microenviroment and that increased Treg cells and elevated PD-L1 may contribute to the aggressive phenotype of these tumors.

We now hypothesize that patients with meningioma expressing PD-L1 will respond to

checkpoint blockade with pembrolizumab. We are thus proposing a prospective Phase 2 study of the PD-1 inhibitor pembrolizumab in patients with recurrent high grade (WHO grade II and III) using a dose schedule that has demonstrated efficacy in systemic tumors[18]. We propose to investigate the activity of pembrolizumab in a single cohort. We propose to incorporate monitoring with MR imaging, and pathology correlates from tissue resections (IHC markers for characterizing the immune infiltrate including PD-1 expressing cells and PD-L1 expression of the tumors via IHC and mRNA in situ hybridization/quantitative RNAscope), and next generation DNA sequencing and RNA-sequencing to identify genetic and imaging predictors of response.

2.4 Correlative Studies Background

2.4.1 Molecular Correlatives

Our objectives are to use next generation sequencing technologies to identify genetic predictors of response to immune checkpoint inhibitor blockade using clinical samples collected as part of clinical care. Our group has extensive experience in genomic characterization of a number of brain tumors including craniopharyngioma, brain metastases and their matched peripheral primaries and meningioma [14,19,20]. We will use whole exome or targeted exome sequencing panels that cover genes implicated in meningioma development and progression to identify markers of response. We will characterize mutations, gene rearrangements and copy number alterations from this data. RNA-sequencing will also be used to identify signatures of response. We will also characterize and quantify the levels of PD-1 and PD-L1 mRNA expression in the tumor samples using an in situ hybridization technology (RNAscope) as well as to characterize the immune infiltrate using immunohistochemistry markers and image quantification. Our goal is to use these techniques to identify genetic, mRNA and proteomic predictors of response to checkpoint inhibitors.

2.4.2 Imaging Correlatives

MRI is a very useful tool to non-invasively probe tumor response to treatment and is already part of the routine management of patients with meningioma. Imaging correlatives will be performed as outlined in Section 9.3.5.

3.0 PARTICIPANT SELECTION

3.1 Eligibility Criteria

3.1.1 Documentation Of Disease

Histologic Documentation: Histologically proven recurrent or residual intracranial or metastatic meningioma or meningioma with extracranial spread

Progressive OR residual disease, as defined by the following:

Progressive disease, as defined as an increase in size of a measurable meningioma lesion on imaging by greater than 25% (bidirectional area). The change must occur between scans separated by no more than 24 months.

Residual measurable disease: For Grade II or III meningioma, residual measurable disease immediately after surgery without requirement for progression. Residual measurable disease will be defined by measurable lesions with clearly defined margins by MRI scans, with a minimum diameter of 10mm in one dimension.

Post radiation patients: Patients with measurable and progressive meningioma who have received radiation are potentially eligible, but need to show evidence of progressive disease in the radiated field after completion of radiation. ≥ 24 weeks must have elapsed from completion of radiation to registration. Patients that have progressive disease outside of the radiation field do not need to wait 24 weeks from completion of radiation.

3.1.2 **Measurable Disease:** Measurable disease is defined by a main lesion measurable on MRI or CT images (MRI preferred) with clearly defined margins and ≥ 10 mm in one dimension. Multifocal disease is allowed as long as one lesion meets criteria for measurable disease and progressive disease. For measurable disease, refer to Section 11.0

3.1.3 **Prior Treatment**

- Prior medical therapy is allowed but not required.
- Meningioma that have resulted from prior radiation therapy are allowed.
- No limit on number of prior therapies.
- No chemotherapy, other investigational agents within 14 days of study treatment.
- No other concurrent investigational agents or other meningioma-directed therapy (chemotherapy, radiation) while on study.
- For patients treated with external beam radiation, interstitial brachytherapy or radiosurgery, an interval ≥ 24 weeks must have elapsed from completion of XRT to registration.
- Stable dose of dexamethasone 2mg or less for at least 7 days prior to initiation of treatment.
- Recovered to CTCAE grade 1 or less toxicity from other agents with exception of alopecia and fatigue.
- No craniotomy within 21 days of registration.

- Grade I meningiomas will be allowed as long as they meet criteria for progression as defined by 3.1.1.

3.1.4 **Not pregnant and not nursing:**

A female of childbearing potential is a sexually mature female who: 1) has not undergone a hysterectomy or bilateral oophorectomy; or 2) has not been naturally postmenopausal for at least 12 consecutive months (i.e., has had menses at any time

in the preceding 12 consecutive months). Female subject of childbearing potential should have a negative urine or serum pregnancy within 72 hours prior to receiving the first dose of study medication. If the urine test is positive or cannot be confirmed as negative, a serum pregnancy test will be required. Female subjects of childbearing potential should be willing to use 2 methods of birth control or be surgically sterile, or abstain from heterosexual activity for the course of the study through 120 days after the last dose of study medication.

3.1.5 **Age ≥ 18 years**

3.1.6 **ECOG Performance Status ≤ 2**

3.1.7 **Patient history:** Patients with history of NF may have other stable CNS tumors (schwannoma, acoustic neuroma or ependymoma) if lesions have been stable for 6 months.

3.1.8 Metastatic meningiomas (as defined by extracranial meningiomas) and meningioma with extra-cranial spread are allowed.

3.1.9 **Required Initial Laboratory Values**

Participants must have normal organ and marrow function as defined in Table 1, all screening labs should be performed within 14 days of treatment initiation.

Table 1: Required Initial Laboratory Values	
System	Laboratory Value
**Hematological**	
Absolute neutrophil count (ANC)	≥1,500 /mcL
Platelets	≥100,000 / mcL
Hemoglobin	≥9 g/dL or ≥5.6 mmol/L without transfusion or EPO dependency (within 7 days of assessment)
**Renal**	
Serum creatinine **OR**<br>Measured or calculated[a] creatinine clearance<br>(GFR can also be used in place of creatinine or CrCl)	≤1.5 X upper limit of normal (ULN) **OR**<br><br>≥60 mL/min for subject with creatinine levels > 1.5 X institutional ULN
**Hepatic**	
Serum total bilirubin	≤ 1.5 X ULN **OR**
	Direct bilirubin ≤ ULN for subjects with total bilirubin levels > 1.5 ULN
AST (SGOT) and ALT (SGPT)	≤ 2.5 X ULN
Albumin	≥2.5 g/dL
**Coagulation**	
International Normalized Ratio (INR) or Prothrombin Time	≤1.5 X ULN unless subject is receiving anticoagulant therapy

(PT)	as long as PT or PTT is within therapeutic range of intended use of anticoagulants
Activated Partial Thromboplastin Time (aPTT)	≤1.5 X ULN unless subject is receiving anticoagulant therapy<br>as long as PT or PTT is within therapeutic range of intended use of anticoagulants
[a]Creatinine clearance should be calculated per institutional standard.	

3.1.10 Male subjects should agree to use an adequate method of contraception starting with the first dose of study therapy through 120 days after the last dose of study therapy.

3.1.11 Ability to understand and the willingness to sign a written informed consent document.

3.2 Exclusion Criteria

3.2.1 Participants who have had chemotherapy, targeted small molecule therapy or study therapy within 14 days of protocol treatment, or those who have not recovered (i.e., ≤ Grade 1 or at baseline) from adverse events due to agents administered more than 2 weeks earlier. Subjects with ≤ Grade 2 neuropathy are an exception to this criterion and may qualify for the study. If subject received major surgery, they must have recovered adequately from the toxicity and/or complications from the intervention prior to starting therapy.

3.2.2 Participants with brainstem lesions

3.2.3 Participants who are receiving any other investigational agents.

3.2.4 Participants who have a diagnosis of an immunodeficiency.

3.2.5 Requires treatment with high dose systemic corticosteroids defined as dexamethasone >2mg/day or bioequivalent within at least 7 days of initiating therapy.

3.2.6 Has received systemic immunosuppressive treatments, aside from systemic corticosteroids as described in Section 5.7, within three months of start of study drug

3.2.7 Has a known hypersensitivity to pembrolizumab or any of its excipients

3.2.8 Has a known history of active TB (Bacillus Tuberculosis)

3.2.9 Uncontrolled intercurrent illness including, but not limited to, ongoing or active infection, symptomatic congestive heart failure, unstable angina pectoris, cardiac arrhythmia, or psychiatric illness/social situations that would limit compliance with study requirements.

3.2.10 Has known psychiatric or substance abuse disorders that would interfere with cooperation with the requirements of the trial.

3.2.11 Is pregnant or breastfeeding, or expecting to conceive or father children within the projected duration of the trial, starting with the pre-screening or screening visit through 120 days after the last dose of trial treatment.

3.2.12 Has a known additional malignancy that is progressing or requires active treatment. Exceptions include basal cell carcinoma of the skin or squamous cell carcinoma of the skin that has undergone potentially curative therapy or in situ cervical cancer or stable NF-related neoplasms (Section 3.1.7).

3.2.13 Has active autoimmune disease that has required systemic treatment in the past 2

years (i.e. with use of disease modifying agents, corticosteroids or immunosuppressive drugs). Replacement therapy (eg., thyroxine, insulin, or physiologic corticosteroid replacement therapy for adrenal or pituitary insufficiency, etc.) is not considered a form of systemic treatment.

3.2.14 Has known history of, or any evidence of active, non-infectious pneumonitis.

3.2.15 Has a known active infection requiring systemic therapy.

3.2.16 Has received prior therapy with an anti-PD-1, anti-PD-L1, or anti-PD-L2 agent.

3.2.17 Has a known history of Human Immunodeficiency Virus (HIV) (HIV 1/2 antibodies). HIV-positive participants on combination antiretroviral therapy are ineligible because of the potential for pharmacokinetic interactions with pembrolizumab. In addition, these participants are at increased risk of lethal infections when treated with marrow-suppressive therapy. Appropriate studies will be undertaken in participants receiving combination antiretroviral therapy when indicated.

3.2.18 Has known active Hepatitis B (e.g., HBsAg reactive) or Hepatitis C (e.g., HCV RNA [qualitative] is detected).

3.2.19 Has received a live vaccine within 30 days of planned start of study therapy. Note: Seasonal influenza vaccines for injection are generally inactivated flu vaccines and are allowed; however intranasal influenza vaccines (e.g., Flu-Mist®) are live attenuated vaccines, and are not allowed.

3.2.20 Unable to undergo brain MRI.

3.3 Inclusion of Women and Minorities

Both men and women of all races and ethnic groups are eligible for this trial.

4. REGISTRATION PROCEDURES

4.1 General Guidelines for DF/HCC Institutions

Institutions will register eligible participants in the Clinical Trials Management System (CTMS) OnCore. Registrations must occur prior to the initiation of protocol therapy. Any participant not registered to the protocol before protocol therapy begins will be considered ineligible and registration will be denied.

An investigator will confirm eligibility criteria and a member of the study team will complete the protocol-specific eligibility checklist.

Following registration, participants may begin protocol therapy. Issues that would cause treatment delays should be discussed with the Overall Principal Investigator (PI). If a participant does not receive protocol therapy following registration, the participant's registration on the study must be canceled. Registration cancellations must be made in OnCore as soon as possible.

4.2 Registration Process for DF/HCC Institutions

DF/HCC Standard Operating Procedure for Human Subject Research Titled *Subject Protocol Registration* (SOP #: REGIST-101) must be followed.

5. TREATMENT PLAN

5.1 Treatment Regimen

Pembrolizumab will be administered every 3 weeks, with 21 consecutive days defined as a treatment cycle. Treatment will be administered on an outpatient basis. Reported adverse events and potential risks are described in Section 7. Appropriate dose modifications are described in Section 6. No investigational or commercial agents or therapies other than those described below may be administered with the intent to treat the participant's malignancy.

The treatment to be used in this trial is outlined below in the table below.

Table 2: Dose of Pembrolizumab

Drug	Dose/Potency	Dose Frequency	Route of Administration	Regimen/Treatment Period	Cycle Length
Pembrolizumab	200 mg	Q3W	IV infusion	Day 1 of each 3 week cycle	3 weeks

5.2 Pre-Treatment Criteria

Informed consent will be obtained after the study has been fully explained to the subject and before the conduct of any screening procedures or assessments. If screening assessments occur within 7 days before start of study treatment, then they may serve as the baseline Cycle 1 Day 1 study tests and do not need to be repeated.

Pretreatment criteria will be assessed within 14 days of the first dose of study treatment to establish eligibility and baseline values. This will be considered the baseline clinical evaluation. Subsequent changes from screening physical exam (PE) findings that meet the definition of an AE will be recorded on the AE page of the eCRFs.

Demographic information and baseline characteristics will be collected at the Screening Visit. Standard demographic parameters include age, sex, and race/ethnicity (recorded in accordance with prevailing regulations). Baseline characteristics will include ECOG PS (Appendix A), disease status, medical histories, prior and concomitant medications, and PE findings.

5.3 Agent Administration

Trial treatment should be administered on Day 1 of each cycle after all procedures/assessments have been completed as detailed in the Study Calendar (Section 10). Trial treatment may be administered up to 3 days before or after the scheduled Day 1 of each cycle due to administrative reasons.

All trial treatments will be administered on an outpatient basis.

Pembrolizumab 200 mg will be administered as a 30 minute IV infusion every 3 weeks. Sites

should make every effort to target infusion timing to be as close to 30 minutes as possible.

However, given the variability of infusion pumps from site to site, a window of -5 minutes and +10 minutes is permitted (i.e., infusion time is 30 minutes: -5 min/+10 min).

The Pharmacy Manual contains specific instructions for the preparation of the pembrolizumab infusion fluid and administration of infusion solution.

5.4 General Concomitant Medication and Supportive Care Guidelines

Medications or vaccinations specifically prohibited in the exclusion criteria are not allowed during the ongoing trial. If there is a clinical indication for one of these or other medications or vaccinations specifically prohibited during the trial, discontinuation from trial therapy or vaccination may be required. The investigator should discuss any questions regarding this with the Merck Clinical team. The final decision on any supportive therapy or vaccination rests with the investigator and/or the subject's primary physician.

- **Diarrhea/Colitis**:
  Subjects should be carefully monitored for signs and symptoms of enterocolitis (such as diarrhea, abdominal pain, blood or mucus in stool, with or without fever) and of bowel perforation (such as peritoneal signs and ileus).

  - All subjects who experience diarrhea/colitis should be advised to drink liberal quantities of clear fluids. If sufficient oral fluid intake is not feasible, fluid and electrolytes should be substituted via IV infusion. For Grade 2 or higher diarrhea, consider GI consultation and endoscopy to confirm or rule out colitis.
  - For **Grade 2 diarrhea/colitis** that persists greater than 3 days, administer oral corticosteroids.
  - For **Grade 3 or 4 diarrhea/colitis** that persists > 1 week, treat with intravenous steroids followed by high dose oral steroids.
  - When symptoms improve to Grade 1 or less, steroid taper should be started and continued over no less than 4 weeks.
- **Nausea/vomiting:** Nausea and vomiting should be treated aggressively, and consideration should be given in subsequent cycles to the administration of prophylactic antiemetic therapy according to standard institutional practice. Subjects should be strongly encouraged to maintain liberal oral fluid intake.

- **Anti-infectives:** Subjects with a documented infectious complication should receive oral or IV antibiotics or other anti-infective agents as considered appropriate by the treating investigator for a given infectious condition, according to standard institutional practice.

- Immune-related adverse events: See Section 6.0 and Events of Clinical Interest Guidance Document for supportive care guidelines, including use of corticosteroids, regarding diagnosis and management of adverse experiences of a potential immunologic etiology.

  Management of Infusion Reactions: Signs and symptoms usually develop during or

shortly after drug infusion and generally resolve completely within 24 hours of completion of infusion.

Table 3 below shows treatment guidelines for subjects who experience an infusion reaction associated with administration of pembrolizumab (MK-3475).

Table 3 Infusion Reaction Treatment Guidelines

NCI CTCAE Grade	Treatment	Premedication at subsequent dosing
Grade 1<br>Mild reaction; infusion interruption not indicated; intervention not indicated	Increase monitoring of vital signs as medically indicated until the subject is deemed medically stable in the opinion of the investigator.	None
Grade 2<br>Requires infusion interruption but responds promptly to symptomatic treatment (e.g., antihistamines, NSAIDS, narcotics, IV fluids); prophylactic medications indicated for < =24 hrs	**Stop Infusion and monitor symptoms.**<br>Additional appropriate medical therapy may include but is not limited to:<br>IV fluids<br>Antihistamines<br>NSAIDS<br>Acetaminophen<br>Narcotics<br>Increase monitoring of vital signs as medically indicated until the subject is deemed medically stable in the opinion of the investigator.<br>If symptoms resolve within one hour of stopping drug infusion, the infusion may be restarted at 50% of the original infusion rate (e.g., from 100 mL/hr to 50 mL/hr). Otherwise dosing will be held until symptoms resolve and the subject should be premedicated for the next scheduled dose.<br>**Subjects who develop Grade 2 toxicity despite adequate premedication should be permanently discontinued from further trial treatment administration.**	Subject may be premedicated 1.5h (± 30 minutes) prior to infusion of pembrolizumab (MK-3475) with:<br><br>Diphenhydramine 50 mg po (or equivalent dose of antihistamine).<br><br>Acetaminophen 500-1000 mg po (or equivalent dose of antipyretic).
Grades 3 or 4<br>Grade 3:<br>Prolonged (i.e., not rapidly responsive to symptomatic medication and/or brief interruption of infusion); recurrence of symptoms	**Stop Infusion.**<br>Additional appropriate medical therapy may include but is not limited to:<br>IV fluids<br>Antihistamines<br>NSAIDS	No subsequent dosing

NCI CTCAE Grade	Treatment	Premedication at subsequent dosing
following initial improvement; hospitalization indicated for other clinical sequelae (e.g., renal impairment, pulmonary infiltrates)<br>Grade 4:<br>Life-threatening; pressor or ventilatory support indicated	Acetaminophen<br>Narcotics<br>Oxygen<br>Pressors<br>Corticosteroids<br>Epinephrine<br><br>Increase monitoring of vital signs as medically indicated until the subject is deemed medically stable in the opinion of the investigator.<br>Hospitalization may be indicated.<br>**Subject is permanently discontinued from further trial treatment administration.**	
Appropriate resuscitation equipment should be available in the room and a physician readily available during the period of drug administration.		

5.5 Supportive Care Guidelines for Immune Related Adverse Events (irAEs)

Adverse events (both non-serious and serious) associated with pembrolizumab exposure may represent an immunologic etiology. These adverse events may occur shortly after the first dose or several months after the last dose of treatment. Pembrolizumab must be withheld for drug-related toxicities and severe or life-threatening AEs as per the Table below. See Section 6.0 for supportive care guidelines, including use of corticosteroids.

Subjects should receive appropriate supportive care measures as deemed necessary by the treating investigator. Suggested supportive care measures for the management of adverse events with potential immunologic etiology are outlined below and in greater detail in the ECI guidance document. Where appropriate, these guidelines include the use of oral or intravenous treatment with corticosteroids as well as additional anti-inflammatory agents if symptoms do not improve with administration of corticosteroids. Note that several courses of steroid tapering may be necessary as symptoms may worsen when the steroid dose is decreased.

If an irAE is suspected, efforts should be made to rule out neoplastic, infectious, metabolic, toxin or other etiologic causes prior to labeling an adverse event as an irAE. Subjects who develop a Grade 2 or higher irAE should be discussed immediately with the overall Principal Investigator or designee. The treatment guidelines are intended to be applied when the investigator determines the events to be related to pembrolizumab.

It may be necessary to perform conditional procedures such as bronchoscopy, endoscopy, or skin photography as part of evaluation of the event. Suggested conditional procedures, as appropriate, can be found in the ECI guidance document.

Note: if after the evaluation the event is determined not to be related, the investigator does not need to follow the treatment guidance (as outlined below). Refer to Section 6.0 for dose modification.

- **Pneumonitis**:
    - For **Grade 2 events**, treat with systemic corticosteroids. When symptoms improve to Grade 1 or less, steroid taper should be started and continued over no less than 4 weeks.
    - For **Grade 3-4 events**, immediately treat with intravenous steroids. Administer additional anti-inflammatory measures, as needed.
    - Add prophylactic antibiotics for opportunistic infections in the case of prolonged steroid administration.

- **Type 1 diabetes mellitus (if new onset, including diabetic ketoacidosis [DKA]) or ≥ Grade 3 Hyperglycemia, if associated with ketosis (ketonuria) or metabolic acidosis (DKA)**

    - For **T1DM** or **Grade 3-4** Hyperglycemia
        - Insulin replacement therapy is recommended for Type I diabetes mellitus and for Grade 3-4 hyperglycemia associated with metabolic acidosis or ketonuria.
        - Evaluate patients with serum glucose and a metabolic panel, urine ketones, glycosylated hemoglobin, and C-peptide.

- **Hypophysitis**:

    - For **Grade 2** events, treat with corticosteroids. When symptoms improve to Grade 1 or less, steroid taper should be started and continued over no less than 4 weeks. Replacement of appropriate hormones may be required as the steroid dose is tapered.
    - For **Grade 3-4** events, treat with an initial dose of IV corticosteroids followed by oral corticosteroids. When symptoms improve to Grade 1 or less, steroid taper should be started and continued over no less than 4 weeks. Replacement of appropriate hormones may be required as the steroid dose is tapered.

- **Hyperthyroidism or Hypothyroidism**:
Thyroid disorders can occur at any time during treatment. Monitor patients for changes in thyroid function (at the start of treatment, periodically during treatment, and as indicated based on clinical evaluation) and for clinical signs and symptoms of thyroid disorders.

    - **Grade 2** hyperthyroidism events (and **Grade 2-4** hypothyroidism):

    - In hyperthyroidism, non-selective beta-blockers (e.g. propranolol) are suggested as initial therapy.
    - In hypothyroidism, thyroid hormone replacement therapy, with levothyroxine or liothyroinine, is indicated per standard of care.
  - **Grade 3-4** hyperthyroidism
    - Treat with an initial dose of IV corticosteroid followed by oral corticosteroids. When symptoms improve to Grade 1 or less, steroid taper should be started and continued over no less than 4 weeks. Replacement of appropriate hormones may be required as the steroid dose is tapered.

- **Hepatic**:
  - For **Grade 2** events, monitor liver function tests more frequently until returned to baseline values (consider weekly).
    - Treat with IV or oral corticosteroids
  - For **Grade 3-4** events, treat with intravenous corticosteroids for 24 to 48 hours.
  - When symptoms improve to Grade 1 or less, a steroid taper should be started and continued over no less than 4 weeks.

- **Renal Failure or Nephritis**:
  - For **Grade 2** events, treat with corticosteroids.
  - For **Grade 3-4** events, treat with systemic corticosteroids.
  - When symptoms improve to Grade 1 or less, steroid taper should be started and continued over no less than 4 weeks.

- **Cerebral Edema**
  Due to the immunologic nature of pembrolizumab administration, cerebral edema could theoretically result as a consequence of pembrolizumab administration due to immune infiltration of the brain. Symptoms related to cerebral edema may include headache or neurologic deficit that is either new or worsened. Patients with any signs or symptoms of cerebral edema should be treated as clinically appropriate including initiation or increased systemic corticosteroid dosing, treatment with an osmotic diuretic or surgical decompression. Subsequent pembrolizumab dosing should be immediately interrupted if significant clinical symptoms attributable to cerebral edema develop. Treatment with additional pembrolizumab doses may only be re-initiated if clinically significant symptoms attributable to cerebral edema have resolved to grade $\leq$ 1 or pre-treatment baseline. Patients who develop CTCAE 4.0 grade-4 cerebral edema attributable to pembrolizumab administration should not receive further pembrolizumab doses and should discontinue study therapy.

Recommendations to manage irAEs not detailed elsewhere in the protocol are detailed in Table 4.

Table 4 Dose Modification and Toxicity Management Guidelines for Immune-related AEs Associated with Pembrolizumab

General instructions:

1. Corticosteroid taper should be initiated upon AE improving to Grade 1 or less and continue to taper over at least 4 weeks.
2. For situations where pembrolizumab has been withheld, pembrolizumab can be resumed after AE has been reduced to Grade 1 or 0 and corticosteroid has been tapered. Pembrolizumab should be permanently discontinued if AE does not resolve within 12 weeks of last dose or corticosteroids cannot be reduced to ≤10 mg prednisone or equivalent per day within 12 weeks.
3. For severe and life-threatening irAEs, IV corticosteroid should be initiated first followed by oral steroid. Other immunosuppressive treatment should be initiated if irAEs cannot be controlled by corticosteroids.

**Immune-related AEs**	**Toxicity grade or conditions (CTCAEv 4.0)**	**Action taken to pembrolizumab**	**irAE management with corticosteroid and/or other therapies**	**Monitor and follow-up**
Pneumonitis	Grade 2	Withhold	• Administer corticosteroids (initial dose of 1-2 mg/kg prednisone or equivalent) followed by taper	• Monitor participants for signs and symptoms of pneumonitis<br>• Evaluate participants with suspected pneumonitis with radiographic imaging and initiate corticosteroid treatment<br>• Add prophylactic antibiotics for opportunistic infections
	Grade 3 or 4, or recurrent Grade 2	Permanently discontinue		
Diarrhea / Colitis	Grade 2 or 3	Withhold	• Administer corticosteroids (initial dose of 1-2 mg/kg prednisone or equivalent) followed by taper	• Monitor participants for signs and symptoms of enterocolitis (ie, diarrhea, abdominal pain, blood or mucus in stool with or without fever) and of bowel perforation (ie, peritoneal signs and ileus).<br>• Participants with ≥ Grade 2 diarrhea suspecting colitis should consider GI consultation and performing endoscopy to rule out colitis.<br>• Participants with diarrhea/colitis should be advised to drink liberal quantities of clear fluids. If sufficient oral fluid intake is not feasible, fluid and electrolytes should be substituted via IV infusion.
	Grade 4	Permanently discontinue		
AST / ALT elevation or Increased bilirubin	Grade 2	Withhold	• Administer corticosteroids (initial dose of 0.5- 1 mg/kg prednisone or equivalent) followed by taper	• Monitor with liver function tests (consider weekly or more frequently until liver enzyme value returned to baseline or is stable
	Grade 3 or 4	Permanently discontinue	• Administer corticosteroids (initial dose of 1-2 mg/kg prednisone or	

			equivalent) followed by taper	
Type 1 diabetes mellitus (T1DM) or Hyperglycemia	Newly onset T1DM or Grade 3 or 4 hyperglycemia associated with evidence of β-cell failure	Withhold	• Initiate insulin replacement therapy for participants with T1DM<br>• Administer anti-hyperglycemic in participants with hyperglycemia	• Monitor participants for hyperglycemia or other signs and symptoms of diabetes.
Hypophysitis	Grade 2	Withhold	• Administer corticosteroids and initiate hormonal replacements as clinically indicated.	• Monitor for signs and symptoms of hypophysitis (including hypopituitarism and adrenal insufficiency)
	Grade 3 or 4	Withhold or permanently discontinue[1]		
Hyperthyroidism	Grade 2	Continue	• Treat with non-selective beta-blockers (eg, propranolol) or thionamides as appropriate	• Monitor for signs and symptoms of thyroid disorders.
	Grade 3 or 4	Withhold or permanently discontinue[1]		
Hypothyroidism	Grade 2-4	Continue	• Initiate thyroid replacement hormones (eg, levothyroxine or liothyroinine) per standard of care	• Monitor for signs and symptoms of thyroid disorders.
Nephritis and Renal dysfunction	Grade 2	Withhold	• Administer corticosteroids (prednisone 1-2 mg/kg or equivalent) followed by taper.	• Monitor changes of renal function
	Grade 3 or 4	Permanently discontinue		
Myocarditis	Grade 1 or 2	Withhold	• Based on severity of AE administer corticosteroids	• Ensure adequate evaluation to confirm etiology and/or exclude other causes
	Grade 3 or 4	Permanently discontinue		
All other immune-related AEs	Intolerable/ persistent Grade 2	Withhold	• Based on type and severity of AE administer corticosteroids	• Ensure adequate evaluation to confirm etiology and/or exclude other causes
	Grade 3	Withhold or discontinue based on the type of event. Events that require discontinuation include and not limited to: Gullain-Barre		

		Syndrome, encephalitis		
	Grade 4 or recurrent Grade 3	Permanently discontinue		

1. Withhold or permanently discontinue pembrolizumab is at the discretion of the investigator or treating physician.

NOTE:
For participants with Grade 3 or 4 immune-related endocrinopathy where withhold of pembrolizumab is required, pembrolizumab may be resumed when AE resolves to ≤ Grade 2 and is controlled with hormonal replacement therapy or achieved metabolic control (in case of T1DM).

5.6 Acceptable Concomitant Medications

All treatments that the investigator considers necessary for a subject's welfare may be administered at the discretion of the investigator in keeping with the community standards of medical care. All concomitant medication will be recorded on the case report form (CRF) including all prescription, over-the-counter (OTC), herbal supplements, and IV medications and fluids. If changes occur during the trial period, documentation of drug dosage, frequency, route, and date may also be included on the CRF.

All concomitant medications received within 28 days before the first dose of trial treatment and 30 days after the last dose of trial treatment should be recorded. Concomitant medications administered after 30 days after the last dose of trial treatment should be recorded for SAEs and ECIs as defined in Section 7.4.

5.7 Prohibited Concomitant Medication

Subjects are prohibited from receiving the following therapies during the Screening and Treatment Phase (including retreatment for post-complete response relapse) of this trial:

- Antineoplastic systemic chemotherapy or biological therapy
- Immunotherapy not specified in this protocol
- Chemotherapy not specified in this protocol
- Investigational agents other than pembrolizumab
- Radiation therapy
    - Note: Radiation therapy to a symptomatic lesion may be allowed at the investigator's discretion. If radiation is given to a solitary lesion at the investigator's discretion, then the patient is considered to have progressive disease, but may continue on treatment if patient is clinically stable.
- Live vaccines within 30 days prior to the first dose of trial treatment and while participating in the trial. Examples of live vaccines include, but are not limited to, the following: intranasal influenza vaccines (e.g., Flu-Mist®), measles, mumps, rubella, varicella/zoster, yellow fever, rabies, BCG, and typhoid vaccine.

- Systemic glucocorticoids for any purpose other than to treat edema related to the meningioma or supportive post-operative management or to modulate symptoms from an event of clinical interest of suspected immunologic etiology. Intranasal, inhaled or topical corticosteroids (<5% of body surface area) for the treatment of mild/moderate asthma, allergies or dermatitis are permitted. The use of physiologic doses of corticosteroids may be approved after consultation with the Overall PI. Physiologic replacement of glucocorticoids as maintenance therapy for adrenal insufficiency are permitted. Standard doses of hydrocortisone for maintenance therapy are 10–20 mg/m2/day divided 2–4 times per day. For a subject with a body surface area (BSA) of 1.73 m2, this translates to a total dose of 34.6 mg of hydrocortisone per day. The equivalent dose of dexamethasone is 1.2 mg per day. Some subjects may additionally receive mineralocorticoid- replacement maintenance therapy with fludrocortisone. The maintenance dose of fludrocortisone for this indication is 0.05–0.1 mg/day. Antihistamines and other non- steroidal anti-allergy medications are also permitted.

Subjects who, in the assessment by the investigator, require the use of any of the aforementioned treatments for clinical management should be removed from the trial. Subjects may receive other medications that the investigator deems to be medically necessary.

The Exclusion Criteria describes other medications which are prohibited in this trial.

There are no prohibited therapies during the Post-Treatment Follow-up Phase.

5.8 Diet

Subjects should maintain a normal diet unless modifications are required to manage an adverse event such as diarrhea, nausea or vomiting.

5.9 Contraception

Pembrolizumab may have adverse effects on a fetus in utero. Furthermore, it is not known if pembrolizumab has transient adverse effects on the composition of sperm. Non-pregnant, non-breast-feeding women may be enrolled if they are willing to use 2 methods of birth control or are considered highly unlikely to conceive. Highly unlikely to conceive is defined as 1) surgically sterilized, or 2) postmenopausal (a woman who is ≥45 years of age and has not had menses for greater than 1 year will be considered postmenopausal), or 3) not heterosexually active for the duration of the study. The two birth control methods can be either two barrier methods or a barrier method plus a hormonal method to prevent pregnancy. Subjects should start using birth control from study Visit 1 throughout the study period up to 120 days after the last dose of study therapy.

The following are considered adequate barrier methods of contraception: diaphragm, condom (by the partner), copper intrauterine device, sponge, or spermicide. Appropriate hormonal contraceptives will include any registered and marketed contraceptive agent that contains an estrogen and/or a progestational agent (including oral, subcutaneous, intrauterine, or intramuscular agents).

Subjects should be informed that taking the study medication may involve unknown risks to the fetus (unborn baby) if pregnancy were to occur during the study. In order to participate in the study they must adhere to the contraception requirement (described above) for the duration of the study and during the follow-up period defined in section 7.2-Reporting of Pregnancy and Lactation to the Overall PI and to Merck. If there is any question that a subject will not reliably comply with the requirements for contraception, that subject should not be entered into the study.

5.10 Use in Pregnancy

If a subject inadvertently becomes pregnant while on treatment with pembrolizumab, the subject will immediately be removed from the study. The site will contact the subject at least monthly and document the subject's status until the pregnancy has been completed or terminated. The outcome of the pregnancy will be reported to the Overall PI and to Merck without delay and within 24 hours to the Overall PI and within 2 working days to Merck if the outcome is a serious adverse experience (e.g., death, abortion, congenital anomaly, or other disabling or life-threatening complication to the mother or newborn).

The study investigator will make every effort to obtain permission to follow the outcome of the pregnancy and report the condition of the fetus or newborn to the Overall PI. If a male subject impregnates his female partner the study personnel at the site must be informed immediately and the pregnancy reported to the Overall PI and to Merck and followed as described above and in Section 7.2.

5.11 Use in Nursing Women

It is unknown whether pembrolizumab is excreted in human milk. Since many drugs are excreted in human milk, and because of the potential for serious adverse reactions in the nursing infant, subjects who are breast-feeding are not eligible for enrollment.

5.12 Criteria for Taking a Participant Off Protocol Therapy

Duration of therapy will depend on individual response, evidence of disease progression and tolerance. In the absence of treatment delays due to adverse event(s), treatment may continue until one of the following criteria applies:

- Confirmed radiographic disease progression

  Note: For unconfirmed radiographic disease progression, please see Section 11.3.3

  Note: A subject may be granted an exception to continue on treatment with confirmed radiographic progression if clinically stable or clinically improved.

- Intercurrent illness that prevents further administration of treatment

- Unacceptable adverse event(s)

- Participant demonstrates an inability or unwillingness to comply with trial regimen and/or documentation requirements
- Participant decides to withdraw from the protocol therapy
- General or specific changes in the participant's condition render the participant unacceptable for further treatment in the judgment of the treating investigator
- The subject has a confirmed positive serum pregnancy test
- Subject is lost to follow-up
- Completed 24 months of uninterrupted treatment with pembrolizumab or 35 administrations of study medication, whichever is later.

Participants will be removed from the protocol therapy when any of these criteria apply. The reason for removal from protocol therapy, and the date the participant was removed, must be documented in the case report form (CRF). Alternative care options will be discussed with the participant.

An ODQ Treatment Ended/Off Study Form will be filled out when a participant is removed from protocol therapy. This form can be found on the DF/HCC website at http://www.dfhcc.harvard.edu/research/clinical-research-support/document-library-forms-sops-etc/.

In the event of unusual or life-threatening complications, treating investigators must immediately notify the Overall PI, Priscilla Brastianos at 617-643-1938.

5.13 Duration of Follow Up

After the end of treatment, each subject will be followed for 30 days for adverse event monitoring (serious adverse events will be collected for 90 days after the end of treatment as described in Section 7.3). Subjects who discontinue for reasons other than progressive disease will have post-treatment follow-up for disease status until disease progression, initiating a non-study cancer treatment, withdrawing consent or becoming lost to follow-up. After documented disease progression each subject will be followed by telephone for overall survival until death, or withdrawal of consent, whichever occurs first.

5.14 Criteria for Taking a Participant Off Study

Participants will be removed from study when any of the following criteria apply:

- Lost to follow-up
- Withdrawal of consent for data submission
- Death

The reason for taking a participant off study, and the date the participant was removed, must be documented in the case report form (CRF).

For Decentralized Subject Registrations, the research team updates the relevant Off Treatment/Off Study information in OnCore.

6. DOSING DELAYS/DOSE MODIFICATIONS

The descriptions and grading scales found in the revised NCI Common Terminology Criteria for Adverse Events (CTCAE) version 4.0 will be utilized for dose delays and dose modifications. A copy of the CTCAE version 4.0 can be downloaded from the CTEP website.
http://ctep.cancer.gov/protocolDevelopment/electronic_applications/ctc.htm.

Pembrolizumab will be withheld for drug-related Grade 4 hematologic toxicities, non-hematological toxicity ≥ Grade 3 including laboratory abnormalities despite appropriate replacement therapies when appropriate, and severe or life-threatening AEs as per Table 5.

Table 5 Dose modification guidelines for drug-related adverse events

Toxicity	Hold Treatment For Grade	Timing for Restarting Treatment	Treatment Discontinuation
Diarrhea/Colitis	2-3	Toxicity resolves to Grade 0-1	Toxicity does not resolve within 12 weeks of last dose or inability to reduce corticosteroid to 10 mg or less of prednisone or equivalent per day within 12 weeks
	4	Permanently discontinue	Permanently discontinue
AST, ALT, or Increased Bilirubin	2	Toxicity resolves to Grade 0-1	Toxicity does not resolve within 12 weeks of last dose
	3-4	Permanently discontinue (see exception below)[a]	Permanently discontinue
Type 1 diabetes mellitus (if new onset) or Hyperglycemia	T1DM or 3-4	Hold pembrolizumab for new onset Type 1 diabetes mellitus or Grade 3-4 hyperglycemia associated with evidence of beta cell failure	Resume pembrolizumab when patients are clinically and metabolically stable
Hypophysitis	2-4	Toxicity resolves to Grade 0-1. Therapy with pembrolizumab can be continued while endocrine replacement therapy is instituted	Toxicity does not resolve within 12 weeks of last dose or inability to reduce corticosteroid to 10 mg or less of prednisone or equivalent per day within 12 weeks
Hyperthyroidism	3	Toxicity resolves to Grade 0-1	Toxicity does not resolve within 12 weeks of last dose or inability to reduce corticosteroid to 10 mg or less of prednisone or equivalent per day within 12 weeks
	4	Permanently discontinue	Permanently discontinue
Hypothyroidism		Therapy with pembrolizumab can be continued while thyroid replacement therapy is instituted	Therapy with pembrolizumab can be continued while thyroid replacement therapy is instituted
Infusion Reaction	2[b]	Toxicity resolves to Grade 0-1	Permanently discontinue if toxicity develops despite adequate premedication
	3-4	Permanently discontinue	Permanently discontinue
Pneumonitis	2	Toxicity resolves to Grade 0-1	Toxicity does not resolve within 12 weeks of last dose or inability to reduce corticosteroid to 10 mg or less of prednisone or equivalent per day within 12 weeks

Toxicity	Hold Treatment For Grade	Timing for Restarting Treatment	Treatment Discontinuation
	3-4	Permanently discontinue	Permanently discontinue
Renal Failure or Nephritis	2	Toxicity resolves to Grade 0-1	Toxicity does not resolve within 12 weeks of last dose or inability to reduce corticosteroid to 10 mg or less of prednisone or equivalent per day within 12 weeks
	3-4	Permanently discontinue	Permanently discontinue
All Other Drug-Related Toxicity[c]	3 or Severe	Toxicity resolves to Grade 0-1	Toxicity does not resolve within 12 weeks of last dose or inability to reduce corticosteroid to 10 mg or less of prednisone or equivalent per day within 12 weeks
	4	Permanently discontinue	Permanently discontinue

Note: Permanently discontinue for any severe or Grade 3 drug-related AE that recurs or any life-threatening event.

[a] For patients with liver metastasis who begin treatment with Grade 2 AST or ALT, if AST or ALT increases by greater than or equal to 50% relative to baseline and lasts for at least 1 week then patients should be discontinued.

[b] If symptoms resolve within one hour of stopping drug infusion, the infusion may be restarted at 50% of the original infusion rate (e.g., from 100 mL/hr to 50 mL/hr). Otherwise dosing will be held until symptoms resolve and the subject should be premedicated for the next scheduled dose; Refer to

Table – Infusion Treatment Guidelines for further management details.

[c] Patients with intolerable or persistent Grade 2 drug-related AE may hold study medication at physician discretion. Permanently discontinue study drug for persistent Grade 2 adverse reactions for which treatment with study drug has been held, that do not recover to Grade 0-1 within 12 weeks of the last dose.

Dosing interruptions are permitted in the case of medical / surgical events or logistical reasons not related to study therapy (e.g., elective surgery, unrelated medical events, patient vacation, and/or holidays). Subjects should be placed back on study therapy within 3 weeks of the scheduled interruption, unless otherwise discussed with the PI of the study. The reason for interruption should be documented in the patient's study record.

7. ADVERSE EVENTS: LIST AND REPORTING REQUIREMENTS

An adverse event is defined as any untoward medical occurrence in a patient or clinical investigation subject administered a pharmaceutical product and which does not necessarily have to have a causal relationship with this treatment. An adverse event can therefore be any unfavorable and unintended sign (including an abnormal laboratory finding, for example), symptom, or disease temporally associated with the use of a medicinal product or protocol-specified procedure, whether or not considered related to the medicinal product or protocol-specified procedure. Any worsening (i.e., any clinically significant adverse change in frequency and/or intensity) of a preexisting condition that is temporally associated with the use of the Merck's product, is also an adverse event.

Changes resulting from normal growth and development that do not vary significantly in frequency or severity from expected levels are not to be considered adverse events. Examples of this may include, but are not limited to, teething, typical crying in infants and children and onset of menses or menopause occurring at a physiologically appropriate time. Merck product includes any pharmaceutical product, biological product, device, diagnostic agent or protocol-specified procedure, whether investigational (including placebo or active comparator medication) or marketed, manufactured by, licensed by, provided by or distributed by Merck for human use.

Adverse events may occur during the course of the use of Merck product in clinical trials or within the follow-up period specified by the protocol, or prescribed in clinical practice, from overdose (whether accidental or intentional), from abuse and from withdrawal.

Adverse events may also occur in screened subjects during any pre-allocation baseline period as a result of a protocol-specified intervention, including washout or discontinuation of usual therapy, diet, placebo treatment or a procedure. Progression of the cancer under study is not considered an adverse event unless it is considered to be drug related by the investigator.

All adverse events will be recorded from the time of patient registration through 30 days following cessation of treatment and at each examination on the Adverse Event case report forms/worksheets. The reporting timeframe for adverse events meeting any serious criteria is described in section 7.3.

7.1 Definition of an Overdose for This Protocol and Reporting of Overdose to the Overall PI and to Merck

For purposes of this trial, an overdose of pembrolizumab will be defined as any dose of 1,000 mg or greater (≥5 times the indicated dose). No specific information is available on the treatment of overdose of pembrolizumab. Appropriate supportive treatment should be provided if clinically indicated. In the event of overdose, the subject should be observed closely for signs of toxicity. Appropriate supportive treatment should be provided if clinically indicated. If an adverse event(s) is associated with ("results from") the overdose of a Merck product, the adverse event(s) is reported as a serious adverse event, even if no other seriousness criteria are met. If a dose of Merck's product meeting the protocol definition of overdose is taken without any associated clinical symptoms or abnormal laboratory results, the overdose is reported as a non-serious Event of Clinical Interest (ECI), using the terminology "accidental or intentional overdose without adverse effect."

All reports of overdose with and without an adverse event must be reported within 24 hours to the Overall PI and within 2 working days hours to Merck Global Safety. (Attn: Worldwide Product Safety; FAX 215 993-1220)

7.2 Reporting of Pregnancy and Lactation to the Overall PI and to Merck

Although pregnancy and lactation are not considered adverse events, it is the responsibility of investigators or their designees to report any pregnancy or lactation in a subject (spontaneously reported to them), including the pregnancy of a male subject's female partner that occurs during the trial or within 120 days of completing the trial completing the trial, or 30 days following cessation of treatment if the subject initiates new anticancer therapy, whichever is earlier. All subjects and female partners of male subjects who become pregnant must be followed to the completion/termination of the pregnancy. Pregnancy outcomes of spontaneous abortion, missed abortion, benign hydatidiform mole, blighted ovum, fetal death, intrauterine death, miscarriage and stillbirth must be reported as serious events (Important Medical Events). If the pregnancy continues to term, the outcome (health of infant) must also be reported.

Such events must be reported within 24 hours to the Overall PI and within 2 working days to Merck Global Safety. (Attn: Worldwide Product Safety; FAX 215 993-1220)

7.3 Immediate Reporting of Adverse Events to the Overall PI and to Merck

7.3.1 Serious Adverse Events

A serious adverse event is any adverse event occurring at any dose or during any use of Merck's product that:

- Results in death;
- Is life threatening;
- Results in persistent or significant disability/incapacity;
- Results in or prolongs an existing inpatient hospitalization;
- Is a congenital anomaly/birth defect;
- Is a new cancer (that is not a condition of the study);
- Is associated with an overdose;
- Is another important medical event

Refer to Table 6 below for additional details regarding each of the above criteria.

Any serious adverse event, or follow up to a serious adverse event, including death due to any cause other than progression of the cancer under study that occurs to any subject from the time of patient registration through 90 days following cessation of treatment, or the initiation of new anti-cancer therapy, whichever is earlier, whether or not related to Merck product, must be reported within 24 hours to the Overall PI and within 2 working days to Merck Global Safety.
Non-serious Events of Clinical Interest will be forwarded to Merck Global Safety and will be handled in the same manner as SAEs.

Additionally, any serious adverse event, considered by an investigator who is a qualified physician to be related to Merck product that is brought to the attention of the investigator at any time outside of the time period specified in the previous paragraph also must be reported immediately to the Overall PI and to Merck.

SAE reports and any other relevant safety information are to be forwarded to the Merck Global Safety facsimile number: +1-215-993-1220

A copy of all 15 Day Reports and Annual Progress Reports is submitted as required by FDA, European Union (EU), Pharmaceutical and Medical Devices agency (PMDA) or other local regulators. Investigators will cross reference this submission according to local regulations to the Merck Investigational Compound Number (IND, CSA, etc.) at the time of submission.

Additionally investigators will submit a copy of these reports to Merck & Co., Inc. (Attn: Worldwide Product Safety; FAX 215 993-1220) at the time of submission to FDA.

All subjects with serious adverse events must be followed up for outcome.

7.4 Events of Clinical Interest

Selected non-serious and serious adverse events are also known as Events of Clinical Interest (ECI) and must be reported within 24 hours to the Overall PI and within 2 working days to Merck Global Safety. (Attn: Worldwide Product Safety; FAX 215 993-1220). For the time period beginning when the consent form is signed until treatment allocation/randomization, any ECI, or follow up to an ECI, that occurs to any subject must be reported within 24 hours to the Overall PI and within 2 working days to Merck Global Safety if it causes the subject to be excluded from the trial, or is the result of a protocol-specified intervention, including but not limited to washout or discontinuation of usual therapy, diet, placebo treatment or a procedure.

For the time period beginning at patient registration through 90 days following cessation of treatment, or 30 days following cessation of treatment if the subject initiates new anticancer therapy, whichever is earlier, any ECI, or follow up to an ECI, whether or not related to Merck product, must be reported within 24 hours to the Overall PI and within 24 hours to Merck Global Safety.

Events of clinical interest for this trial include:

1. an overdose of Merck product, as defined in Section 7.1 - Definition of an Overdose for This Protocol and Reporting of Overdose to the Overall PI, that is not associated with clinical symptoms or abnormal laboratory results.
2. an elevated AST or ALT lab value that is greater than or equal to 3X the upper limit of normal and an elevated total bilirubin lab value that is greater than or equal to 2X the upper limit of normal and, at the same time, an alkaline phosphatase lab value that is less than 2X the upper limit of normal, as determined by way of protocol-specified laboratory testing or unscheduled laboratory testing.*

*Note: These criteria are based upon available regulatory guidance documents. The purpose of the criteria is to specify a threshold of abnormal hepatic tests that may require an additional evaluation for an underlying etiology.

7.5 Criteria for Taking a Participant Off Study

An investigator who is a qualified physician will evaluate all adverse events according to the NCI Common Terminology for Adverse Events (CTCAE), version 4.0. Any adverse event which changes CTCAE grade over the course of a given episode will have each change of grade recorded on the adverse event case report forms/worksheets. All adverse events regardless of CTCAE grade must also be evaluated for seriousness.

Table 6 Evaluating Adverse Events

An investigator who is a qualified physician, will evaluate all adverse events as to:

**V4.0 CTCAE Grading**	**Grade 1**	**Mild; asymptomatic or mid symptoms; clinical or diagnostic observations only; intervention not indicated.**
	**Grade 2**	**Moderate; minimal, local or noninvasive intervention indicated; limiting age-appropriate instrumental ADL.**
	**Grade 3**	**Severe or medically significant but not immediately life-threatening; hospitalization or prolongation or hospitalization indicated; disabling; limiting self-care ADL.**
	**Grade 4**	**Life threatening consequences; urgent intervention indicated.**
	**Grade 5**	**Death related to AE**
**Seriousness**	A serious adverse event is any adverse event occurring at any dose or during any use of Merck product that:	
	†**Results in death**; or	
	†**Is life threatening; or** places the subject, in the view of the investigator, at immediate risk of death from the event as it occurred (Note: This does not include an adverse event that, had it occurred in a more severe form, might have caused death.); or	
	†**Results in a persistent or significant disability/incapacity** (substantial disruption of one's ability to conduct normal life functions); or	
	†**Results in or prolongs an existing inpatient hospitalization** (hospitalization is defined as an inpatient admission, regardless of length of stay, even if the hospitalization is a precautionary measure for continued observation. (Note: Hospitalization [including hospitalization for an elective procedure] for a preexisting condition which has not worsened does not constitute a serious adverse event.); or	
	†**Is a congenital anomaly/birth defect** (in offspring of subject taking the product regardless of time to diagnosis);or	
	**Is a** new **cancer;** (that is not a condition of the study) **or**	
	**Is an overdose** (whether accidental or intentional). Any adverse event associated with an overdose is considered a serious adverse event. An overdose that is not associated with an adverse event is considered a non-serious event of clinical interest and must be reported within 24 hours.	
	**Other important medical events** that may not result in death, not be life threatening, or not require hospitalization may be considered a serious adverse event when, based upon appropriate medical judgment, the event may jeopardize the subject and may require medical or surgical intervention to prevent one of the outcomes listed previously (designated above by a †).	
**Duration**	Record the start and stop dates of the adverse event. If less than 1 day, indicate the appropriate length of time and units	
**Action taken**	Did the adverse event cause the Merck product to be discontinued?	
**Relationship to test drug**	Did the Merck product cause the adverse event? The determination of the likelihood that the Merck product caused the adverse event will be provided by an investigator who is a qualified physician. The investigator's signed/dated initials on the source document or worksheet that supports the causality noted on the AE form, ensures that a medically qualified assessment of causality was done. This initialed document must be retained for the required regulatory time frame. The criteria below are intended as reference guidelines to assist the investigator in assessing the likelihood of a relationship between the test drug and the adverse event based upon the available information. **The following components are to be used to assess the relationship between the Merck product and the AE**; the greater the correlation with the components and their respective elements (in number and/or intensity), the more likely the Merck product caused the adverse event (AE):	
	**Exposure**	Is there evidence that the subject was actually exposed to the Merck product such as: reliable history, acceptable compliance assessment (pill count, diary, etc.), expected pharmacologic effect, or measurement of drug/metabolite in bodily specimen?
	**Time Course**	Did the AE follow in a reasonable temporal sequence from administration of the Merck product? Is the time of onset of the AE compatible with a drug-induced effect (applies to trials with investigational medicinal product)?
	**Likely Cause**	Is the AE not reasonably explained by another etiology such as underlying disease, other drug(s)/vaccine(s), or other host or environmental factors

**Relationship to Merck product (continued)**	**The following components are to be used to assess the relationship between the test drug and the AE: (continued)**	
	**Dechallenge**	Was the Merck product discontinued or dose/exposure/frequency reduced?<br>If yes, did the AE resolve or improve?<br>If yes, this is a positive dechallenge. If no, this is a negative dechallenge.<br>(Note: This criterion is not applicable if: (1) the AE resulted in death or permanent disability; (2) the AE resolved/improved despite continuation of the Merck product; or (3) the trial is a single-dose drug trial); or (4) Merck product(s) is/are only used one time.)
	**Rechallenge**	Was the subject re-exposed to the Merck product in this study?<br>If yes, did the AE recur or worsen?<br>If yes, this is a positive rechallenge. If no, this is a negative rechallenge.<br>(Note: This criterion is not applicable if: (1) the initial AE resulted in death or permanent disability, or (2) the trial is a single-dose drug trial); or (3) Merck product(s) is/are used only one time).<br>NOTE: IF A RECHALLENGE IS PLANNED FOR AN ADVERSE EVENT WHICH WAS SERIOUS AND WHICH MAY HAVE BEEN CAUSED BY THE MERCK PRODUCT, OR IF REEXPOSURE TO THE MERCK PRODUCT POSES ADDITIONAL POTENTIAL SIGNIFICANT RISK TO THE SUBJECT, THEN THE RECHALLENGE MUST BE APPROVED IN ADVANCE BY THE U.S. CLINICAL MONITOR AS PER DOSE MODIFICATION GUIDELINES IN THE PROTOCOL.
	**Consistency with Trial Treatment Profile**	Is the clinical/pathological presentation of the AE consistent with previous knowledge regarding the Merck product or drug class pharmacology or toxicology?
The assessment of relationship will be reported on the case report forms /worksheets by an investigator who is a qualified physician according to his/her best clinical judgment, including consideration of the above elements.		
**Record one of the following**		**Use the following scale of criteria as guidance (not all criteria must be present to be indicative of a Merck product relationship).**
**Yes, there is a reasonable possibility of Merck product relationship.**		There is evidence of exposure to the Merck product. The temporal sequence of the AE onset relative to the administration of the Merck product is reasonable. The AE is more likely explained by the Merck product than by another cause.
**No, there is not a reasonable possibility Merck product relationship**		Subject did not receive the Merck product OR temporal sequence of the AE onset relative to administration of the Merck product is not reasonable OR there is another obvious cause of the AE. (Also entered for a subject with overdose without an associated AE.)

7.6 Overall PI Responsibility for Reporting Adverse Events

All Adverse Events will be reported to regulatory authorities, IRB/IECs and investigators in accordance with all applicable global laws and regulations.

7.7 Adverse Event Characteristics

- **CTCAE term (AE description) and grade:** The descriptions and grading scales found in the revised NCI Common Terminology Criteria for Adverse Events (CTCAE) version 4.0 will be utilized for AE reporting. All appropriate treatment areas should have access to a copy of the CTCAE version 4.0. A copy of the CTCAE version 4.0 can be downloaded from the CTEP web site http://ctep.cancer.gov/protocolDevelopment/electronic_applications/ctc.htm.

- **For expedited reporting purposes only:**
  - AEs for the agent(s) that are listed above should be reported only if the adverse event varies in nature, intensity or frequency from the expected toxicity information which is provided.
  - Other AEs for the protocol that do not require expedited reporting are outlined in the next section (Expedited Adverse Event Reporting) under the sub-heading of Protocol-

Specific Expedited Adverse Event Reporting Exclusions.

- **Attribution** of the AE:
  - Definite – The AE *is clearly related* to the study treatment.
  - Probable – The AE *is likely related* to the study treatment.
  - Possible – The AE *may be related* to the study treatment.
  - Unlikely – The AE *is doubtfully related* to the study treatment.
  - Unrelated – The AE *is clearly NOT related* to the study treatment.

7.8 Expedited Adverse Event Reporting

7.8.1 Investigators **must** report to the Overall PI any serious adverse event (SAE) that occurs after the initial dose of study treatment, during treatment, or within 90 days of the last dose of treatment on the local institutional SAE form.

7.8.2 DF/HCC Expedited Reporting Guidelines

Investigative sites within DF/HCC and DF/PCC will report SAEs directly to the DFCI Office for Human Research Studies (OHRS) per the DFCI IRB reporting policy.

Attribution	DF/HCC Reportable AEs				
	**Gr. 2 & 3 AE Expected**	**Gr. 2 & 3 AE Unexpected**	**Gr. 4 AE Expected**	**Gr. 4 AE Unexpected**	**Gr. 5 AE Expected or Unexpected**
Unrelated Unlikely	Not required	Not required	5 calendar days#	5 calendar days	24 hours*
Possible Probable Definite	Not required	5 calendar days	5 calendar days#	5 calendar days	24 hours*
# If listed in protocol as expected and not requiring expedited reporting, event does not need to be reported.					
* For participants enrolled and actively participating in the study ***or*** for AEs occurring within 30 days of the last intervention, the AE should be reported within 24 business hours of learning of the event.					

The Overall PI will submit SAE reports from outside institutions to the DFCI OHRS according to DFCI IRB policies and procedures in reporting adverse events.

7.8.3 Expedited Reporting to the Food and Drug Administration (FDA)

The Overall PI, as study sponsor, will be responsible for all communications with the FDA. The Overall PI will report to the FDA, regardless of the site of occurrence, any serious adverse event that meets the FDA's criteria for expedited reporting following the reporting requirements and timelines set by the FDA.

7.8.4 Expedited Reporting to Hospital Risk Management

Participating investigators will report to their local Risk Management office any participant safety reports or sentinel events that require reporting according to institutional policy.

7.8.5 Routine Adverse Event Reporting

All Adverse Events **must** be reported in routine study data submissions to the Overall PI on the toxicity case report forms. **AEs reported through expedited processes (e.g., reported to the IRB, FDA, etc.) must also be reported in routine study data submissions.**

8. PHARMACEUTICAL INFORMATION

8.1 Pembrolizumab

Pembrolizumab is a potent and highly selective humanized mAb designed to block the interaction between PD-1 and its ligands, PD-L1 and PD-L2. MK-3475 potently blocks binding to both ligands with half maximal inhibitory concentration (IC50) values below 1 nM. MK-3475 enhances T cell responses in human donor blood cell cultures with an EC50 of ~0.1 to 0.3 nM.

Additional detailed information on pembrolizumab is available in the Investigator's Brochure.

8.2 Description

Pembrolizumab (MK-3475) is a humanized anti-PD-1 mAb of the IgG4/kappa isotype with a stabilizing S228P sequence alteration in the fragment crystallizable (Fc) region. MK-3475 binds to human PD-1 and blocks the interaction between PD-1 and its ligands. The theoretical molecular weight of the polypeptide is 146,288 Da and its theoretical pI is 7.5. The parental murine anti-human PD-1 antibody (hPD-1.09A) was produced by immunizing mice with hPD-1 DNA. The MK-3475 antibody was generated by humanization of the parental antibody by the Medical Research Council (Cambridge, UK) using complementarily-determining region (CDR) grafting technology (U.S. Patent No. 5,225,539). The gene segments encoding the variable heavy and light chains of MK-3475, as well as human IgG4, were codon-optimized, synthesized, and ligated into a vector.

A single expression plasmid, pAPD11V1-GA was constructed for the expression of both the heavy and light antibody chains of MK-3475. The nucleotide sequences encoding the heavy and light chains, along with their respective promoters and poly A signal sequences have been confirmed by DNA sequence analysis. The pAPD11V1-GA expression vector was subsequently used to transfect CHO-DXB-11 cells for the development of the MK-3475-producing cell line.

The theoretical molecular weight of the polypeptide is 146,288 Da and its theoretical pI is 7.5. Pembrolizumab exhibits linear pharmacokinetics at dose levels of clinical relevance (1-10 mg/kg). It exhibits low clearance and limited volume of distribution that is typical for therapeutic antibodies. Mean estimated t1/2 values are 14.1-21.6 days.

The nomenclature of pembrolizumab is provided in the table below:

Code Name	MK 3475 (Anti-PD-1)
Other Code Name	SCH 900475 (Anti-PD-1)
Chemical Name	Humanized X PD-1_mAb (H409A11) IgG4
CAS Number	1374853-91-4
CAS Name	Anti-(human protein PDCD1 (programmed cell death 1)) immunoglobulin G4 (human-Mus musculus monoclonal heavy chain) disulfide with human-Mus musculus monoclonal light chain, dimer
Generic Name	Not available
Commercial Name	Pembrolizumab

8.3 Form

The investigator shall take responsibility for and shall take all steps to maintain appropriate records and ensure appropriate supply, storage, handling, distribution and usage of investigational product in accordance with the protocol and any applicable laws and regulations.

Pembrolizumab is supplied as a clear to opalescent solution that is essentially free of extraneous particles and may contain proteinaceous particulates. One dosage form of pembrolizumab will be provided by Merck in Type I glass vials intended for single use only as summarized in the following table:

**Product Name & Potency**	**Dosage Form**
Pembrolizumab 100 mg/ 4mL	Solution for Injection

Pembrolizumab solution for infusion is a sterile, non-pyrogenic, aqueous, preservative-free solution. Pembrolizumab solution for infusion contains an excess fill of 6.25 mg (equivalent to 0.25 mL solution) to ensure the recovery of label claim of 100 mg pembrolizumab per vial (equivalent to 4.0 mL of solution).

8.4 Packaging and Labeling Information

Clinical supplies will be affixed with a clinical label in accordance with regulatory requirements.

8.5 Clinical Supplies Disclosure

This trial is open-label; therefore, the subject, the trial site personnel, the Overall PI and/or designee are not blinded to treatment. Drug identity (name, strength) is included in the label text; random code/disclosure envelopes or lists are not provided.

8.6 Storage, Handling and Preparation Requirements

As specified in the Pharmacy Manual for pembrolizumab as provided by the Merck.

Clinical supplies must be stored in a secure, limited-access location under the storage conditions specified on the label.

Receipt and dispensing of trial medication must be recorded by an authorized person at the trial site.

Clinical supplies may not be used for any purpose other than that stated in the protocol.

8.7 Administration

Pembrolizumab will be administered as a 30 minute IV infusion using an infusion pump (treatment cycle intervals may be increased due to toxicity as described in Section 6). Sites should make every effort to target infusion timing to be as close to 30 minutes as possible. However, given the variability of infusion pumps from site to site, a window of -5 minutes and +10 minutes is permitted (i.e., infusion time is 30 minutes: -5 min/+10 min). Attach the infusion line to the pump and prime the line, either with normal saline (at least 25 mL) or with infusion solution as per local SOP, before starting the infusion. Maximum infusion rate should not exceed 6.7 ml/min through a peripheral or indwelling catheter. Use 30 mL normal saline to flush the infusion line at the end of the infusion if institutional guidelines allow. For more details, please see pharmacology manual.

Unused infusion solution should not be used for another infusion of the same participant or different participant.

DO NOT administer the product as an intravenous push or bolus.

DO NOT combine, dilute or administer it as an infusion with other medicinal products.

A central line is not required for Pembrolizumab administration, but may be used if available.

The following infusion set materials are compatible with Pembrolizumab

- PVC infusion set that is plasticized using Di-2-ethylhexyl Terephthalate DEHP
- PVC and tri-(2-ethylhexyl) trimellitate (TOTM) infusion set
- Polyethylene lined PVC infusion set
- Polyrethane
- Plybutadiene A sterile, non-pyrogenic, low-protein binding 0.2 to 5 µm in-line filter made of polyethersulfone (PES) or polysulfone must be used during administration to remove any adventitious particles. If the infusion set does not contain a 0.2 to 5 µm in-line filter, it is recommended to use a 0.2 to 5 µm add-on filter which may contain an extension line (the materials of the extension line and filter should be as mentioned above).

A sterile, non-pyrogenic, low-protein binding 0.2 to 5 µm in-line filter made of polyethersulfone

(PES) or polysulfone must be used during administration to remove any adventitious particles. If the infusion set does not contain a 0.2 to 5 µm in-line filter, it is recommended to use a 0.2 to 5 µm add-on filter which may contain an extension line (the materials of the extension line and filter should be as mentioned above).

8.8 Ordering

Participating Institutions will order their own investigational agent (Pembrolizumab) directly from Merck using the Drug Supply Request Form. Please allow for 3 weeks for drug to arrive after the order is submitted. The Participating Institution will ensure that the pharmacy will be able to receive and store the agent according to state and federal guidelines. The local IRB should be kept informed of who will supply the agent (i.e., Merck pharmaceuticals Inc.) so that any regulatory responsibilities can be met in a timely fashion.

8.9 Accountability

The investigator is responsible for keeping accurate records of the clinical supplies received from Merck or designee, the amount dispensed to and returned by the subjects and the amount remaining at the conclusion of the trial.

8.10 Destruction and Return

Upon completion or termination of the study, all unused and/or partially used investigational product will be destroyed at the site per institutional policy. It is the Investigator's responsibility to arrange for disposal of all empty containers, provided that procedures for proper disposal have been established according to applicable federal, state, local and institutional guidelines and procedures, and provided that appropriate records of disposal are kept.

9. BIOMARKER, CORRELATIVE, AND SPECIAL STUDIES

9.1 Exploratory Laboratory Correlative Studies

As outlined above in section 2.4, we will characterize pre-treatment samples and any post-treatment samples that are resected and available using whole exome sequencing (WES), RNA-sequencing, immunohistochemistry for immune cell markers and in situ hybridization for PD-1 and PD-L1 mRNA.

9.2 Background

Comprehensive genomic characterization of samples collected as part of our proposed clinical trial will allow us to identify biomarkers of response and provide insight into mechanisms of resistance to immunotherapy. Pre-treatment formalin fixed paraffin embedded (FFPE) tissue samples will be collected within 30 days after enrollment and post-treatment FFPE samples will be collected in patients that progress on treatment if surgery is clinically warranted and if the sample tissue is available. Analytic tools for WES have been developed for our prior studies[14,21-23] and will be used to elucidate the genomic evolutionary profiles in matched tumor samples [20]. As previously described in our work showing that meningioma cells express PD-L1, we will characterize PD-L1 expression, tumor infiltrating lymphocytes and PD-1 expressing lymphocytes.

9.3 Methodology

9.3.1 RNAscope assay and evaluation

FFPE sections will be hybridized separately with Probe-Hs-PDL1-v2 and a PD-1 specific probe and then visualized. Probe-DapB and Probe-Hs-PPIB will be used as negative and positive controls respectively. Stained slides will be digitally scanned, then visually evaluated by two pathologists. We will use an open-source CellProfiler image analysis software (http://www.cellprofiler.org/) to analyze the number of dots per cell using a customized analysis pipeline that we have developed, optimized and tested. (http://www.cellprofiler.org/published_pipelines.shtml).

9.3.2 Immunohistochemistry and evaluation

The following primary antibodies will be used to characterize the tissue pre and post-treatment FFPE tissue sections from this study: LCA (1:600 dilution, Cat# M0701; Dako, CA), CD3 (1:250 dilution, Cat# A0452; Dako, CA), CD4 (1:80 dilution, Cat# M7310; Dako, CA), CD8 (1:100 dilution, Cat# M7103; Dako, CA), CD20 (Ready to Use, Cat# N1502 RTU; Dako, CA), FOXP3 (1:50 dilution, Cat# 320102; Biolegend, CA), PD-1 (1:300 dilution, Cat# 315M-95; Cell Marque, CA), PD-L1 (1:36 dilution, Cat# 10084-R015; Sinobiological, China), PD-L1 (1:125 dilution, Cat# 405.9A11; courtesy of Gordan Freeman lab DFCI). We will use an Aperio scanner to acquire digital images from all stained slides. Digital images will be scored using Aperio ImageScope software.

Furthermore, PD-L1 expression will be measured from any remaining archival tissue and correlated with response. PD-L1 expression will be measured by the QualTek assay as well as with RNA-Seq as specified below in 9.3.3.

9.3.3 RNA-sequencing

RNA Sequencing from FFPE includes plating, poly-A selection and cDNA synthesis, library preparation, sequencing, sample identification QC, and data storage. RNA will be extracted from tissue sections and RNA libraries will be prepared for sequencing by NEBNext Ultra RNA Library Prep Kit for Illumina (New England BioLabs, Ipswich, MA), including the removal of large and small RNA, synthesis of cDNA, and construction of cDNA libraries. Libraries will be barcoded using NEBNext Multiplex Oligos for Illumina (NEB). Libraries will be sequenced using Illumina HiSeq 2500, with paired-end 100bp reads. We will follow the analytic pipelines as previously described[24-27]. Paired-end reads will be aligned to UCSC human transcriptome 19 (hg19) using TopHat (Bowtie v2.0.9). Alignment quality and read distribute will be assessed via SAMtools (v0.1.19). Transcript assembly will be conducted using cufflinks (v2.2.1). Normalized expression data will be generated from aligned BAM files using cuffnorm and cuffdiff. Statistically significant genes differentially expressed between responder samples and non-responders will be determined.

9.3.4 Whole exome sequencing

Whole-exome sequencing will be performed on DNA isolated from unstained FFPE tissue sections and matched DNA from blood. Sequencing will be performed to a mean depth of 100X, and analysis proceeded as previously described [14,19,20,28]. Raw sequencing data will be processed using the Picard tools pipeline and the Genome Analysis Toolkit (GATK). Mutation analysis for single nucleotide variants (SNV) will be performed using MuTect v1.1.4, indel calling performed using the GATK SomaticIndelDetector tool; SNVs and indels annotated using Oncotator. To analyze somatic copy number alterations from whole exome data, we use the ReCapSeg algorithm, which assesses homologue-specific copy ratios (HSCRs) from segmental estimates of multipoint allelic copy-ratios at heterozygous loci incorporating the statistical phasing software (BEAGLE) and population haplotype panels (HAPMAP3). Phlyogenetic analysis will be performed as previously described [20]. Targeted sequencing of panels of genes will be performed in a select subset of cases as previously described [29].

9.3.5 MRI Studies

All MRIs including baseline and any follow-up time points during the study duration should be sent to Dr. Elizabeth Gerstner. These studies can be batched but should be sent at least every 3 months. The MRI can be sent in the mail via CD/DVD to Dr. Elizabeth Gerstner at the address below or electronically transferred via Partners Secure File Transfer at: https://transfer.partners.org/courier/web/1000@/wmLogin.html

For CD/DVD shipping, please label DVD with patient ID and time point. Send to:

Elizabeth Gerstner, MD

Yawkey 9E
55 Fruit Street
Boston, MA
02114

9.3.5.3 Statistical Considerations

Exploratory analyses will be conducted. As data permit, analyses of the MRI parameters will be summarized by descriptive statistics, including mean, median and standard deviation. Differences in these parameters between responders and non-responders will be compared with parametric or nonparametric techniques as permitted by the data. For correlative analyses, Cox proportional hazards model will be used to explore the relationship between parameters measured at baseline and PFS and OS. Logistic regression will be used to explore the relationship between parameters measured at baseline and the binary outcome of alive and progression-free at 6 months (APF6).

Overall, the plan is to determine whether the baseline measurements are prognostic for the entire group. We will determine whether there is an association between the baseline measurements and response adjusting for the patient cohort. A priori we do not think that the association between the baseline measurements and response will depend on the type of mutation and treatment. However, if the association between baseline measurements and response are not found to be statistically significantly associated, we will do subgroup analyses to determine if there might be differences in the association between baseline variables and response among the groups. Specifically, we will do perform the analysis for each group separately. We will also do tests for interaction between the cohort group and the baseline variable, though these will likely not have sufficient power due to the small sample sizes.

The overall plan for determining whether a change in the parameters from baseline is associated with response is to first evaluate the association with the change between baseline and first MRI time point (6 weeks into therapy). An additional analysis will be done that uses all the available MRI assessment time points. To account for multiple MRI time points, we will treat the change in MRI from baseline as a time-dependent variable (which could change at each assessment).

9.4 Collection and Shipping of Tissue Specimen(s)

Collection, Processing and Handling of Archived Specimens: For each resection, all H&E slides shall be made available. In addition 1 FFPE block shall be forwarded to the address within 30 days after patient enrollment. If an institution is unable to provide a tissue block, 20 unstained slides five-micron sections mounted on charged glass slides will be required. Slides will need to be cut with a new blade and using a fresh water bath to avoid contamination. Label the slides with patient ID number, accession number, and order of sections *(include thickness of section if applicable).* Do not bake or place covers slips on the slide.

Shipping of Tissue Specimen(s): Shipment on Monday through Thursday by overnight service to assure receipt is encouraged. Do not ship specimens on Fridays or Saturdays.

Nicole Morris
Massachusetts General Hospital Cancer Center Protocol Office
326 Cambridge Street, Floor 3
Boston, MA 02114

9.5 Collection and Shipping of Blood Specimen(s)

Shipping of Blood Specimen(s): Collect two 10 mL of venous blood at pre-registration and then two 10 mL every 6 weeks in lavender top (EDTA anticoagulant) vacutainer tube(s). The tubes should be inverted approximately 8-10 times to mix the EDTA. Refrigerate (please do not freeze) sample until shipping. The sample should be placed in a biohazard bag and shipped according to IATA guidelines within 2-3 hours of the blood being drawn on a cold pack (please do not use dry ice)**.** For DFCI samples, call the MGH operator at 617-726-2000, ask to page 2-3389 and 2-1928, and leave a brief message indicating that the sample has been shipped. For any questions regarding samples, please contact Nathaniel Goss and/or Brian Shaw.

When samples are shipped and for any concerns regarding shipments, please page Nathaniel Goss at 2-3389.

Brastianos Lab
Nathaniel Goss
Simches Research Center
185 Cambridge Street
Boston, MA 02114

10. STUDY CALENDAR

Scans and x-rays must be done <u><</u>3 weeks prior to the start of therapy. All eligibility labs are to be drawn within 14 days of day 1 (as stated in section 3.1.9). Assessments must be performed prior to administration of any study agent. Study assessments and agents should be administered within <u>+</u> 3 days of the protocol-specified date, unless otherwise noted.

**Trial Period:**	**Screening Phase[a]**	**Treatment Cycles (21 day cycles)[b,c]**								**End of Treatment[d]**	**Post-Treatment**		
						To be repeated beyond 8 cycles[v]							
Treatment Cycle/Title:	Study Screening (Visit 1)	1	2	3	4	5	6	7	8	Discon	Safety Follow-up[e]	Active Followup[f]	Longterm Followup[g]
Scheduling Window (Days):	-14 to 0		± 3	± 3	± 3	± 3	± 3	± 3	± 3	At time of Discon	30 days post discon	Every 6 weeks	Every 3 months
**Administrative Procedures**													
Informed Consent[h]	X												
Inclusion/Exclusion Criteria[i]	X												
Demographics and Medical History[j]	X												
Prior and Concomitant Medication Review[k]	X												
Pembrolizumab Administration		X	X	X	X	X	X	X	X				
Post-study anticancer therapy status[f]										X	X	X	X
Survival Status[g]												X	X
**Clinical Procedures/Assessments**													
Review Adverse Events[l]	X	X	X	X	X	X	X	X	X	X	X		
Full Physical Examination[m]	X	X	X	X	X	X	X	X	X	X	X		
Vital Signs and Weight[n]	X	X	X	X	X	X	X	X	X	X	X		
ECOG Performance Status[o]	X	X	X	X	X	X	X	X	X	X	X		
**Laboratory Procedures/Assessments: analysis performed by LOCAL laboratory**													
Pregnancy Test – Urine or Serum β-HCG[p]	X												
PT/INR and aPTT[q]	X									X			
CBC with Differential[r]	X	X	X	X	X	X	X	X	X	X			
Comprehensive Serum Chemistry Panel[s]	X	X	X	X	X	X	X	X	X	X			
TSH[t]	X	X	X		X		X		X	X			
**Efficacy Measurements**													
Tumor Imaging[u]	X			X		X		X		X			
**Tumor Biopsies/Archival Tissue Collection/Correlative Studies Blood**													
Archival Tissue[w]	X												

**Trial Period:**	**Screening Phase[a]**	**Treatment Cycles (21 day cycles)[b,c]**								**End of Treatment[d]**	**Post-Treatment**		
						To be repeated beyond 8 cycles[v]							
Treatment Cycle/Title:	Study Screening (Visit 1)	1	2	3	4	5	6	7	8	Discon	Safety Follow-up[e]	Active Followup[f]	Longterm Followup[g]
Scheduling Window (Days):	-14 to 0		± 3	± 3	± 3	± 3	± 3	± 3	± 3	At time of Discon	30 days post discon	Every 6 weeks	Every 3 months
Research Blood[x]	X	X		X		X		X		X			

[a]All screening procedures to be performed within 14 days of start of study treatment, except scans which can be obtained within 21 days of starting study treatment, and informed consent may occur up to 28 days prior to start of study treatment
[b]C1D1 and Day 1 of subsequent cycles: For C1D1 screening assessments may serve as day 1 assessments if performed within 7 days before start of study treatment, except serum pregnancy (within 72 hours of drug) and except in the event that there are any indications that the participant's condition is deteriorating for which laboratory evaluations should be repeated within 48 hours prior to initiation. C1D1 labs must meet eligibility criteria. For all subsequent cycles, required assessments should be performed within 3 days of scheduled cycle day 1.
[c]Study drug therapy administration windows include ± 3 days for each agent.
[d]End of Tx: End of treatment assessments to be performed within 7 days after last day drug administration or within 7 days after decision to end treatment. Assessments may continue for ongoing reportable adverse events.
[e]30-Day Post Drug: A contact/visit is to be performed at 30 days (+/-5 days) after the last study drug is given. This may be performed via documented phone conversation with a study nurse or clinician or a clinic visit. All participants will be followed until resolution or stabilization of any serious or reportable adverse events occurring during treatment or starting within 30 days of last study drug.
[f]Active Follow Up: In participants who discontinue study treatment without documented disease progression, every effort should be made to continue monitoring their disease status by radiologic imaging every 6 weeks (+/- 7 days) until (1) the start of new anti-cancer treatment, (2) documented disease progression, or (3) death whichever occurs first.
[g]Long Term Follow Up: participants will be followed every 3 months (+/- 1 month) via contact or medical record review until death for post-treatment therapies, reason for stopping those therapies and survival.
[h]Informed Consent: Informed Consent may be obtained within 28 days of start of study treatment
[i]Inclusion/Exclusion Criteria: source documentation providing investigator's confirmation that the participant had met all eligibility criteria must be available prior to registration.
[j]Demographics and Medical History: to include review of treatment history for cancer, any ongoing medical conditions and medical history pertaining to eligibility on study and involvement during study.
[k]Concomitant medications and reason for administration should be documented in the case history from within 28 days before starting study treatment up to the 30-Day Post Drug Visit.
[l]Adverse event assessment: adverse events experienced by participants will be collected and recorded from the first dose of study treatment up to the 30-Day Post Drug Visit of the last dose of study medication (+/- 5 days depending on when 30-Day Post Drug visit/contact occurs) and all SAEs (related and unrelated to trial treatment) / ECIs up to 90 days after the last dose of trial
[m]Full Physical Exam (including neurologic exam): to be completed by the investigator or qualified designee at screening, C1D1 and start of all subsequent cycles.
[n]Vital signs: weight, heart rate, blood pressure, respiration rate, temperature. Vital signs must be performed prior to administration of treatment on treatment days. Height required only at screening.
[o]Performance Status: See Appendix A for ECOG scale.
[p]Pregnancy Test: For women of child bearing potential, a urine pregnancy test should be performed within 72 hours of first dose of study treatment. If urine pregnancy results are positive or cannot be confirmed as negative, a serum pregnancy test will be required.
[q]Coagulation – PT/INR, PTT required at screening only.
[r]Hematology: erythrocytes (RBC), hemoglobin, hematocrit, platelets, total WBC plus differential (neutrophils/ANC, lymphocytes, monocytes, eosinophils, basophils).
[s]Serum Chemistry: albumin, alkaline phosphatase (ALP), bicarbonate (HCO3), BUN, calcium, chloride, creatinine, glucose, lactate dehydrogenase (LDH) magnesium, phosphorous, potassium, SGOT (AST), SGPT (ALT), sodium, total protein, total bilirubin (if total bilirubin is greater than the upper limit of normal, direct should be performed), and uric acid.
[t]TSH will be performed at baseline, week 1, beginning of cycle 2 and then every 2 cycles. TSH results do not need to be back prior to dosing
[u]Brain MRI will be performed every 2 cycles. In the rare circumstance where patients have extracranial metastases, chest/abd/pelvis CT will also be obtained every 2 cycles. If pembrolizumab is held or interrupted, the scan schedule should be maintained at every 2 cycles.
[v]As per section 5.12, completed 24 months of uninterrupted treatment with pembrolizumab or 35 administrations of study medication, whichever is later
[w]Tissue collected as part of clinical care (e.g. from prior biopsy, craniotomy) will be collected. Tissue needs to be submitted within 30 days after patient enrollment. See Section 9 for specimen collection requirements
[x]Blood will be collected at screening every 2 cycles (two 10 mL EDTA/lavender top tube) and at the end of treatment

11. MEASUREMENT OF EFFECT

11.1 Antitumor Effect – Intracranial Disease

For the purposes of this study, participants should be re-evaluated for intracranial response every 6 weeks. In addition to a baseline scan, confirmatory scans should also be obtained at 6 weeks following initial documentation of objective response. Response and progression will be evaluated in this study using the modified RANO Response Criteria.

11.2 Definitions

Evaluable for objective response. Only those participants who have measurable CNS disease present at baseline, have received at least one cycle of therapy, and have had their disease re-evaluated will be considered evaluable for target disease response. These participants will have their response classified according to the definitions stated below. (Note: Participants who exhibit objective disease progression prior to the end of cycle 1 will also be considered evaluable.)

Evaluable non-measurable evaluable disease. Participants who have lesions present at baseline that are evaluable but do not meet the definitions of measurable disease, have received at least one cycle of therapy, and have had their disease re-evaluated will be considered evaluable for non-target disease. The response assessment is based on the presence, absence, or unequivocal progression of the lesions.

11.2.1 Disease Parameters

Measurable disease. Measurable lesions are defined as those that can be accurately measured in at least one dimension (longest diameter to be recorded) as ≥10 mm with MRI. All tumor measurements must be recorded in millimeters (or decimal fractions of centimeters).

For multifocal intracranial disease, no more than 5 target measurable lesions (each ≥10mm in diameter in both dimensions) should be selected for measurement. Target lesions should be selected on the basis of their size (lesions with longest diameter), be representative of other lesions and lend themselves to reproducible repeated measurements.

Non-measurable disease. Unidimensionally measurable lesions, masses with margins not clearly defined, lesions with maximal diameter < 1cm.

11.2.2 Methods for Evaluation of Disease

All measurements should be taken and recorded in metric notation using a ruler, calipers, or a digital measurement tool. All baseline evaluations should be performed as closely as

possible to the beginning of treatment and never more than 3 weeks before the beginning of the treatment.

The same method of assessment and the same technique should be used to characterize each identified and reported lesion at baseline and during follow-up. Imaging-based evaluation is preferred to evaluation by clinical examination unless the lesion(s) being followed cannot be imaged but are assessable by clinical exam.

11.3 Response Criteria

11.3.1.1 Evaluation of Intracranial Lesions

Complete Response (CR): Requires all of the following:

- complete disappearance of all enhancing measurable and nonmeasurable disease sustained for at least 4 weeks
- no new lesions
- stable or decreasing corticosteroids (the steroid dose at the time of the scan evaluation should be no greater than the dose at time of baseline scan)
- stable or improved clinically

Participants with non-measurable disease cannot have a complete response. The best possible response is stable disease.

Partial Response (PR): All of the following criteria must be met:

- Greater than or equal to 50% decrease compared to baseline in the sum of products of perpendicular diameters of all measurable enhancing lesions sustained for at least 4 weeks. In the absence of a confirming scan 4 weeks later, this scan will be considered only stable disease.
- no progression of non-measurable disease.
- no new lesions
- stable or reduced corticosteroid dose (the steroid dose at the time of the scan evaluation should be no greater than the dose at time of baseline scan)
- stable or improved clinically

Progressive Disease (PD): At least one of the following must be true:

- ≥25% increase in the sum of products of all measurable lesions over smallest sum observed (over baseline if no decrease) using the same techniques as baseline
- clear worsening of any nonmeasurable disease*
- appearance of any new enhancing lesion/site
- clear clinical worsening (unless clearly unrelated to this cancer, e.g. seizures, medication side effects, complications of therapy, cerebrovascular events, infecion)
- failure to return for evaluation due to death or deteriorating condition

* Progression of non-measurable CNS lesions is defined as follows:

- a lesion initially at baseline ≤ 5mm in diameter that increases to ≥ 10mm in diameter, or
- a lesion initially at baseline 6-9 mm in diameter that increases by at least 5 mm in diameter.
- a lesion that is smaller than 10mm that is too complex or has poorly defined margins, that is now unequivocally progressing by more than 25%

Stable Disease (SD): All of the following criteria must be met:

- Does not qualify for CR, PR or progression
- All measurable and non-measurable sites must be assessed using the same techniques as baseline
- Stable clinically.

A summary of the response criteria is in the below:

	Complete Response	Partial Response	Stable Disease	Progressive Disease
T1-Gd+	None	≥ 50% decrease	<50% decrease-<25% increase	≥25% increase*
New Lesion	None	None	None	Present*
Corticosteroids	Stable or decrease	Stable or decrease	Stable or decrease	Stable or increasing
Clinical Status	Stable or improved	Stable or improved	Stable or improved	Decrease*
Requirement for Response	All	All	All	Any*
NA=not applicable<br>#: Progression occurs when any of the criteria with * is present<br>Increase in corticosteroids alone will not be taken into account in determining progression in the absence of persistent clinical deterioriation				

11.3.1.2 Evaluation of Best Overall Response

The best overall response is the best response recorded from the start of the treatment until disease progression/recurrence (taking as reference for progressive disease the smallest measurements recorded since the treatment started). The patient's best response assignment will depend on the achievement of both measurement and confirmation criteria. If a response recorded at one scheduled MRI does not persist at the next regular scheduled MRI, the response will still be recorded based on the prior scan, but will be designated as a non-sustained response. If the response is sustained, i.e. still present on the subsequent MRI at least four weeks later, it will be recorded as a sustained response, lasting until the time of tumor progression. Participants without

measurable disease may only achieve SD or PD as their best "response."

11.3.2 Duration of Response

Duration of overall response: The duration of overall response is measured from the time measurement criteria are met for CR or PR (whichever is first recorded) until the first date that recurrent or progressive disease is objectively documented (taking as reference for progressive disease the smallest measurements recorded since the treatment started, or death due to any cause. Participants without events reported are censored at the last disease evaluation).

Duration of overall complete response: The duration of overall CR is measured from the time measurement criteria are first met for CR until the first date that progressive disease is objectively documented, or death due to any cause. Participants without events reported are censored at the last disease evaluation.

Duration of stable disease: Stable disease is measured from the start of the treatment until the criteria for progression are met, taking as reference the smallest measurements recorded since the treatment started, including the baseline measurements.

11.3.3 Study Continuation Beyond Initial Progressive Disease

Immunotherapeutic agents such as pembrolizumab may produce antitumor effects by potentiating endogenous cancer-specific immune responses which may manifest as initial worsening of enhancement and edema on MRI or CT scans (i.e. pseudo-progression). In addition, the response patterns seen with immunotherapeutics may extend beyond the typical time course of responses seen with cytotoxic agents, and can manifest a clinical response after an initial increase in tumor burden or even the appearance of new lesions. For these reasons, the immune- related response criteria (irRC) have endorsed continuation of study therapy beyond initial radiographic evidence of progression for clinically stable patients undergoing immune based therapies.

Therefore, the following adaptations will be used to assess response for patients treated on this study:

Potential Pseudo-progression: If radiologic imaging shows initial PD, subjects who are not experiencing significant clinical decline, may be allowed to continue study treatment for up to three months. Patients who have radiographic evidence of progression after up to three months (as defined by progression as in 11.3.1.1), or who decline significantly at anytime, will be classified as progressive with the date of disease progression back-dated to the first date that the subject met criteria for progression and such subjects will be discontinued from study therapy. Three months is the most common timeframe for pseudo-progression observed among patients with advanced melanoma or other solid tumors treated with PD-1/PD-L1 immune checkpoint blockade[40].

Among patients on this study with initial radiographic PD, tumor assessment should be

repeated regularly in order to confirm PD with the option of continuing treatment as described below while awaiting radiologic confirmation of progression. If repeat imaging shows a stabilization or reduction in the tumor burden compared to the initial scan demonstrating PD, treatment may be continued / resumed. If repeat imaging after up to three months confirms progressive disease, then the date of disease progression will be the first date the subject met criteria for progression and subjects will be discontinued from study therapy. Subjects who have confirmed disease progression will discontinue study medication and enter the follow up/survival phase of the study. In determining whether or not the tumor burden has increased or decreased, investigators should consider all target lesions as well as non-target lesions.

Tumor Enhancement to Define Progression: RANO expanded the previously utilized Macdonald criteria[41] to include the development of “significantly” increased T2 or FLAIR abnormality in the definition of progressive disease because such changes can be a major component defining radiographic progression following therapeutic use of VEGF/VEGFR-targeting therapeutics which are known to elicit potent anti-permeability changes that limit contrast uptake. However, immune based therapies are expected to be associated with inflammatory changes that may include edema. Therefore, our study will define radiographic progressive disease by assessment of enhancing tumor burden only and will not include assessment of T2 or FLAIR changes as outlined in RANO because:

- There is no expectation that immunotherapy agents including PD-1 inhibitors will falsely diminish enhancing tumor burden as has been noted with anti-angiogenic therapies; and
- Immune based therapies are expected to induce inflammatory responses which may be associated with increased edema and T2/FLAIR changes. Such radiographic finding may inaccurately be interpreted to represent tumor progression (i.e. pseudoprogression).

Treatment beyond evidence of radiographic PD: In subjects who have initial evidence of radiographic PD, it is at the discretion of the treating physician whether to continue a subject on study treatment for up to three months pending confirmation of PD on follow-up imaging. This clinical judgment decision should be based on the subject’s overall clinical condition, including performance status, clinical symptoms, and laboratory data. Subjects may receive study treatment while waiting for confirmation of PD if they are not experiencing significant clinical decline and if:

- The subject is believed to demonstrate clinical benefit from the study regimen as determined by the treating physician;
- The subject is adequately tolerating study therapy.

When feasible, study therapy should not be discontinued until radiographic progression is confirmed. This allowance to continue treatment despite initial radiologic progression takes into account the observation that some subjects can have a transient tumor flare in the first few months after the start of immunotherapy, but with subsequent disease response[42]. Subjects that are exhibiting significant neurologic decline are not required to

have repeat imaging for confirmation of progressive disease.

Participants with progressive radiographic findings are encouraged to undergo surgical intervention in order to delineate pseudo-progression due to inflammation associated with PD-1 blockade from true tumor progression. Participants with histopathologic findings of significant immune infiltrate and evolving gliosis will be allowed to continue study therapy. In contrast, those with clear evidence of progressive tumor by histopathologic evaluation will be defined as progressive and discontinued from study therapy. For such patients, the date of tumor progression will be the first date the participant met radiographic criteria for PD.

11.3.4 Response Review

All brain MRI scans will be reviewed centrally by the DF/HCC Tumor Imaging Metrics Core (TIMC). CNS response will be assessed centrally using the criteria outlined in Section 11.0.

11.4 Antitumor Effect – Extracranial lesion assessments

For patients that have metastatic meningioma, participants should be re-evaluated for extracranial response every 6 weeks. In addition to a baseline scan, confirmatory scans should also be obtained 6 weeks following initial documentation of objective response.

Response and progression will be evaluated in this study using the new international criteria proposed by the Response Evaluation Criteria in Solid Tumors (RECIST) guideline (version 1.1) [*Eur J Ca* 45:228-247, 2009]. Changes in the largest diameter (unidimensional measurement) of the tumor lesions and the shortest diameter in the case of malignant lymph nodes are used in the RECIST criteria.

11.4.1 Definitions

Evaluable for Target Disease response. Only those participants who have measurable disease present at baseline, have received at least one cycle of therapy, and have had their disease re-evaluated will be considered evaluable for target disease response. These participants will have their response classified according to the definitions stated below. (Note: Participants who exhibit objective disease progression prior to the end of cycle 1 will also be considered evaluable.)

Evaluable Non-Target Disease Response. Participants who have lesions present at baseline that are evaluable but do not meet the definitions of measurable disease, have received at least one cycle of therapy, and have had their disease re-evaluated will be considered evaluable for non-target disease. The response assessment is based on the presence, absence, or unequivocal progression of the lesions.

11.4.2 Disease Parameters

Measurable disease. Measurable lesions are defined as those that can be accurately measured in at least one dimension (longest diameter to be recorded) as ≥ 20 mm by chest x-ray or ≥10 mm with CT scan, MRI, or calipers by clinical exam. All tumor measurements must be recorded in millimeters (or decimal fractions of centimeters).

Malignant lymph nodes. To be considered pathologically enlarged and measurable, a lymph node must be ≥15 mm in short axis when assessed by CT scan (CT scan slice thickness recommended to be no greater than 5 mm). At baseline and in follow-up, only the short axis will be measured and followed.

Non-measurable disease. All other lesions (or sites of disease), including small lesions (longest diameter <10 mm or pathological lymph nodes with ≥10 to <15 mm short axis), are considered non-measurable disease. Bone lesions, leptomeningeal disease, ascites, pleural/pericardial effusions, lymphangitis cutis/pulmonitis, inflammatory breast disease, abdominal masses (not followed by CT or MRI), and cystic lesions are all considered non-measurable.

Note: Cystic lesions that meet the criteria for radiographically defined simple cysts should not be considered as malignant lesions (neither measurable nor non-measurable) since they are, by definition, simple cysts.

‘Cystic lesions’ thought to represent cystic metastases can be considered as measurable lesions, if they meet the definition of measurability described above. However, if non-cystic lesions are present in the same participant, these are preferred for selection as target lesions.

Target lesions. All measurable lesions up to a maximum of 2 lesions per organ and 5 lesions in total, representative of all involved organs, should be identified as **target lesions** and recorded and measured at baseline. Target lesions should be selected on the basis of their size (lesions with the longest diameter), be representative of all involved organs, but in addition should be those that lend themselves to reproducible repeated measurements. It may be the case that, on occasion, the largest lesion does not lend itself to reproducible measurement in which circumstance the next largest lesion which can be measured reproducibly should be selected. A sum of the diameters (longest for non-nodal lesions, short axis for nodal lesions) for all target lesions will be calculated and reported as the baseline sum diameters. If lymph nodes are to be included in the sum, then only the short axis is added into the sum. The baseline sum diameters will be used as reference to further characterize any objective tumor regression in the measurable dimension of the disease.

Non-target lesions. All other lesions (or sites of disease) including any measurable lesions over and above the 5 target lesions should be identified as **non-target lesions** and should also be recorded at baseline. Measurements of these lesions are not required, but the presence, absence, or in rare cases unequivocal progression of each should be noted throughout follow up.

11.4.3 Methods for Evaluation of Disease

All measurements should be taken and recorded in metric notation using a ruler, calipers, or a digital measurement tool. All baseline evaluations should be performed as closely as possible to the beginning of treatment and never more than 3 weeks before the beginning of the treatment.

The same method of assessment and the same technique should be used to characterize each identified and reported lesion at baseline and during follow-up. Imaging-based evaluation is preferred to evaluation by clinical examination unless the lesion(s) being followed cannot be imaged but are assessable by clinical exam.

Clinical lesions. Clinical lesions will only be considered measurable when they are superficial (*e.g.*, skin nodules and palpable lymph nodes) and ≥10 mm in diameter as assessed using calipers (*e.g.*, skin nodules). In the case of skin lesions, documentation by color photography, including a ruler to estimate the size of the lesion, is recommended.

Chest x-ray. Lesions on chest x-ray are acceptable as measurable lesions when they are clearly defined and surrounded by aerated lung; however, CT is preferable.

Conventional CT and MRI. This guideline has defined measurability of lesions on CT scan based on the assumption that CT thickness is 5mm or less. If CT scans have slice thickness greater than 5 mm, the minimum size of a measurable lesion should be twice the slice thickness. MRI is also acceptable in certain situations (*e.g.* for body scans).

Use of MRI remains a complex issue. MRI has excellent contrast, spatial, and temporal resolution; however, there are many image acquisition variables involved in MRI, which greatly impact image quality, lesion conspicuity, and measurement. Furthermore, the availability of MRI is variable globally. As with CT, if an MRI is performed, the technical specifications of the scanning sequences used should be optimized for the evaluation of the type and site of disease. Furthermore, as with CT, the modality used at follow-up should be the same as was used at baseline and the lesions should be measured/assessed on the same pulse sequence. It is beyond the scope of the RECIST guidelines to prescribe specific MRI pulse sequence parameters for all scanners, body parts, and diseases. Ideally, the same type of scanner should be used and the image acquisition protocol should be followed as closely as possible to prior scans. Body scans should be performed with breath-hold scanning techniques, if possible.

11.4.4 Response Criteria

11.4.4.1 Evaluation of Target Lesions

Complete Response (CR): Disappearance of all target lesions. Any pathological lymph nodes (whether target or non-target) must have reduction in short axis to <10 mm.

Partial Response (PR): At least a 30% decrease in the sum of the diameters of target lesions, taking as reference the baseline sum diameters.

Progressive Disease (PD): At least a 20% increase in the sum of the diameters of target lesions, taking as reference the smallest sum on study (this includes the baseline sum if that is the smallest on study). In addition to the relative increase of 20%, the sum must also demonstrate an absolute increase of at least 5 mm. (Note: the appearance of one or more new lesions is also considered progressions).

Stable Disease (SD): Neither sufficient shrinkage to qualify for PR nor sufficient increase to qualify for PD, taking as reference the smallest sum diameters while on study.

11.4.4.2 Evaluation of Non-Target Lesions

Complete Response (CR): Disappearance of all non-target lesions and normalization of tumor marker level. All lymph nodes must be non-pathological in size (<10 mm short axis).

Note: If tumor markers are initially above the upper normal limit, they must normalize for a patient to be considered in complete clinical response.

Non-CR/Non-PD: Persistence of one or more non-target lesion(s) and/or maintenance of tumor marker level above the normal limits.

Progressive Disease (PD): Appearance of one or more new lesions and/or *unequivocal progression* of existing non-target lesions. *Unequivocal progression* should not normally trump target lesion status. It must be representative of overall disease status change, not a single lesion increase.

Although a clear progression of "non-target" lesions only is exceptional, the opinion of the treating physician should prevail in such circumstances, and the progression status should be confirmed at a later time by the review panel (or Principal Investigator).

11.4.4.3 Evaluation of New Lesions

The finding of a new lesion should be unequivocal (i.e. not due to difference in scanning technique, imaging modality, or findings thought to represent something other than tumor (for example, some 'new' bone lesions may be simply healing or flare of pre-existing lesions). However, a lesion identified on a follow-up scan in an anatomical location that was not scanned at baseline is considered new and will indicate PD. If a new lesion is equivocal (because of small size etc.), follow-up evaluation will clarify if it truly represents new disease and if PD is confirmed, progression should be declared using the date of the initial scan on which the lesion was discovered.

11.4.4.4 Evaluation of Best Overall Response

The best overall response is the best response recorded from the start of the treatment until disease progression/recurrence (taking as reference for progressive disease the smallest measurements recorded since the treatment started). The patient's best response assignment will depend on the achievement of both measurement and confirmation criteria.

For Participants with Measurable Disease (*i.e.*, Target Disease)

**Target Lesions**	**Non-Target Lesions**	**New Lesions**	**Overall Response**	**Best Overall Response when Confirmation is Required***
CR	CR	No	CR	≥4 wks Confirmation**
CR	Non-CR/Non-PD	No	PR	≥4 wks Confirmation**
CR	Not evaluated	No	PR	
PR	Non-CR/Non-PD/not evaluated	No	PR	
SD	Non-CR/Non-PD/not evaluated	No	SD	Documented at least once ≥4 wks from baseline**
PD	Any	Yes or No	PD	no prior SD, PR or CR
Any	PD***	Yes or No	PD	
Any	Any	Yes	PD	

* See RECIST 1.1 manuscript for further details on what is evidence of a new lesion.
** Only for non-randomized trials with response as primary endpoint.
*** In exceptional circumstances, unequivocal progression in non-target lesions may be accepted as disease progression.

Note: Participants with a global deterioration of health status requiring discontinuation of treatment without objective evidence of disease progression at that time should be reported as "*symptomatic deterioration.*" Every effort should be made to document the objective progression even after discontinuation of treatment.

For Participants with Non-Measurable Disease (*i.e.*, Non-Target Disease)

**Non-Target Lesions**	**New Lesions**	**Overall Response**
CR	No	CR
Non-CR/non-PD	No	Non-CR/non-PD*
Not all evaluated	No	not evaluated
Unequivocal PD	Yes or No	PD
Any	Yes	PD

* 'Non-CR/non-PD' is preferred over 'stable disease' for non-target disease since SD is

increasingly used as an endpoint for assessment of efficacy in some trials so to assign this category when no lesions can be measured is not advised

11.4.5 Duration of Response

Duration of overall response: The duration of overall response is measured from the time measurement criteria are met for CR or PR (whichever is first recorded) until the first date that recurrent or progressive disease is objectively documented (taking as reference for progressive disease the smallest measurements recorded since the treatment started, or death due to any cause. Participants without events reported are censored at the last disease evaluation).

Duration of overall complete response: The duration of overall CR is measured from the time measurement criteria are first met for CR until the first date that progressive disease is objectively documented, or death due to any cause. Participants without events reported are censored at the last disease evaluation.

Duration of stable disease: Stable disease is measured from the start of the treatment until the criteria for progression are met, taking as reference the smallest measurements recorded since the treatment started, including the baseline measurements.

11.4.6 Progression-Free Survival

Overall Survival: Overall Survival (OS) is defined as the time from randomization (or registration) to death due to any cause, or censored at date last known alive.

Progression-Free Survival: Progression-Free Survival (PFS) is defined as the time from randomization (or registration) to the earlier of progression or death due to any cause. Participants alive without disease progression are censored at date of last disease evaluation.

Time to Progression: Time to Progression (TTP) is defined as the time from randomization (or registration) to progression, or censored at date of last disease evaluation for those without progression reported.

11.4.7 Response Review

All non-CNS scans will be reviewed centrally by the DF/HCC Tumor Imaging Metrics Core (TIMC).

12. END OF TREATMENT/INTERVENTION

12.1 Duration of Treatment

12.1.1 CR, PR, or SD

Patients who are in CR, PR or SD will continue on therapy until progression of disease, excessive toxicity requiring the patient to come off of treatment, or the patient withdraws from treatment. After treatment is discontinued, patients will be followed per the study calendar in section 10.0.

12.1.2 Disease Progression

Remove from protocol therapy any patient with disease progression. Document details, including tumor measurements, on data forms.

After disease progression, patients should be followed for survival per the study calendar (Section 10.0).

12.1.3 Discontinuation of study agent

If the patient discontinues study agent, patients should be followed for disease progression and survival per the study calendar (Section 10.0).

12.2 Definitions and Follow-up Requirements

Definition of ineligible patients: A study participant who is registered to the trial but does not meet all of the eligibility criteria is deemed to be ineligible. Patients who are deemed ineligible may continue protocol treatment, provided the treating physician, study chair, and executive officer agree there are no safety concerns if the patient were to continue protocol treatment. Notification of the local IRB may be necessary per local IRB policies.

Definition of clinical follow-up: The follow-up period where the study participant is no longer receiving treatment, but is still following the study calendar for tests, exams, and correlative endpoints (e.g., specimen collection, quality of life, disease assessments as required by the study).

Definition of survival only follow-up: The follow-up period where the study participant is monitored for long-term endpoints, is no longer receiving study treatment, and is not required to follow the study calendar for tests, exams, and correlative endpoints (e.g. specimen collection, quality of life, disease assessments as required by the study). In this follow-up period, there is a schedule in which case report forms should be submitted, but the physician visits are based on the standard of care.

12.2.1 Follow-up for Ineligible Patients

Study participants who are registered to the trial but deemed ineligible must complete follow-up requirements as specified below.

Baseline, on-study and off-treatment notice data submission required.

12.2.2 Follow-up for Patients Never Receiving Protocol Intervention

Study participants who are registered to the trial but who never go on to receive study intervention must still complete follow-up requirements as specified below.

Baseline, on-study and off-treatment notice data submission required.

12.3 Extraordinary Medical Circumstances

If, at any time the constraints of this protocol are detrimental to the patient's health and/or the patient no longer wishes to continue protocol therapy, protocol therapy shall be discontinued. In this event:

Document the reason(s) for discontinuation of therapy on data forms.

Follow the patient for protocol endpoints as required by the Study Calendar.

13.0 STATISTICAL CONSIDERATIONS

13.1 Study Design

There will be a single phase 2 arm in this study. Patients with recurrent or residual meningioma will be eligible for this trial. There is no planned interim analysis.

13.2 Endpoints

Primary Endpoints:

The primary endpoint is progression-free survival at 6 months (PFS6). PFS6 is defined as not having progressive disease or death within six months of the first day of treatment. Contrast-enhanced cranial magnetic resonance imaging (MRI) will be performed every 6 weeks. Standard response criteria will be used (see Section 11.0).

All patients meeting the eligibility criteria that have signed the consent form and have begun treatment will be considered evaluable for the analysis of primary endpoint.

Secondary Endpoints:

1. Toxicity by CTCAE criteria (grade 3 or higher hematologic toxicity; grade 3 or higher neurologic toxicity)
2. Overall survival
3. Intracranial response as defined by RANO criteria

Exploratory Endpoints:

1. Genetic alterations
2. MRI Imaging changes

13.3 Sample Size, Accrual Rate and Study Duration

The total accrual for this phase II trial will be 26 patients in order to achieve 24 evaluable patients. The estimated accrual rate will be approximately 1 patients per month for this protocol. Twenty-six patients will be accrued within 24-28 months. Based on 6 months of follow-up, the total study duration will be 36 months. Both men and women of all races and ethnic groups are eligible for this trial. Every effort will be made to include patients from minority populations.

13.4 Stratification Factors

NA

13.5 Interim Monitoring Plan

NA

13.6 Analysis of Primary Endpoints

The goal will be to enroll 24 patients. The primary endpoint is the rate of 6 month PFS. The trial will distinguish between 6 month PFS rates of 26% vs. 52%. The historical control of 6 month PFS is based on a recent meta-analysis showing a PFS-6 of 26% in recurrent Grade II/III meningiomas[43] and other studies in this patient population[44-46]. If at least 10 patients demonstrate 6 month PFS, among the 24 evaluable patients, the agent will be considered worthy of further study, yielding at least 88% power to detect a true 6 month PFS rate of at least 52%, using an exact binomial test with a one-sided significance level of 0.1, against the null hypothesis of 26% 6-month PFS.

13.7 Analysis of Secondary Endpoints

<u>Safety and Tolerability</u>: All adverse events recorded during the trial will be summarized for all patients having received one or more doses of pembrolizumab. The proportions of patients with grade-3 or higher hematologic toxicities or grade-3 or higher neurologic toxicities will be presented with 90% exact binomial confidence intervals. The incidence of events that are new or worsening from the time of first dose of treatment will be summarized according to system organ class and/or preferred term, severity (based on CTCAE grade), type of adverse event, and relation to study treatment. Deaths reportable as SAEs and non-fatal serious adverse events will be listed by patient and tabulated by primary system organ class, and type of adverse event.

<u>Overall Survival</u>: The distributions of overall survival will be summarized using the method of Kaplan-Meier. The follow-up of patients who are alive at the time of analysis will be censored at the date of last assessment of vital status. Median OS will be presented and accompanied by 90% confidence intervals estimated using log(-log(survival)) methodology. Survival point estimates at 3 and 6 months will also be presented with confidence intervals.

<u>Intracranial/Extracranial Response</u>: Contrast-enhanced cranial magnetic resonance imaging (MRI) will be performed every 6 weeks. The proportion of patients with intracranial response (CR, PR or SD by RANO) will be presented with a two-sided, 90% exact binomial confidence interval.

13.8 Analysis of Exploratory Endpoints

Biomarkers of blood and tissue: Analyses of blood, imaging, and tissue biomarkers will be based on descriptive statistics, as appropriate for the biomarker.

To examine response according to pre-treatment levels of biomarkers, the study sample will be divided retrospectively according to the primary endpoint of response or non-response. Pre-treatment biomarker levels will be summarized descriptively for the two response groups and compared using Wilcoxon rank-sum tests for markers measured on a continuous scale, or Fisher's exact tests for those measured on a categorical scale. Where appropriate, visualization of the relationship between baseline marker levels and the distributions of PFS or OS will employ Kaplan-Meier estimates stratified by biomarker levels. Medians of the time-to-event endpoints will be shown with two-sided 90% confidence intervals; the distributions of PFS and OS will be compared across biomarker strata using the log-rank test.

Changes in biomarkers between pretreatment and progression or treatment discontinuation will be calculated (post-pre) for each patient and summarized descriptively. In addition, the correlation of changes in tissue biomarkers with changes in measures of peripheral blood markers will be explored graphically, or by appropriate statistical methods based on data availability, to assess associations.

13.9 Reporting and Exclusions

13.9.1 Evaluation of Toxicity

All participants who have received at least one dose of study medication will be evaluable for toxicity from the time of their first treatment.

13.9.2 Evaluation of the Primary Efficacy Endpoint

To be included in the assessment of the primary objective, patients need to receive at least one cycle. If systemic progression occurs prior to the 6 week CNS response timepoint, patient will still be included in the analysis of primary objective.

14 PUBLICATION PLAN

The results should be made public within 24 months of reaching the end of the study. The end of the study is the time point at which the last data items are to be reported, or after the outcome data are sufficiently mature for analysis, as defined in the section on Sample Size, Accrual Rate and Study Duration. If a report is planned to be published in a peer-reviewed journal, then that initial release may be an abstract that meets the requirements of the International Committee of Medical Journal Editors. A full report of the outcomes should be made public no later than three (3) years after the end of the study.

REFERENCES

1. Mahmood A, Qureshi NH, Malik GM. Intracranial meningiomas: analysis of recurrence after surgical treatment. Acta Neurochir (Wien) 1994;126:53-8.
2. Mathiesen T, Lindquist C, Kihlstrom L, Karlsson B. Recurrence of cranial base meningiomas. Neurosurgery 1996;39:2-7; discussion 8-9.
3. Perry A, Stafford SL, Scheithauer BW, Suman VJ, Lohse CM. Meningioma grading: an analysis of histologic parameters. Am J Surg Pathol 1997;21:1455-65.
4. Willis J, Smith C, Ironside JW, Erridge S, Whittle IR, Everington D. The accuracy of meningioma grading: a 10-year retrospective audit. Neuropathol Appl Neurobiol 2005;31:141-9.
5. Sughrue ME, Sanai N, Shangari G, Parsa AT, Berger MS, McDermott MW. Outcome and survival following primary and repeat surgery for World Health Organization Grade III meningiomas. J Neurosurg 2010;113:202-9.
6. Rosenberg LA, Prayson RA, Lee J, et al. Long-term experience with World Health Organization grade III (malignant) meningiomas at a single institution. Int J Radiat Oncol Biol Phys 2009;74:427-32.
7. Wen PY, Quant E, Drappatz J, Beroukhim R, Norden AD. Medical therapies for meningiomas. J Neurooncol 2010;99:365-78.
8. Chamberlain MC, Tsao-Wei DD, Groshen S. Salvage chemotherapy with CPT-11 for recurrent meningioma. Journal of neuro-oncology 2006;78:271-6.
9. Chamberlain MC, Johnston SK. Hydroxyurea for recurrent surgery and radiation refractory meningioma: a retrospective case series. Journal of neuro-oncology 2011;104:765-71.
10. Reardon DA, Norden AD, Desjardins A, et al. Phase II study of Gleevec(R) plus hydroxyurea (HU) in adults with progressive or recurrent meningioma. J Neurooncol 2012;106:409-15.
11. Chamberlain MC. Hydroxyurea for recurrent surgery and radiation refractory high-grade meningioma. J Neurooncol 2012;107:315-21.
12. Chamberlain MC, Glantz MJ. Interferon-alpha for recurrent World Health Organization grade 1 intracranial meningiomas. Cancer 2008;113:2146-51.
13. Norden AD, Raizer JJ, Abrey LE, et al. Phase II trials of erlotinib or gefitinib in patients with recurrent meningioma. Journal of neuro-oncology 2010;96:211-7.
14. Brastianos PK, Horowitz PM, Santagata S, et al. Genomic sequencing of meningiomas identifies oncogenic SMO and AKT1 mutations. Nat Genet 2013;45:285-9.
15. Clark VE, Erson-Omay EZ, Serin A, et al. Genomic analysis of non-NF2 meningiomas reveals mutations in TRAF7, KLF4, AKT1, and SMO. Science 2013;339:1077-80.
16. Du Z, Abedalthagafi M, Aizer AA, et al. Increased expression of the immune modulatory molecule PD-L1 (CD274) in anaplastic meningioma. Oncotarget 2014.
17. Furtner J, Schopf V, Seystahl K, et al. Kinetics of tumor size and peritumoral brain edema before, during, and after systemic therapy in recurrent WHO grade II or III meningioma. Neuro-oncology 2015.
18. Wolchok JD, Kluger H, Callahan MK, et al. Nivolumab plus ipilimumab in advanced melanoma. N Engl J Med 2013;369:122-33.
19. Brastianos PK, Taylor-Weiner A, Manley PE, et al. Exome sequencing identifies BRAF mutations in papillary craniopharyngiomas. Nat Genet 2014;46:161-5.
20. Brastianos PK, Carter SL, Santagata S, et al. Genomic Characterization of Brain

Metastases Reveals Branched Evolution and Potential Therapeutic Targets. Cancer Discov 2015.
21. Brastianos PK, Carter SL, Santagata S, Taylor-Weiner A. Abstract: Genomic characterization of 101 brain metastases and paired primary tumors reveals patterns of clonal evolution and selection of driver mutations. AACR 2014.
22. Brastianos PK, Taylor-Weiner A, Manley PE, et al. Exome sequencing identifies BRAF mutations in papillary craniopharyngiomas. Nat Genet 2014.
23. Shankar GM, Taylor-Weiner A, Lelic N, et al. Sporadic hemangioblastomas are characterized by cryptic VHL inactivation. Acta neuropathologica communications 2014;2:2.
24. Dobin A, Davis CA, Schlesinger F, et al. STAR: ultrafast universal RNA-seq aligner. Bioinformatics 2013;29:15-21.
25. Li B, Dewey CN. RSEM: accurate transcript quantification from RNA-Seq data with or without a reference genome. BMC bioinformatics 2011;12:323.
26. DeLuca DS, Levin JZ, Sivachenko A, et al. RNA-SeQC: RNA-seq metrics for quality control and process optimization. Bioinformatics 2012;28:1530-2.
27. Brooks AN, Yang L, Duff MO, et al. Conservation of an RNA regulatory map between Drosophila and mammals. Genome research 2011;21:193-202.
28. Abedalthagafi MS, Bi WL, Merrill PH, et al. ARID1A and TERT promoter mutations in dedifferentiated meningioma. Cancer genetics 2015;208:345-50.
29. Abedalthagafi MS, Merrill PH, Bi WL, et al. Angiomatous meningiomas have a distinct genetic profile with multiple chromosomal polysomies including polysomy of chromosome 5. Oncotarget 2014;5:10596-606.
30. Lamszus K LU, Schmidt NO, Stavrou D, Ergun S, Westphal M. Vascular endothelial growth factor, hepatocyte growth factor/scatter factor, basic fibroblast growth factor, and placenta growth factor in human meningiomas and their relation to angiogenesis and malignancy. Neurosurgery 2000;46:938-47
31. Zhu XP LK, Kamaly-Asl ID, et al. Quantification of endothelial permeability, leakage space, and blood volume in brain tumors using combined T1 and T2* contrast-enhanced dynamic MR imaging. J Magn Reson Imaging 2000;11:575-85.
32. Sorensen AG BT, Zhang WT, et al. A "vascular normalization index" as potential mechanistic biomarker to predict survival after a single dose of cediranib in recurrent glioblastoma patients. Cancer Res 2009;69:5296-300.
33. Hawighorst H ER, Knopp MV, et al. Intracranial meningeomas: time- and dose-dependent effects of irradiation on tumor microcirculation monitored by dynamic MR imaging. Magn Reson Imaging 1997;15:423-32.
34. Ludemann L GW, Wurm R, Wust P, Zimmer C. Quantitative measurement of leakage volume and permeability in gliomas, meningiomas and brain metastases with dynamic contrast-enhanced MRI. Magn Reson Imaging 2005;23:833-41.
35. Yang S LM, Zagzag D, et al. Dynamic contrast-enhanced perfusion MR imaging measurements of endothelial permeability: differentiation between atypical and typical meningiomas. AJNR Am J Neuroradiol 2003;24:1554-9.
36. Batchelor TT SA, di Tomaso E, et al. AZD2171, a pan-VEGF receptor tyrosine kinase inhibitor, normalizes tumor vasculature and alleviates edema in glioblastoma patients. Cancer Cell 2007;11:83-95.
37. Sorensen AG EK, Polaskova P, et al. Increased survival of glioblastoma patients who respond to antiangiogenic therapy with elevated blood perfusion. Cancer Res 2012;72:402-7.
38. Gerstner ER CP, Wen PY, Jain RK, Batchelor TT, Sorensen G. Infiltrative patterns of

glioblastoma spread detected via diffusion MRI after treatment with cediranib. Neuro Oncol 2010;12:466-72.
39. Batchelor TT GE, Emblem KE, et al. Improved tumor oxygenation and survival in glioblastoma patients who show increased blood perfusion after cediranib and chemoradiation. Proc Natl Acad Sci U S A 2013;110:19059-64.
40. Brahmer JR, Tykodi SS, Chow LQ, et al. Safety and activity of anti-PD-L1 antibody in patients with advanced cancer. N Engl J Med 2012;366:2455-65.
41. Macdonald DR, Cascino TL, Schold SC, Jr., Cairncross JG. Response criteria for phase II studies of supratentorial malignant glioma. J Clin Oncol 1990;8:1277-80.
42. Wolchok JD, Hoos A, O'Day S, et al. Guidelines for the evaluation of immune therapy activity in solid tumors: immune-related response criteria. Clin Cancer Res 2009;15:7412-20.
43. Kaley T, Barani I, Chamberlain M, et al. Historical benchmarks for medical therapy trials in surgery- and radiation-refractory meningioma: a RANO review. Neuro Oncol 2014;16:829-40.
44. Norden AD, Ligon KL, Hammond SN, et al. Phase II study of monthly pasireotide LAR (SOM230C) for recurrent or progressive meningioma. Neurology 2015;84:280-6.
45. Wen PY, Yung WK, Lamborn KR, et al. Phase II study of imatinib mesylate for recurrent meningiomas (North American Brain Tumor Consortium study 01-08). Neuro Oncol 2009;11:853-60.
46. Norden AD, Raizer JJ, Abrey LE, et al. Phase II trials of erlotinib or gefitinib in patients with recurrent meningioma. J Neurooncol 2010;96:211-7.

APPENDIX A PERFORMANCE STATUS CRITERIA

**ECOG Performance Status Scale**		**Karnofsky Performance Scale**	
Grade	Descriptions	Percent	Description
0	Normal activity. Fully active, able to carry on all pre-disease performance without restriction.	100	Normal, no complaints, no evidence of disease.
		90	Able to carry on normal activity; minor signs or symptoms of disease.
1	Symptoms, but ambulatory. Restricted in physically strenuous activity, but ambulatory and able to carry out work of a light or sedentary nature (*e.g.*, light housework, office work).	80	Normal activity with effort; some signs or symptoms of disease.
		70	Cares for self, unable to carry on normal activity or to do active work.
2	In bed <50% of the time. Ambulatory and capable of all self-care, but unable to carry out any work activities. Up and about more than 50% of waking hours.	60	Requires occasional assistance, but is able to care for most of his/her needs.
		50	Requires considerable assistance and frequent medical care.
3	In bed >50% of the time. Capable of only limited self-care, confined to bed or chair more than 50% of waking hours.	40	Disabled, requires special care and assistance.
		30	Severely disabled, hospitalization indicated. Death not imminent.
4	100% bedridden. Completely disabled. Cannot carry on any self-care. Totally confined to bed or chair.	20	Very sick, hospitalization indicated. Death not imminent.
		10	Moribund, fatal processes progressing rapidly.
5	Dead.	0	Dead.

APPENDIX B Event of clinical interest guidance document
