# Supplementary material for: Phase 2 study of pembrolizumab in patients with recurrent and residual high-grade meningiomas
Source: Nat Commun. 2022 Mar 14;13:1325. doi: 10.1038/s41467-022-29052-7 (PMC8921328; doi:10.1038/s41467-022-29052-7)
Supplement: Supplementary file 2 — Reporting Summary [file 41467_2022_29052_MOESM2_ESM.pdf]

nature research

Corresponding author(s): Brastianos

Last updated by author(s): Jan 12, 2022

Reporting Summary

Nature Research wishes to improve the reproducibility of the work that we publish. This form provides structure for consistency and transparency in reporting. For further information on Nature Research policies, see our Editorial Policies and the Editorial Policy Checklist.

Statistics

For all statistical analyses, confirm that the following items are present in the figure legend, table legend, main text, or Methods section.

n/a	Confirmed	
☐	☒	The exact sample size (*n*) for each experimental group/condition, given as a discrete number and unit of measurement
☒	☐	A statement on whether measurements were taken from distinct samples or whether the same sample was measured repeatedly
☐	☒	The statistical test(s) used AND whether they are one- or two-sided<br>*Only common tests should be described solely by name; describe more complex techniques in the Methods section.*
☒	☐	A description of all covariates tested
☒	☐	A description of any assumptions or corrections, such as tests of normality and adjustment for multiple comparisons
☐	☒	A full description of the statistical parameters including central tendency (e.g. means) or other basic estimates (e.g. regression coefficient) AND variation (e.g. standard deviation) or associated estimates of uncertainty (e.g. confidence intervals)
☐	☒	For null hypothesis testing, the test statistic (e.g. *F*, *t*, *r*) with confidence intervals, effect sizes, degrees of freedom and *P* value noted<br>*Give P values as exact values whenever suitable.*
☒	☐	For Bayesian analysis, information on the choice of priors and Markov chain Monte Carlo settings
☒	☐	For hierarchical and complex designs, identification of the appropriate level for tests and full reporting of outcomes
☒	☐	Estimates of effect sizes (e.g. Cohen's *d*, Pearson's *r*), indicating how they were calculated

Our web collection on statistics for biologists contains articles on many of the points above.

Software and code

Policy information about availability of computer code

Data collection	No software was used.
Data analysis	Commercially available statistical software packages (Stata version 16) was used for data analysis. A deep-learning algorithm (DeepNeuro) was used to initially segment the meningioma on post-contrast MRI's. These segmentations were edited by a board-certified neuro-oncologist and used in downstream imaging analyses.

For manuscripts utilizing custom algorithms or software that are central to the research but not yet described in published literature, software must be made available to editors and reviewers. We strongly encourage code deposition in a community repository (e.g. GitHub). See the Nature Research guidelines for submitting code & software for further information.

Data

Policy information about availability of data

All manuscripts must include a data availability statement. This statement should provide the following information, where applicable:

- Accession codes, unique identifiers, or web links for publicly available datasets
- A list of figures that have associated raw data
- A description of any restrictions on data availability

The raw clinical and imaging data are protected due to patient privacy laws. Information is taken directly from the electronic medical record or original source generated by treating investigators (e.g. email confirmations, Adverse Event logs). This is stored on a secured network drive to which only appropriately trained and delegated staff have access to. Lesion measurements are obtained from the Tumor Metrics Imaging Core online portal that uses a secure server to which only appropriately trained and delegated staff are granted access to. Any requests for raw and analyzed data should be sent in writing to Priscilla Brastianos (pbrastianos@mgh.harvard.edu) and will be reviewed by the DF/HCC Institutional Review Board (IRB) in an expeditious fashion. Patient-related data not included in the paper were generated as part of a clinical trial and are subject to patient confidentiality. Any data and materials (e.g. tissue samples, PD-L1 testing, or imaging

data) that can be shared will need approval from the DF/HCC IRB and a Material Transfer Agreement in place. Deidentified data then will then be transferred to the inquiring investigator in an expeditious fashion over secure file transfer. The study protocol, statistical analysis plan, and Source Data for the figures in this paper are included with the submission.

Field-specific reporting

Please select the one below that is the best fit for your research. If you are not sure, read the appropriate sections before making your selection.

- [x] Life sciences
- [ ] Behavioural & social sciences
- [ ] Ecological, evolutionary & environmental sciences

For a reference copy of the document with all sections, see nature.com/documents/nr-reporting-summary-flat.pdf

Life sciences study design

All studies must disclose on these points even when the disclosure is negative.

Sample size	The total planned accrual for this clinical trial was 26 patients in order to achieve 24 evaluable patients. The trial was powered to distinguish between PFS-6 rates of 26% vs. 52%. The historical control of PFS-6 was based on a recent meta-analysis showing a historical PFS-6 weighted average of 26% in recurrent grade II/III meningiomas. If at least 10 patients demonstrated PFS-6, among the 24 evaluable patients, the agent would be considered worthy of further study. This design had at least 88% power to detect a true PFS-6 rate of at least 52%, using an exact binomial test with a one-sided significance level of 0.1, against the null hypothesis of 26%.
Data exclusions	No data was excluded.
Replication	Replication of findings could not be performed, as we are reporting pre-specified analysis of a phase II clinical trial.
Randomization	There was no randomization of patients in our study, as this was a single arm phase II study.
Blinding	Blinding was not possible for our study, as this was a single arm phase II study.

Reporting for specific materials, systems and methods

We require information from authors about some types of materials, experimental systems and methods used in many studies. Here, indicate whether each material, system or method listed is relevant to your study. If you are not sure if a list item applies to your research, read the appropriate section before selecting a response.

Materials & experimental systems

n/a	Involved in the study	
[ ]	[x]	Antibodies
[x]	[ ]	Eukaryotic cell lines
[x]	[ ]	Palaeontology and archaeology
[x]	[ ]	Animals and other organisms
[ ]	[x]	Human research participants
[ ]	[x]	Clinical data
[x]	[ ]	Dual use research of concern

Methods

n/a	Involved in the study	
[x]	[ ]	ChIP-seq
[x]	[ ]	Flow cytometry
[x]	[ ]	MRI-based neuroimaging

Antibodies

Antibodies used	Anti-PD-L1 antibody 22C3
Validation	The anti-PDL1 22C3 antibody is a validated CLIA-certified assay for multiple solid tumors for both exploratory and prospective use (Dolled-Filhart M et al., Arch Pathol Lab Med 2016). This assay has been used to assess PD-L1 expression in a number of prior immune checkpoint inhibitor based trials (Garon EB et al., NEJM 2015; Muro et al., Lancet Oncol 2016; Adams S et al., NPJ Breast Cancer 2017; Rodriguez CP et al., Clin Cancer Res 2020; Mehnert JM et al., BMC Cancer 2019)

Human research participants

Policy information about studies involving human research participants

Population characteristics	Eligible patients had histologically confirmed progressive or residual intracranial or metastatic meningioma. 26 patients were enrolled. 15 patients were male and 11 were female. 20 patients were White, 3 were Black, 2 were 'Other', and 1 identified with more than one race. The median age of patients in our cohort was 61.4, and the range was 19 to 89 years of age.<br><br>Patients must have had progressive or residual measurable disease immediately prior to enrollment. Progressive disease was defined as an increase in size of a measurable meningioma lesion on imaging by greater than 25% (bidirectional area). The change must occur between scans separated by no more than 24 months. Residual measurable disease was defined by measurable lesions with clearly defined margins by MRI scans, with a minimum diameter of 10mm in one dimension.<br><br>Prior meningioma-directed therapies, such as radiotherapy and systemic therapies, were strongly preferred but not required. To minimize the risk of enrolling patients with pseudo-progression, patients with prior external beam radiation therapy or interstitial brachytherapy were required to show evidence of progressive meningioma within the irradiated field > 24 weeks after completion of radiation treatment. Participants who had systemic therapies within 2 weeks prior to trial enrollment were excluded. Concurrent meningioma-directed systemic agents or radiation with the study drug was not allowed. Other key inclusion criteria included the following: age > 18, ECOG performance status < 2, and stable dose of dexamethasone at 2 mg or less for at least 7 days prior to start of trial. Key exclusion criteria included: the presence of brainstem meningiomas and active autoimmune disease that required systemic immunosuppression within two years of enrollment.
Recruitment	Patients were recruited through the Massachusetts General Hospital Pappas Center for Neuro-Oncology (MGH) and Dana-Farber Center for Neuro-Oncology (DFCI). In addition, the MGH and DFCI have a large referral base encompassing the New England area through which eligible patients were identified and recruited. While steps were taken to minimize bias in our study in accordance to MGH & DFCI guidelines, one bias is that the majority of our cohort (20/26; 76.9%) is White, consistent with the population in the New England area.
Ethics oversight	The Dana-Farber Harvard Cancer Center (DF/HCC) Institutional Review Board approved the protocol.

Note that full information on the approval of the study protocol must also be provided in the manuscript.

Clinical data

Policy information about clinical studies

All manuscripts should comply with the ICMJEguidelines for publication of clinical research and a completedCONSORT checklist must be included with all submissions.

Clinical trial registration	Clinicaltrials.gov identifier NCT03279692
Study protocol	The full clinical trial protocol was provided with manuscript submission.
Data collection	Study data files for 26 patients were retrieved for this report on November 11, 2021. The first patient was enrolled on November 7, 2017 and the last patient for this analysis was enrolled on January 8, 2021. Clinical research coordinators and physicians collected salient clinical information for each patient. Information was taken directly from the electronic medical record or original source generated by treating investigators (e.g. email confirmations, Adverse Event logs). This data was then stored on a secured network drive and the Inform Database (Oracle; version 6.2) to which only appropriately trained and delegated staff have access to. Lesion measurements on MRI are obtained from the Tumor Metrics Imaging Core (TIMC) online portal that utilizes a secure server to which only appropriately trained and delegated staff are gained access to. All collected data were then sent to the Dana-Farber/Harvard Cancer Center Office of Data Quality for quality control before analysis.
Outcomes	The primary endpoint was the proportion of patients alive and progression-free at 6 months (PFS-6). Secondary endpoints include overall survival (OS), progression-free survival (PFS), best intracranial response (as defined by complete response [CR], partial response [PR], stable disease [SD], or progressive disease [PD]), and toxicity using Common Terminology Criteria for Adverse Events (CTCAE) version 5.0.
